# Modeling spatial variation in risk of presence and insecticide resistance for malaria vectors in Laos

**DOI:** 10.1371/journal.pone.0177274

**Published:** 2017-05-11

**Authors:** Marc Souris, Sébastien Marcombe, Julie Laforet, Paul T. Brey, Vincent Corbel, Hans J. Overgaard

**Affiliations:** 1 UMR "Émergence des Pathologies Virales" (EPV: Aix-Marseille Univ–IRD 190 –Inserm 1207 –EHESP–IHU Méditerranée Infection), Marseille, France; 2 Asian Institute of Technology, Remote Sensing and GIS FoS, Klong Luang, Pathumthani, Thailand; 3 Institut Pasteur du Laos, Vientiane, Lao PDR; 4 Université de Toulouse–Le Mirail, Département de Géographie & Aménagement—UFR Sciences, Espaces, Sociétés Université, Toulouse, France; 5 UMR « Maladies Infectieuses et Vecteurs, Ecologie, Génétique, Evolution et Contrôle » (MIVEGEC, UM1-CNRS 5290-IRD 224), Montpellier, France; 6 Kasetsart University, Department of Entomology, Faculty of Agriculture, Bangkok, Thailand; 7 Faculty of Science and Technology, Norwegian University of Life Sciences, Ås, Norway; New Mexico State University, UNITED STATES

## Abstract

Climatic, sociological and environmental conditions are known to affect the spatial distribution of malaria vectors and disease transmission. Intensive use of insecticides in the agricultural and public health sectors exerts a strong selective pressure on resistance genes in malaria vectors. Spatio-temporal models of favorable conditions for Anopheles species’ presence were developed to estimate the probability of presence of malaria vectors and insecticide resistance in Lao PDR. These models were based on environmental and meteorological conditions, and demographic factors. GIS software was used to build and manage a spatial database with data collected from various geographic information providers. GIS was also used to build and run the models. Results showed that potential insecticide use and therefore the probability of resistance to insecticide is greater in the southwestern part of the country, specifically in Champasack province and where malaria incidence is already known to be high. These findings can help national authorities to implement targeted and effective vector control strategies for malaria prevention and elimination among populations most at risk. Results can also be used to focus the insecticide resistance surveillance in *Anopheles* mosquito populations in more restricted area, reducing the area of surveys, and making the implementation of surveillance system for *Anopheles* mosquito insecticide resistance possible.

## 1. Introduction

**Fig 1:**
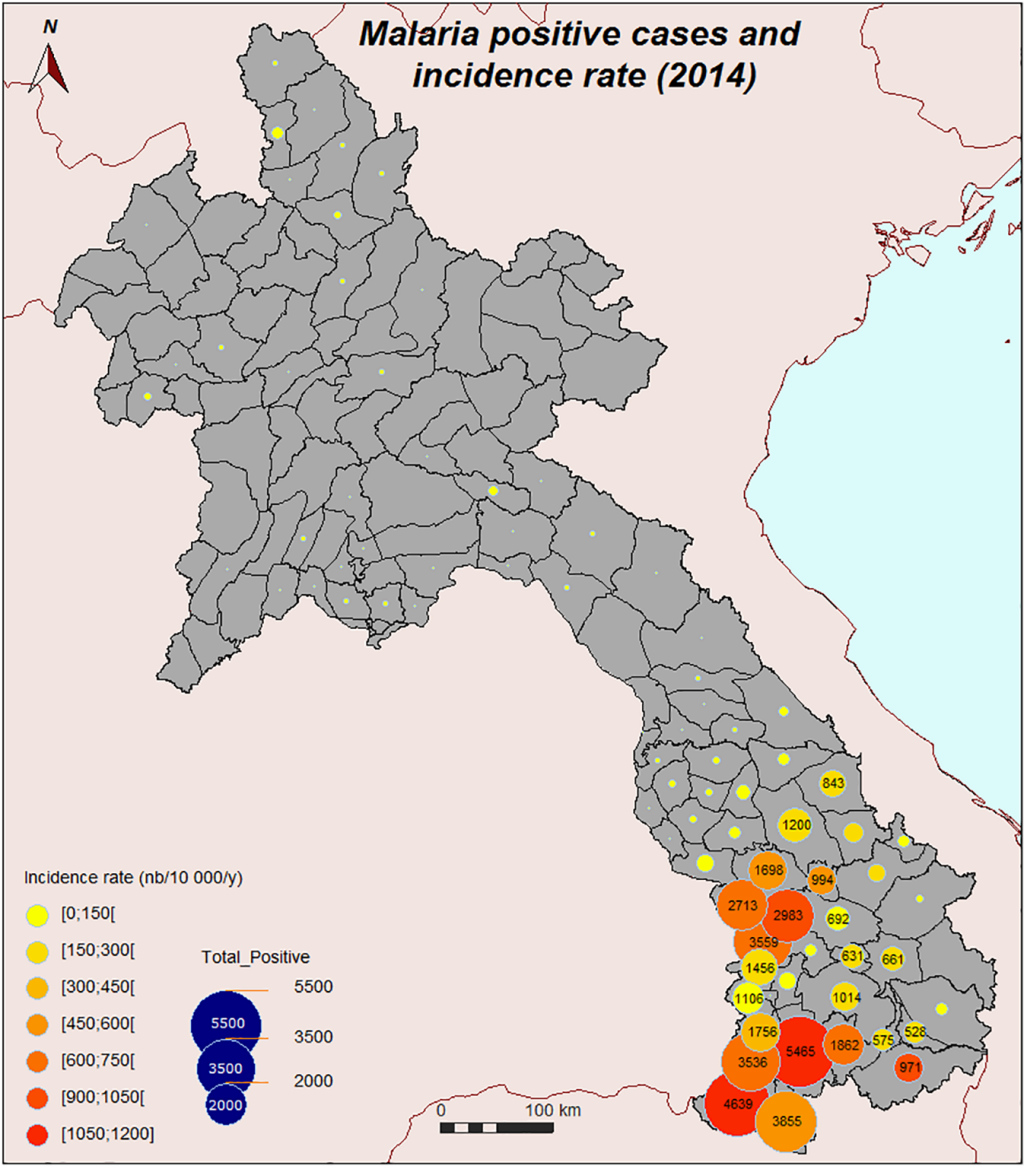
Malaria positive cases and incidence rate, 2014 (from CMPE Malaria Information System).

Malaria is still an important health issue in the Lao PDR (hereafter Laos). In 2010, 63% of the Lao population lived in rural areas and many were therefore directly affected by this mainly rural infectious disease [1]. Although malaria incidence in Laos decreased by 50% between 2000 and 2010, the case incidence has increased since 2011, with 38,131 malaria confirmed cases in 2014 by the national authorities (CMPE, the Center for Malariology, Parasitology and Entomology, Ministry of Health, Lao PDR). Malaria is endemic in Laos, but highly heterogeneous, with more intense transmission in remote and forested areas: Malaria incidence is highest in southern Laos and lowest in the northern part of the country where transmission is sporadic and local (Fig 1) [2–3]. Malaria outbreaks have been observed since 2011 in the five southern provinces: Savannakhet, Saravane, Sekong, Champasack, and Attapeu (CMPE Malaria Information System, unpublished observations).

Malaria vector control in Laos was first initiated in 1953 with indoor residual spraying (IRS) of DDT twice a year at 2gm/m2 [4] and carried out along with extensive epidemiological and entomological surveys. IRS with DDT continued until the revolution in 1975 and was definitively banned in 1990. In Vientiane province mass drug administration (MDA) took place between 1969 and 1988, with a break between 1975 and 1977. After the ban of DDT and termination of the MDA program, insecticide-treated bed-nets (ITNs) and long lasting insecticidal nets (LLINs) were introduced in the 2000’, first in Borikhamxay province, then in Luang Pra Bang, Saravane, Vientiane and Savannakhet provinces. According to the WHO, about 280,000 LLINs were distributed to populations in 2014 hence representing a net coverage of 20% of the population [2].

Intensive use of insecticides by agricultural pest control and insecticide used in vector control may exert a strong selection pressure for resistance development in malaria vectors [5–9]. Insecticide resistance arises when the frequency of resistance genes in the mosquito population increases after being exposed to insecticides. The Asian market represents 22% of the global market of pesticides [4]. In northern Thailand, it was found that pesticides used for crop protection was significantly correlated with the presence of insecticide resistance in malaria vectors [10].

The risk of insecticide resistance in malaria vectors in South-East Asia represents a potential threat for malaria control and the achievements seen during recent years. Insecticide resistance in major malaria vectors have been detected in neighboring countries such as Cambodia, Vietnam, Thailand [11–12] and it is important for National Malaria Control Program to identify suitable areas for insecticide resistance emergence. This information is essential to implement national plans for insecticide resistance management.

In practice, it is difficult to assess favorable ecological conditions for malaria vector presence. The assessment of the spatial distribution of such favorable conditions is also difficult, especially when a spatial precision consistent with local actions of vector control is needed. Indeed, entomological surveys to assess mosquito presence cannot reasonably be conducted over a wide area. The statistical spatial representativeness of a collection point (i.e. the variability of the result in the neighborhood of a collection point) is difficult to assess and varies in space and time, and depends on species and environmental and climatic conditions. The presence and the abundance of the vector is dependent on many geographical, climatic, environmental and anthropogenic variables [13–20]. Results also vary considerably over time depending on seasonal, annual, and environmental conditions that influence the presence of these vectors. Most studies assessing mosquito vector presence and abundance do not exceed the village level [21]. All other studies generally use a model to estimate the probability of the potential presence or average abundance of the vector according to geographical and/or environmental variables (land use, temperature, topography, vegetation, habitat, vector control actions, etc.) [22–25]. For example, the MAP project website shows potential presence maps for many *Anopheles* complex species, derived from a Bayesian geostatistical modeling approach (http://www.map.ox.ac.uk/map/).

The objective of this work was to develop predictive spatial models that estimate with higher spatial accuracy the probability of presence of malaria vectors and insecticide resistance in Laos. Here a modeling approach with high spatial accuracy was used to estimates the location of favorable conditions for malaria vector presence and insecticide resistance, based on previous studies, expert knowledge of entomologists and agronomists and taking into account the vulnerability of people to these hazards [12] [19] [26–37]. The general model uses three sub-models, one to estimate areas where conditions are favorable for the development of vectors, the second to estimate the likelihood of insecticide resistance, and the third to estimate the vulnerability of the population to these hazards.

The main expected outcomes are accurate spatial distributions of risk of vulnerable human hosts being exposed to malaria vectors, risk of malaria vectors being exposed to insecticides and becoming resistant, and risk of vulnerable human hosts being exposed to potentially resistant malaria vectors. Accurate space-time distributions of favorable conditions for malaria vectors are also a valuable intermediate outcome. The results are expected to help public health authorities to improve insecticide resistance surveillance by targeting specific and sensitive areas.

## 2. Materials and methods

### 2.1 Study area

**Fig 2:**
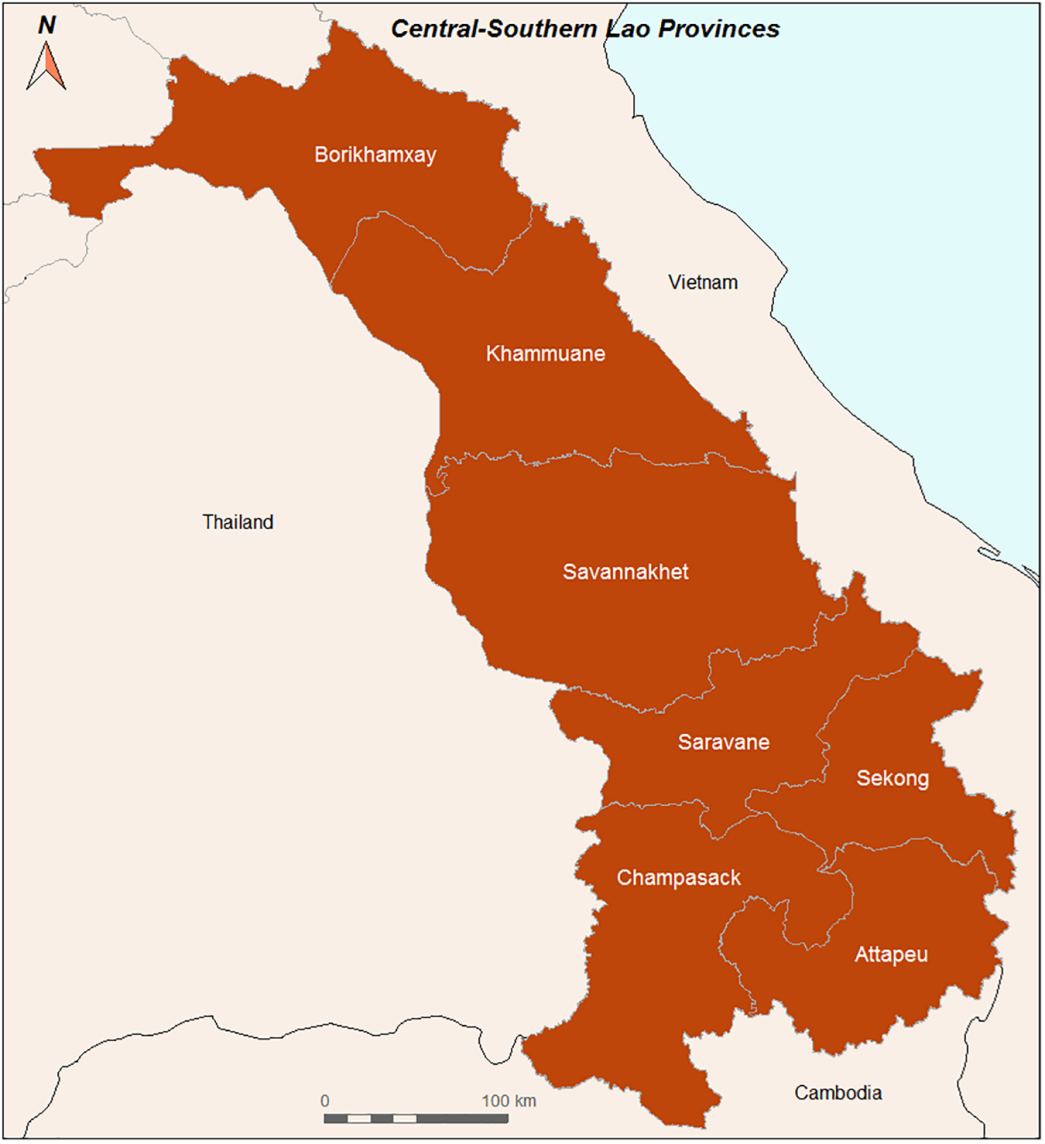
Central and southern Lao provinces.

This study comprises the whole Laos, but because land-use data from the northern part of Laos dates back to 2002 and data from 2014 are still not available, specific focus was put on seven central and southern provinces (Borikhamxay, Khammuane, Savannakhet, Champasack, Attapeu, Sekong and Saravane) (Fig 2). As previously mentioned, these provinces are at higher risk of malaria.

### 2.2 Data collection and geodatabase

**Fig 3:**
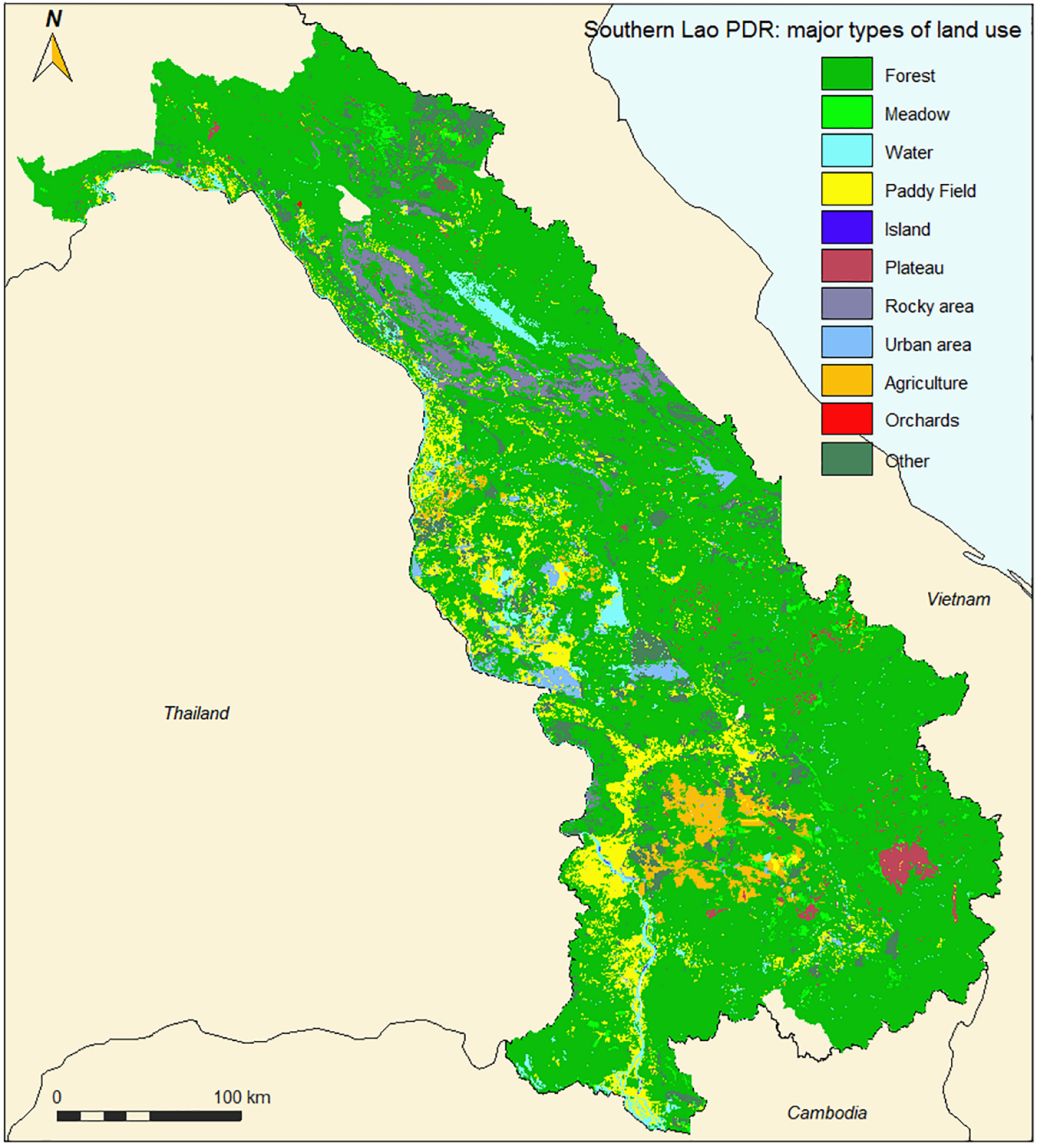
Land-use, 2014, central and southern Laos (from DALAM data).

We used environmental and demographical data verified and managed by a geographic information system (GIS). Data on administrative borders (provinces, districts), village locations, demography, poverty, precipitation, temperature, relative humidity, land-use, hydrology, topography, malaria cases, and bed-net coverage were collected from various sources. Many of these factors potentially affect the presence of malaria vectors and development of insecticide resistance in mosquitoes. We only used already available environmental data, from local Lao administrative sources (for land-use, demography, and epidemiology) or international data providers (for climate, topography). The following data sets were acquired and used in the analysis:

- Land-use from 2014 (Fig 3) was acquired from the Department of Land Management and Development (DALaM, Ministry of Agriculture and Forestry). The land use data were originally classified from remote sensing images (with a resolution of 5 m). The quality of these data was verified and any detected errors were corrected.
- Administrative borders and data on demographical characteristics and poverty were retrieved from the Lao DECIDE project (http://www.decide.la/en/), a follow-up of the “Lao Poverty Mapping and Socio-economic Atlas of Lao PDR project” (2006–2009). These projects were developed in the framework of a collaboration between Lao Statistics Bureau, Ministry of Natural resources and Environment, Ministry of Agriculture and Forestry, and the Centre for Development and Environment at the University of Bern in Switzerland [38,39].
- The Lao Ministry of Agriculture and Forestry provided list and geographical coordinates of villages.
- Climate data such as monthly and annual temperature and rainfall averages were downloaded from WorldClim—Global Climate Data (http://worldclim.org). The raster data have a resolution of 1 km^2^.
- The Center for Malaria Parasitology and Entomology (CMPE) provided data on malaria cases and bed-net coverage by district.
- Digital Elevation Models were created with GIS at various scales (100 m, 200 m 400 m per pixel) from SRTM-4.0 raster data (Shuttle Radar Topographic Mission, NASA, 90 m per pixel. http://www2.jpl.nasa.gov/srtm/).

Data were integrated into a geodatabase as layers with attributes, each layer with detailed metadata. The GIS geodatabase contained administrative layers (locations of villages with information on the presence of health facilities, boundaries of villages with population, boundaries of districts and provinces), climate layers (temperature, precipitation, relative humidity), land-use (2002 and 2014), and epidemiological data on malaria. Bed-net coverage was given by district as number of people protected, percentage of people protected, number of bed-nets, and number of bed-net per person. Malaria epidemiological data by district included number of people tested by RDT, number of malaria confirmed cases, and number of deaths due to malaria. GIS database can be downloaded at http://doi.org/10.5281/zenodo.570063 [40].

### 2.3 Data processing and modeling

**Fig 4:**
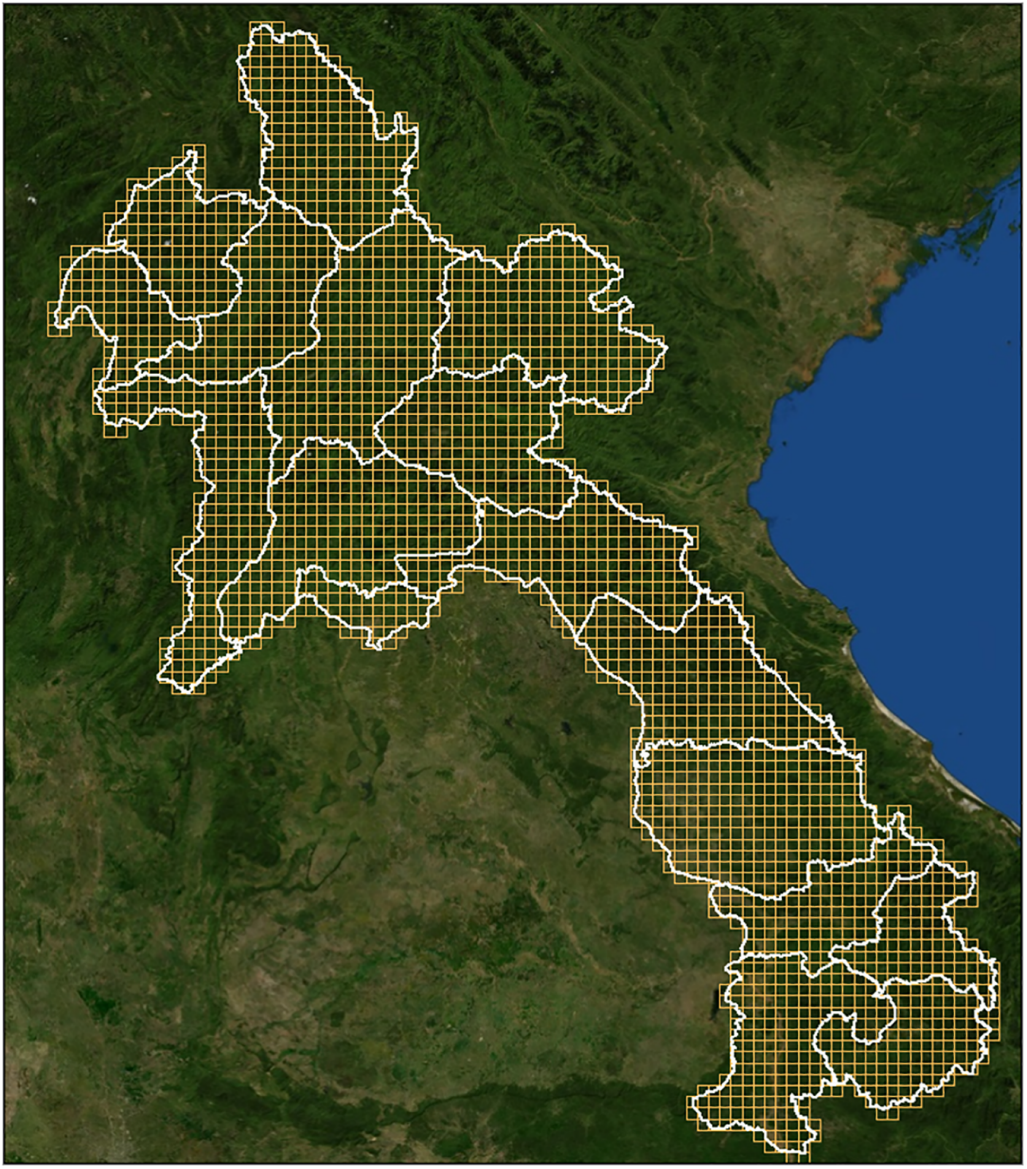
Grid of 10×10 km cells as geographical units.

Risk of *Anopheles* mosquito presence and insecticide resistance was estimated by cells in a grid covering the study area. The spatial definition of the grid (the size of the cells) is based on the objectives of the study (in term of shape and spatial accuracy) and the availability of environmental data needed to characterize the cells of the grid. Thus, to present results with enough precision to manage local phenomena, we created a 10×10 km cell grid, giving a total of 2,836 cells for the whole country and 1,071 cells for the central and southern region (Fig 4).

We developed models combining variables describing these cells. Mean temperature, rainfall, altitude, percentage of surface for each land use type, total of malaria cases, total population were calculated for each grid cell by GIS process. The vulnerability of the human population and its exposure to mosquito presence and potential insecticide resistance in mosquitoes were also estimated by these geographical units. Results were calculated for each grid cell. Results were also calculated for administrative units (village boundaries and districts) by spatial integration from cells.

We used maps to show the results of spatial distributions. We present maps using smoothed trend surface from interpolation rather than results by cells in order to give greater readability.

Three sub-models (Table 1) have been developed and are used for the models for risk evaluation (Table 2: risk of vulnerable human hosts being exposed to malaria vectors, risk of malaria vectors being exposed to insecticides and becoming resistant, and risk of vulnerable human hosts being exposed to potentially resistant malaria vectors): 1) a sub-model of *Anopheles* presence probability from favorable environmental conditions for *Anopheles* mosquitoes; 2) a sub-model of insecticide presence probability, from suitable conditions for insecticide use; 3) a sub-model to estimate the vulnerability of the human population.

**Table 1:** Three sub-models have been developed from environmental, climatic and demographic data.

Sub-models	Based on
Y4: Probability of *Anopheles* mosquito presence	Y1: Presence, based on land use
	Y2: Presence, based on temperature
	Y3: Presence, based on rainfall and topography
Y7: Probability of insecticide use	Y5: Insecticide use based on land use
	Y6: Density of ITNs
Y10: Vulnerability of the human population	Y8: Human population density and poverty
	Y9: Inverse density of ITNs

**Table 2:** Risk evaluation models build on the top of the three sub-models Y4, Y7, Y10.

Models for Risk evaluation	Components	Based on
Y11: Risk of vulnerable human hosts being exposed to malaria vectors	Probability of *Anopheles* mosquito presence × Vulnerability of the human population	Y4, Y10
Y12: Risk of malaria vectors being exposed to insecticides and becoming resistant	Probability of *Anopheles* mosquito presence × Probability of insecticide use	Y4, Y7
Y13: Risk of vulnerable human hosts being exposed to potentially resistant malaria vectors	Resistance risk × Population vulnerability	Y12, Y10

#### 2.3.1 Sub-model 1: Probability of *Anopheles* presence, from favorable environmental conditions

This sub-model aims to estimate the space-time distribution of favorable environmental conditions for the three primary malaria vectors in Laos: *An*. *dirus* s.l., *An*. *minimus* s.1. and *An*. *maculatus* s.l. The model is based on the following environmental factors only: land use, climate (temperature and rainfall), and topography. Knowledge from literature and experts was used to assess relationships between environmental variables and their suitability for the development of malaria vectors.

**2.3.1.1. Land use:** In Southeast Asia, land use is considered as the major environmental factor influencing *Anopheles* distribution, followed by temperature and precipitation [13–14]. Tropical forests, forest fringes, and areas around wetlands and water bodies are favorable areas for *Anopheles* mosquitoes [30], but *Anopheles* can also be found close to agricultural lands such as rice paddy fields, wood cultures, and orchards [14][41]. To quantify the suitability of each land use type *i* for the presence of each species, a weight *a*_*i*_, from 0 to 10 was given to each land use type (Table 3, established from literature review and expert knowledge).

**Table 3:** Estimation of likelihood of presence of *An*. *dirus* s.l., *An*. *maculatus* s.l., and *An*. *minimus* s.l. and insecticide use for agriculture, in different land-use types. Scores for mosquito presence are from 0 to 10, with 0 the lowest and 10 the highest. Scores for insecticide use is from 1 to 4, with 1 the lowest and 4 the highest insecticide use. Land use types and codes are taken from the land use classification used by the Ministry of Agriculture.

Code	Land use type	Mosquito presence				Insecticide use^b^
		*An*. *dirus*	*An*. *maculatus*	*An*. *minimus*	References^a^	
11	Evergreen Forest	10	7	8	[13,14,26,33–37]	1
13	Mixed deciduous Forest	10	7	8	[13,14,26,33–37]	1
15	Dry Dipterocarp Forest	10	7	8	[13,14,26,33–37]	1
16	Gallery Forest	3	9	10	[13,14,26,32–37,41]	1
17	Coniferous forest	1	1	1	[26,29–37]	1
18	Mixed Broad-Leaved Forest	10	9	9	[13,14,26,33–37]	1
19	Forest Plantation	6	7	7	[13,14,26,33–37]	1
191	Rubber	8	6	8	[30,36]	2
192	Eucalyptus	5	5	5		2
193	Kathin nalong wood	6	6	6		1
194	Teak wood	6	6	6		2
195	Aquilaria crassna wood	8	6	8		3
21	Bamboo Forest	7	7	7	[13,14,26,33–37]	1
22	Unstocked Forest	1	1	1	[13,14,26,33–37]	1
24	Plateau	2	2	2		1
31	Savannah	1	1	1		1
32	Scrubland	4	4	4	[31]	1
41	Rice Paddy	2	7	7	[13,14,26,33–37,38]	3
411	Irrigated Paddy	1	7	7	[13,14,26,33–37,38]	3
42	Other Agriculture	3	3	3	[13,26]	2
421	Cassava	1	1	4	[37]	1
422	Sugarcane	1	1	4	[37]	1
423	Morinda citrifolia	1	1	1		4
424	Coffee	2	2	2	[37]	2
425	Maize	1	1	1		2
426	Hibiscus sabdariffa	1	1	1		4
427	Bastard Cardamom	1	1	1		2
428	Cotton	1	1	1		2
429	Tea	1	1	1		2
430	Mulberry	2	2	2	[37]	4
431	Job's Tear	1	1	1		4
432	Coconut	1	1	1		4
434	Banana	1	1	1		4
435	Sweet Potato	1	1	1		2
436	Groundnut	1	1	1		3
437	Citrus	2	3	3	[26,33,37]	4
440	Other Fruit tree	2	3	3	[26,33,37]	4
441	Palm	1	1	1		1
51	Rocked	1	1	1		1
52	Grassland	2	4	2	[29,30]	1
521	Pastures	2	4	2	[29,30]	1
53	Wetlands	4	10	8	[29,30]	1
54	Urban area	0	0	0		1
541	Cemetery	1	1	1		1
542	Industrial	0	0	0		1
543	Mine	1	1	1		1
544	Airport	1	1	1		1
545	Stadium (Any Sport)	1	1	1		1
546	Waste dump	1	1	1		1
547	National Protection	10	10	10		1
548	Culture Zone	1	1	1		2
55	Island	4	10	8		1
550	Other concession	1	1	1		1
61	Water body	6	10	8	[29,30]	1
611	Reservoir	2	2	2	[29,30]	1
612	Irrigated area	1	7	7	[29,30]	1
613	Flood area	2	2	2	[29,30]	1
2104	Unpaved Road	1	1	1		1
21011	Paved Road	0	0	0		1
21021	Street Town	0	0	0		1

^a^ References were used as support to assess presence likelihood. If no reference given, presence assessed by authors.

^b^ Scoring based on authors’ assessment, mainly based on Table 7 in [12].

A geo-aggregation by cells from the land use layer was performed in GIS to calculate the proportional cell area, *sp*_*i*_, for each land use type *i* (∑_*i*_ *sp*_*i*_ = 1). We gave the cell a value Y1 (varying from 0 to 1) as a weighted mean (with weights *a*_*i*_) of these *sp*_*i*_ values. The three species (*An*. *dirus*, *An*. *maculatus* and *An*. *minimus*) have slightly different land-use preferences (as shown in Table 3), so three weighted values were calculated per cell:

$$\begin{array}{l} Y1\left[species\right]=\frac{1}{10}\sum_{i}a_{i}sp_{i}\;\mathrm{where}\;a_{i}=\mathrm{weight}\;\mathrm{of}\;\mathrm{land-use}\;\mathrm{type}\;i\;\mathrm{for}\;\mathrm{the}\;\mathrm{species}\\ \;sp_{i}=\left(\mathrm{area}\;\mathrm{of}\;\mathrm{land-use}\;\mathrm{type}\;i\mathrm{in}\;\mathrm{the}\;\mathrm{cell}\right)/\left(\mathrm{total}\;\mathrm{area}\;\mathrm{of}\;\mathrm{cell}\right) \end{array}$$

**2.3.1.2. Temperature and precipitation:** Mosquitoes, as all insects, are ectothermic, i.e. dependent on external temperatures. At critical minimum temperatures, development stops and mosquitoes die or become inactive. As temperatures increase, development and survival increase, until an optimum is reached. As temperatures increase further, insects respond negatively until critical maximum temperatures cause mosquito populations to crash [42]. Optimal temperatures vary from species to species. An operative range for mosquito larval development and survival was assessed for *An*. *stephensi* in experimental incubators resulting in a critical minimum temperature of 15°C and a critical maximum temperature of 36°C, with optimum temperatures between 22–33°C [43]. Since no detailed studies on vector species in Laos or Southeast Asia could be found, the above critical and optimum temperatures were used. Monthly temperature and precipitation averages were used to reflect seasonal variations; results have been produced by month.

Equation Y2 is a probability model for mosquito presence from monthly mean temperatures, T_Month_, using a linear piecewise function:

$$Y2[Month]=0\;if\;T_{Month\;<\;u_{1}}$$

$$Y2[Month]=\frac{T_{Month}-u_{1}}{u_{2}-u_{1}}\;if\;u_{1}\leq T_{Month}<u_{2}$$

$$Y2[Month]=1\;if\;u_{2}\leq T_{Month}<u_{3}$$

$$Y2[Month]=\frac{u_{4}-T_{Month}}{u_{4}-u_{3}}\;if\;u_{3}\leq T_{Month}<u_{4}$$

$$Y2[Month]=0\;if\;T_{Month}\geq u_{4}$$

with u1 = 15°C, u2 = 28°C, u3 = 33°C, u4 = 36°C.

Water is essential for mosquito development. All immature stages of mosquitoes are aquatic. A minimum amount of rainfall is needed to create breeding habitats, but too heavy rainfalls will destroy habitats and flush out eggs and larvae. We assumed limited breeding and mosquito production with precipitation below 40 millimeters of mean rainfall per month, with a progressively increasing likelihood of production until 400 millimeters, and decreasing above.

Equation Y3a is the probability model for mosquito presence from monthly mean rainfall R_Month_. We use again a linear piecewise function:

$$Y3a[Month]=0\;if\;R_{Month<v_{1}}$$

$$Y3a[Month]=\frac{R_{Month}-v_{1}}{v_{2}-v_{1}}\;if\;v_{1}\leq R_{Month}<v_{2}$$

$$Y3a[Month]=\frac{v_{3}-R_{Month}}{v_{3}-v_{2}}\;if\;v_{2}\leq R_{Month}<v_{3}$$

$$Y3a[Month]=0\;if\;R_{Month}\geq v_{3}$$

For monthly mean precipitation: v1 = 40 mm, v2 = 400 mm, v3 = 800 mm.

**Fig 5:**
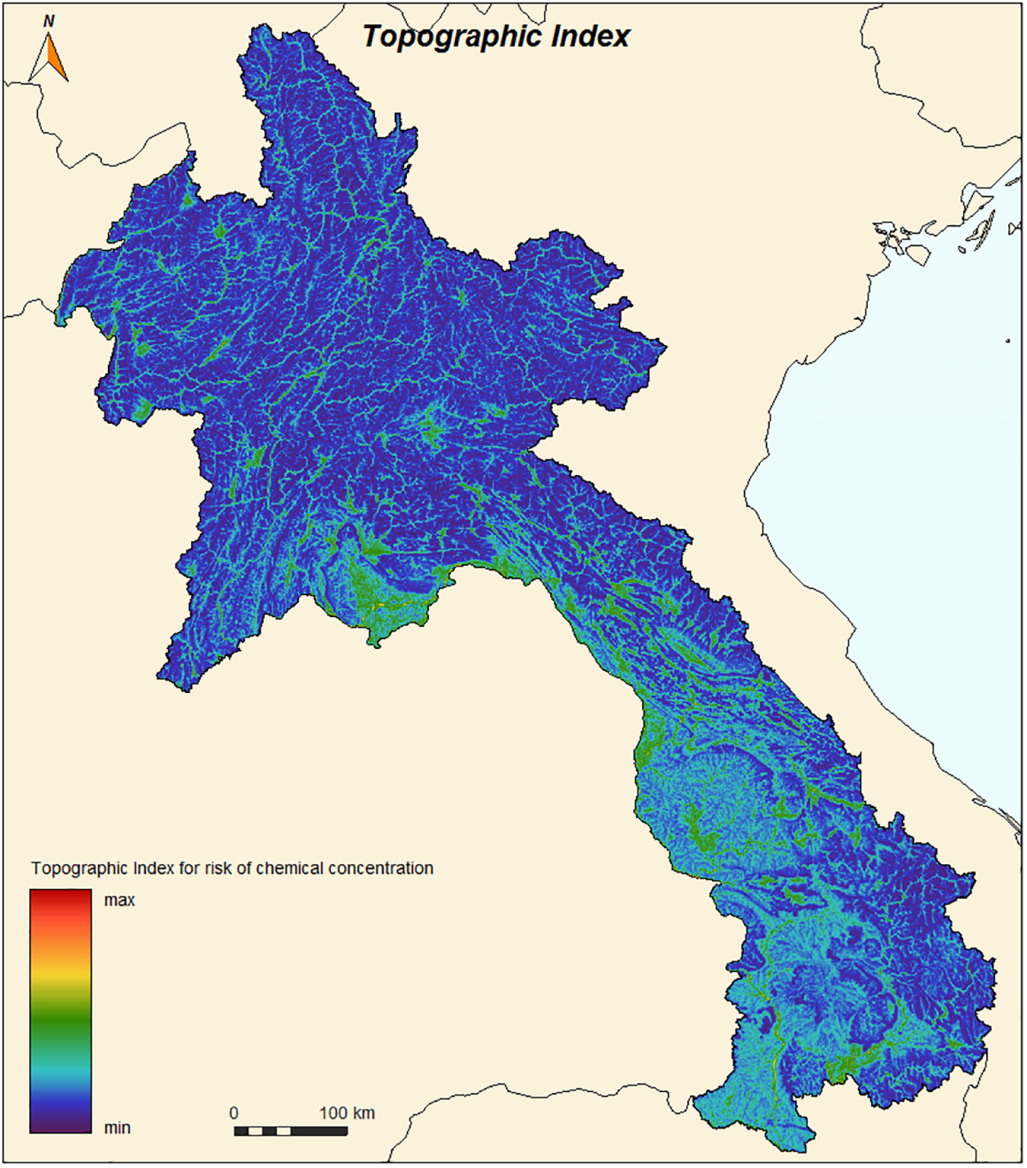
Topographic index TOPMODEL derived from DEM SRTM-4 (90 m resolution).

**2.3.1.3. Topography:** Altitude influences vector density and vector human contact [44]. Altitude is already considered in the model, as temperature is strongly correlated with altitude. Topography also influences the availability and occurrence of larval habitat with sites found commonly in foothills where rainwater can accumulate, next to streams or in the beds of ravines [13]. Using a Digital elevation model (SRTM-4, 90 m resolution), a topographic index (TOPMODEL, based on the amount of water each cell may receive from upstream [45]) was used with GIS to characterize these areas (Fig 5). In each cell, Y3 was calculated from Y3 and this topographic index to consider the capacity of the cell not only to receive but also to accumulate water.

**2.3.1.4. Synthesis:** Land use is a major factor influencing mosquito presence but temperature and precipitation are necessary for mosquito development: our model for the favorable conditions for the presence of *Anopheles* mosquitoes need to be a multiplicative relationship between these three parameters.

The final sub-model for the presence of *Anopheles* mosquitoes from environmental favorable conditions (Y4) is therefore:

Y4[Month,Species]=Y1[Month]×Y2[Month]×Y3[Species]

Depending on the purpose of the study, either the mean or the maximum value over the months can be used, for each species. In the balance of this article, we will also use the mean of the probabilities for the three species, but all results can be obtained by month and specie. In the following equations, Y4 will refer to this value.

#### 2.3.2 Sub-model 2: Probability of insecticide use, from land-use and vector control

Insecticide resistance development in mosquitoes depends on the level of exposure to insecticides. Mosquitoes are mainly exposed to insecticides from agriculture and/or from vector control activities, such as insecticide treated nets (ITNs)–including long lasting insecticide treated nets (LLINs)–or indoor residual spraying (IRS). This sub-model describes the probability of insecticide presence and the intensity of insecticide use. The same principles as the previous sub-model were applied. Weights *b*_*i*_ are given to land use types according to their potential insecticide use (Table 3), based on a previous study conducted in Thailand [12] and expert knowledge. Laos is similar to Thailand in terms of agricultural policies and use, although it may not be as pesticide-intensive as Thailand [4]. It is assumed that agricultural areas, particularly fruit orchards and other cash crops, will apply higher amounts of pesticides than other land uses. This is a pattern commonly seen in neighboring Thailand [10] [12]. We also used expert knowledge from agronomists to develop this sub-model.

First a weighted mean (varying from 0 to 1) was given to each cell:

$$\begin{array}{l} Y5a=1/4\;\sum \left(b_{i}–1\right){\text{sp}}_{i}\;\mathrm{where}\;b_{i}=\mathrm{weight}\;\mathrm{of}\;\mathrm{land}\;\mathrm{use}\;\mathrm{type}\;i\\ \;sp_{i}=\left(\mathrm{area}\;\mathrm{of}\;\mathrm{land-use}\;\mathrm{type}\;i\;\mathrm{in}\;\mathrm{the}\;\mathrm{cell}\right)/\left(\mathrm{total}\;\mathrm{area}\;\mathrm{of}\;\mathrm{cell}\right) \end{array}$$

Leached pesticides are carried downstream following topography and rivers causing accumulation and a higher probability of insecticide presence. In each cell, Y5 was calculated from Y5a and the topographic index, to take into account the capacity of the cell to accumulate pesticides from upstream.

The number of distributed ITNs was only available by district. There are no data on where IRS campaigns were carried out. Density of ITN per person and per cell was calculated in proportion to the population of the cell. Population is available by village, and population of the cell was calculated using integration of villages in cells. Density of ITN per person was then converted to a value between 0 and 1 to reflect the increasing risk of resistance due to ITNs. We estimate that there is no risk of resistance if the density of ITN per person is lower than 0.05, linearly increasing from 0 to 1 until the density reach 0.5, and 1 if the density is greater than 0.5.

Y6=0ifD<0.05

$$Y6=\frac{D-0.05}{0.45}\;if\;0.05\leq D<0.5$$

Y6=1ifD≥0.5

where D is the number of ITN per person for the cell.

Y5 and Y6 are then combined to obtain the model of insecticide resistance risk (equation Y7). We assigned a greater impact of agricultural insecticides since the amounts of insecticides used in agriculture is much larger than what is used in impregnated bed-nets.

Y7=0.9Y5+0.1Y6

#### 2.3.3 Sub-model 3: Vulnerability of the human population

The last sub-model concerns the vulnerability of the Lao population to the presence of malaria vectors. Vulnerability is evaluated independently of the threat: it is related to the factors potentially increasing or reducing susceptibility and local exposure to the threat, even if the threat is not present. Here we consider the vulnerability of each cell using only population density, population socio-economic level and bed net coverage in the cell.

We estimate that the vulnerability of the cell due to population density trend to 1 if population in the cell is superior to 10000 (i.e. if density is higher than 100 inhabitant/km^2^):

Y8a=(2/π)arctan(P/5000)wherePisthenumberofinhabitantinthecell

Poor socio-economic conditions are factors of vulnerably, as poor household condition often implies greater probability of potential exposure to mosquito [46,47]. As indicator of poverty for the cell we use the percentage of population below poverty line inside the cell, derived from the percentage of population below poverty line by village, as calculated by the Lao DECIDE project from the 2005 Population and Housing Census [39]. The higher the poor population, the higher the vulnerability of the cell. We estimate that the vulnerability of the cell due to poor population density trend to 1 if poor population in the cell is superior to 2000:

Y8b=(2./π)×arctan(P’/1000)whereP’isthenumberofinhabitantbelowpovertylineinthecell

We take the mean of Y8a and Y8b as final estimation of vulnerability due to both population density and poor population density, giving more weight to poverty density:

Y8=(Y8a+3×Y8b)/4

ITNs and IRS may reduce population vulnerability since they are able to reduce the contact between humans and mosquito populations. The number of bed net per person from sub-model 2 was used but reversing the score, i.e. the more mosquito bed nets per person, the more people are protected, and the less vulnerable is the cell. We limit the reducing factor Y9 to 0.25, even if bed net coverage is superior to 1 bednet for 2 people: not every people is using bednet even if they have one.

Y9=1ifD<0.05

$$Y9=1-\frac{D-0.05\;}{0.6}\;if\;0.05\leq D<0.5$$

Y9=0.25ifD≥0.5

where D is the number of ITN per person for the cell.

These two values are combined to obtain a final estimation of vulnerability for the cell (equation Y10):

Y10=Y8×Y9

#### 2.3.4 Modeling risks–main models

Modeling risks involve combining vulnerable hosts exposed to the threats, as follows:

1. *Risk of vulnerable hosts to be exposed to malaria vectors*:
  Probability of *Anopheles* mosquito presence × Population Vulnerability: Y11 = Y4 × Y10
2. *Risk of malaria vectors being exposed to insecticide and becoming resistant to insecticide*:
  Probability of *Anopheles* mosquito presence × Probability of insecticide use: Y12 = Y4 × Y7
3. *Risk of vulnerable hosts to be exposed to potentially resistant malaria vectors*:
  Risk of malaria vectors being exposed to insecticide and becoming resistant to insecticide × Population Vulnerability: Y13 = Y12 × Y10

### 2.4. Software

Data verification and editing, data management, GIS data analysis and modeling have been performed with SavGIS software. GIS Software, GIS database used in this work is available on SavGIS website (www.savgis.org). SavGIS macro-command recording all GIS commands used in this work is available as S1 Macro.

## 3. Results

Only results related to variables used in the sub-models (land use, temperature, rainfall), to the sub-models themselves, and to the risk models are presented here. Other results, such as results by district (S1 Table), and maps showing monthly precipitation, monthly temperature, monthly mosquito species presence estimation (S1 Fig) are available as Supporting Information.

**Fig 6:**
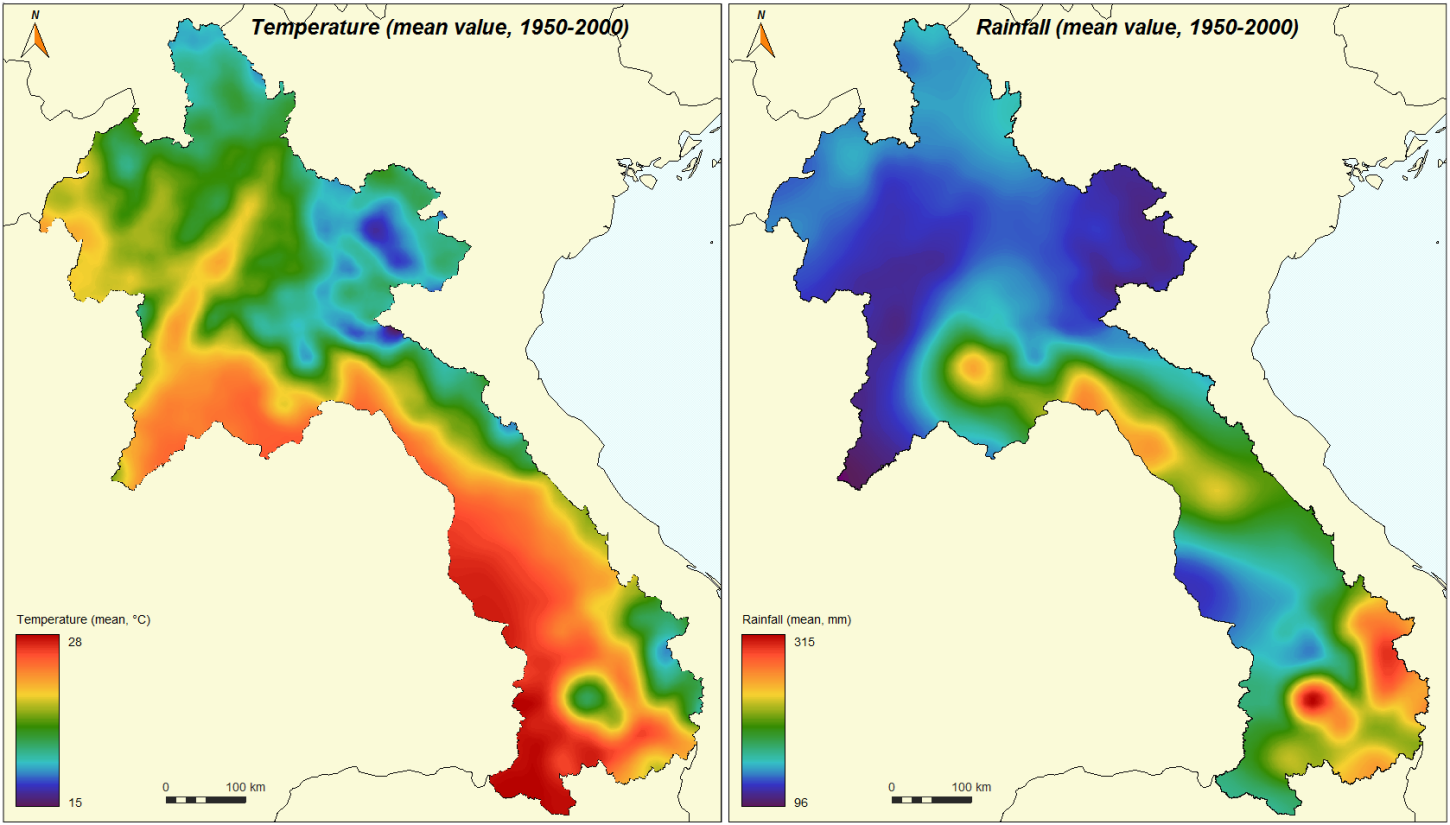
Annual mean temperatures (left panel) and annual mean rainfall (right panel) in Laos from 1950–2000 (from WorldClim—Global Climate Data).

Data collected on temperature (Fig 6, left panel) and precipitation (Fig 6, right panel) shows that the hottest and wettest areas are in southern Laos.

**Fig 7:**
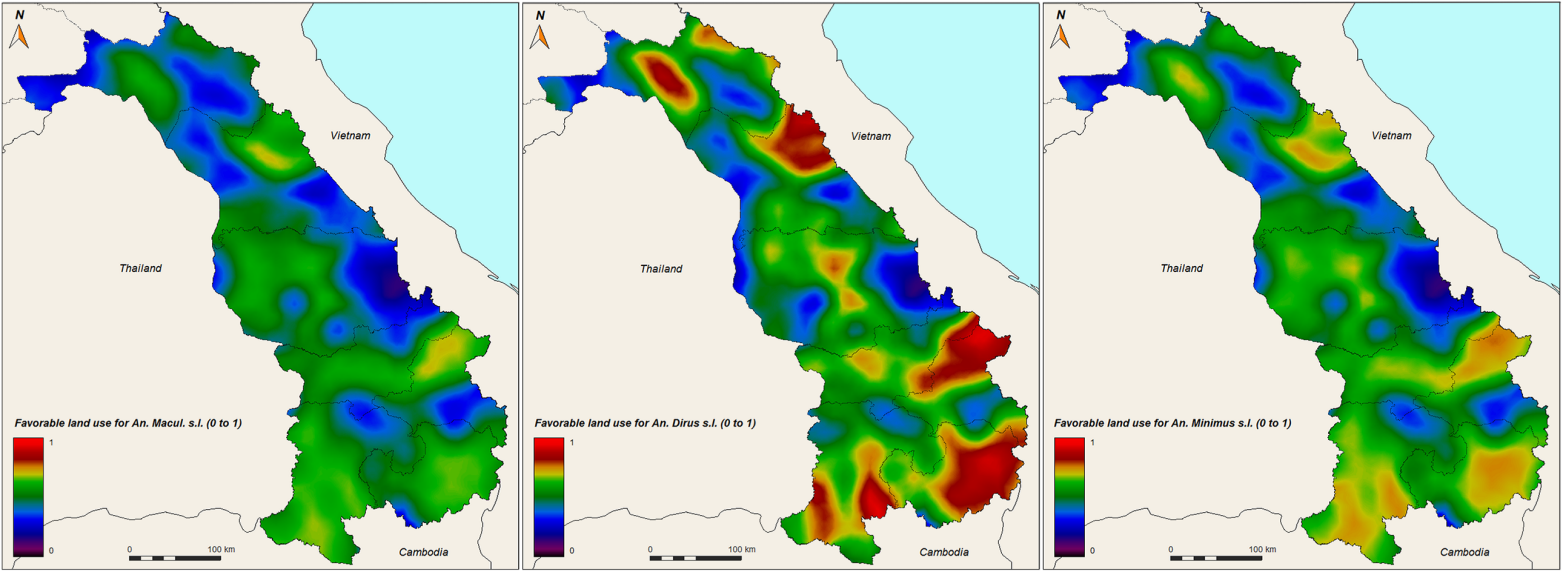
Probability of presence of *An*. *maculatus s*.*l*. (left panel), *An*. *dirus s*.*l*. (middle panel), and *An*. *minimus s*.*l*. (right panel) in central-southern Laos, based on land use favorable conditions only (Y3). Red color indicates higher probability.

The probability of vector presence based on land use only (Y3, Fig 7) shows similarities in spatial distribution. *Anopheles dirus* seems to have a stronger signal than the other species. The results show that probabilities of the presence of these species are highest in the southern part of the country.

**Fig 8:**
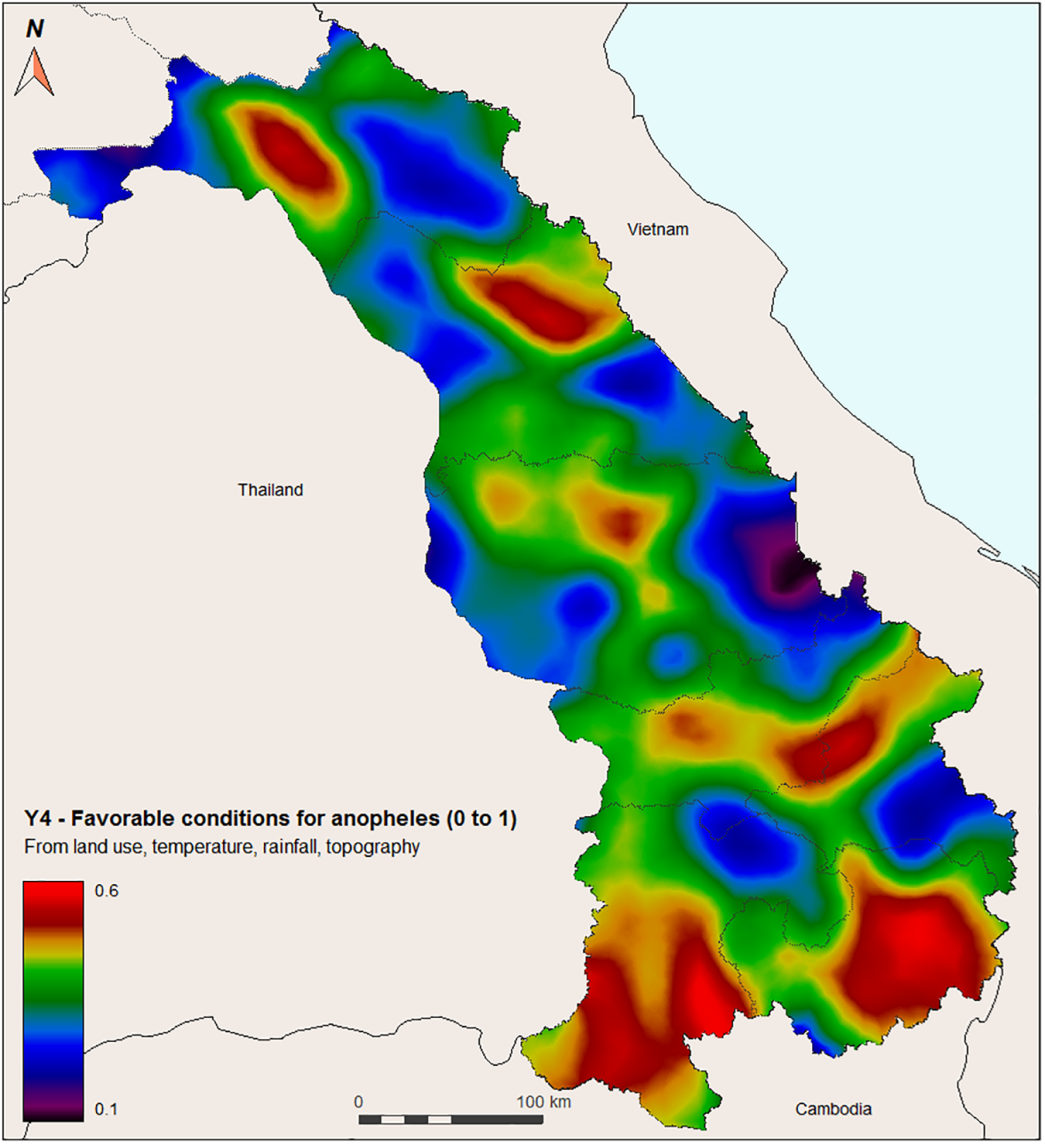
Probability of *Anopheles* mosquito presence (*An*. *maculatus s*.*l*., *An*. *dirus s*.*l*., and *An*. *minimus s*.*l*. combined) (Y4) based on land use (Y1), temperature (Y2), rainfall/topography (Y3), in central-southern Laos. Red color indicates higher probability.

When adding temperature, rainfall, and topography, the combined vector presence (all three species together) show slightly different patterns (Fig 8). The highest probabilities of vector presence are in Attapeu and Champasack provinces, as well as borders areas between Saravane and Sekong provinces. In addition, hilly and forested areas in the central provinces of Khammuane and Borikhamxay show higher probabilities of vector presence.

**Fig 9:**
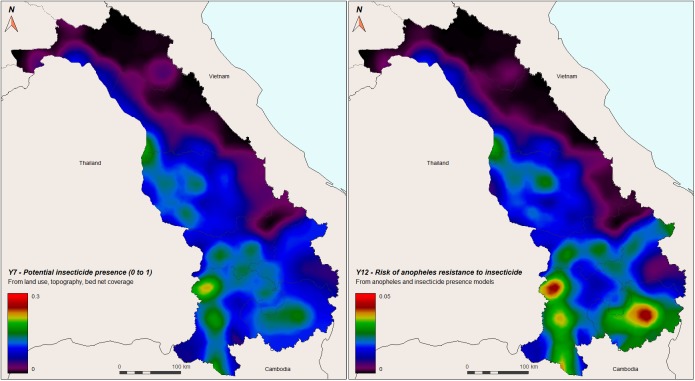
Left panel: Probability of insecticide presence (Y7), based on land use, topography, and bed net coverage, in central-southern Laos. Right panel: Risk of *Anopheles* mosquitoes to be exposed to insecticides and to become resistant (Y12) in central-southern Laos. Red color indicates higher risk.

The probability of insecticide use (Y7) was based on potentially insecticide-intensive land use and density of ITNs. The highest probabilities were found in the south of the country, specifically in several areas of Champasack, but also Savannakhet (Fig 9, left panel). The risk of malaria vectors becoming resistant to insecticides (Y12, Fig 9, right panel) also shows the highest probabilities in the south of the country, especially in the Champasack and Savannakhet provinces, near Thailand border.

**Fig 10:**
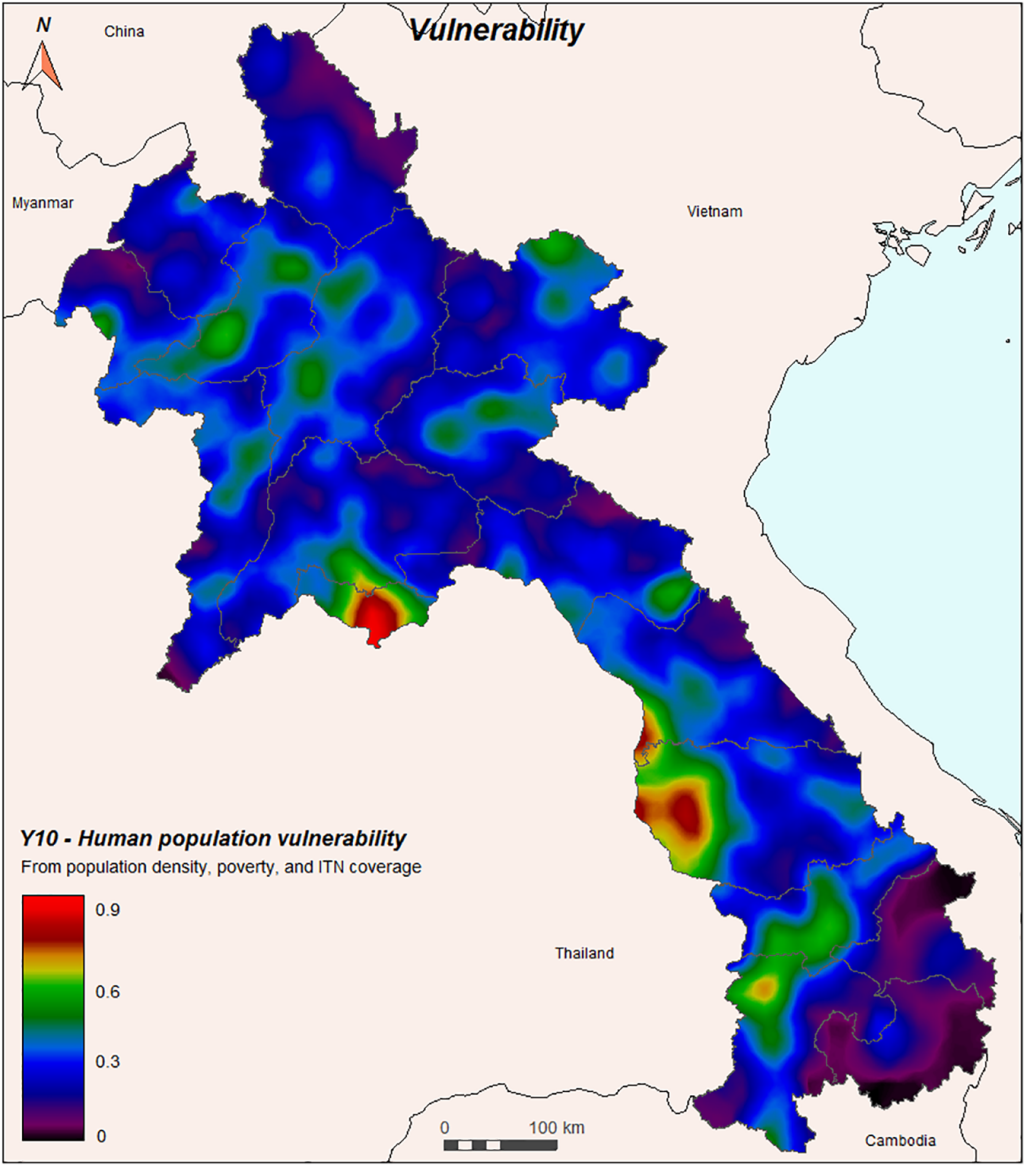
Vulnerability of the human population to *Anopheles* mosquitoes (Y10), combining human density and poverty (Y8) and number of ITNs per person (Y9).

The human population vulnerability was assessed by combining human density, human poverty, and the number of ITNs per person. The spatial distribution shows higher vulnerability in populated urban or semi-urban areas, especially near Vientiane and in the south near the border to Thailand (Pakse, Savannakhet, Takhet, Fig 10).

**Fig 11:**
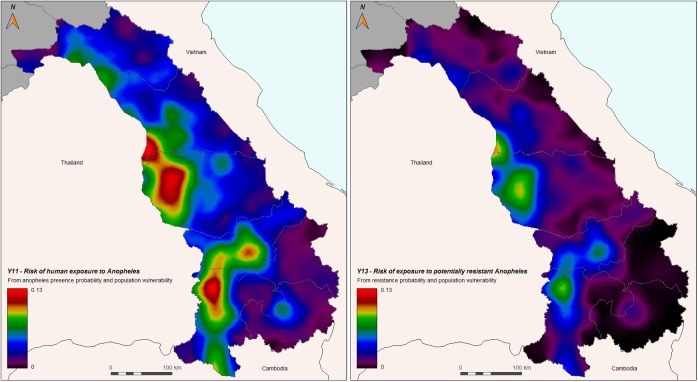
Left panel: Risk of vulnerable human hosts to be exposed to *Anopheles* mosquitoes (Y11), based on probability of vector presence (Y4) and population vulnerability (Y10). Right panel: Risk of vulnerable human hosts to be exposed to potentially insecticide resistant malaria vectors (Y13).

The risk of human exposure to *Anopheles* mosquitoes (Y11) and the risk of human exposure to *Anopheles* mosquito potentially resistant to insecticide (Y13) are shown in Fig 11. Overall, the risks are generally low (maximum is 0.13 for Y11, and 0.10 for Y13); the highest probabilities were found in the western areas of southern Laos, around populated centers such as Pakse, Savannakhet, and Takhek. Areas with especially low probabilities were found in the southeastern areas, e.g. in Attapeu to the border of Cambodia and Sekong to the border of Vietnam.

The maps show that most of the risk to be exposed to the main malaria vectors is concentrated in the southern part of Laos where malaria is the most active. We found that the correlation between *Anopheles* presence estimation and malaria incidence (2014), calculated by district over the central-southern part of the country is 0.51 (Bravais-Pearson’s correlation coefficient).

As shown in the maps of Fig 9, potential insecticide presence and therefore the probability of resistance to insecticide is also greater in the southern part of the country, especially where malaria incidence is the highest (Champasack province). Due to favorable climatic conditions and high potential for insecticide-intensive land-uses, more areas were identified in the southern part of the country than other regions to have a higher risk of resistance selection.

The locations with the highest risk of human exposure to malaria vectors are quite similar to locations with a high risk of human exposure to potentially resistant malaria vectors. In fact, both of these models have overlapping sub-models, the main difference being that the latter includes a major component of land use adjusted by an insecticide weighted factor and a minor component of number of ITNs per person (sub-model 2). The highest risks for insecticide resistance development in Laos are in southwestern Laos, specifically in rice and other agricultural cropping systems along the Mekong river and other agricultural areas in Champassack (Fig 9).

## 4. Discussion

Abundant literature has been generated describing and estimating the presence of *Anopheles* mosquitoes in South-East Asia (e.g. [25]). With more accurate data (especially on land-use) and temporal variations (for meteorological data), our model aims to improve the space-time evaluation and the geographical distribution of favorable conditions for *Anopheles* mosquito presence and potential insecticide resistance, with high spatial resolution.

Areas can be potentially favorable to mosquito presence but this is not sufficient to assume that the vector is actually present in the reality: mosquitoes need to be present in the region for the model to jump from potential to real. In Laos, *Anopheles* has been collected almost everywhere, and maps and models in previous studies show their presence everywhere in the country [25]. A recent study, conducted in 2013 up to 2015, showed that primary vectors can be found throughout Laos during both the dry and rainy seasons (Marcombe *et al*. “Insecticide resistance status of malaria vectors in Lao PDR”). Our model on favorable conditions for *Anopheles* can therefore be used as an indication of the abundance of vectors (in time and space) and not only as a probability of their presence.

Some densely-populated areas (especially urban centers) appear vulnerable whereas *Anopheles* mosquitoes have limited or no presence reported in these urban areas [25]. These results should not be surprising because vulnerability does not represent the risk, i.e. human populations may be vulnerable but not exposed to the threat, as is the case in urban areas where *Anopheles* have limited presence or may be located around the cities. It should also be noted that cells of 10x10 km may not represent an adequate aggregation scale to model the risk in urban areas because cells are too large: 10x10 km cells may aggregate inhabited urban areas with peri-urban and rural areas, where anopheline presence conditions can be met.

Our models inferring favorable conditions for mosquito presence or insecticide resistance are built from expert knowledge only: on the relationship between land-use, climate and mosquito favorable habitats, and between land-use and potential use of pesticides. We used information from bibliography and knowledge from scientists (entomologists and agronomists). We did not try to re-validate this knowledge by field surveys: the aim of this work was to transform the expert knowledge on *Anopheles*–environment relationships to spatial evaluation of the risk of *Anopheles* presence and abundance, insecticide presence and the risk of insecticide resistance in Laos.

The exposure of malaria vectors to insecticides is probably mainly driven by agricultural insecticides due to the intensity of agricultural pesticide input compared to the use of public health insecticides. Although, there are few exact figures on pesticide input in Laos. National registration system for insecticides is lacking in Laos and should be further developed to ensure sounds management of insecticides. Models would be also significantly improved if reliable data would be available on pesticide use and types of insecticides used in public health and crop protection. A limitation of this study was that we do not know exactly which insecticides the mosquitoes are exposed to. This will affect the likelihood of resistance development and cross-resistance patterns. It is extremely difficult to assess actual pesticide use in the agricultural sector. This is because of specific preferences and practices of individual farmers, type of crops grown which often vary from year to year, and large spatio-temporal variations in presence of crop pests. Due to these uncertainties, it is generally not possible to specify the classes of insecticides used, but rather the intensity of use based on prior knowledge of which land uses are more vulnerable and require protection through pesticide input. In a country like Laos there is very little data available on pesticide use, therefore the model is based on data from Thailand [12].

Our models take into account only environmental variables; in fact, mosquito presence and abundance can be related to many other variables, especially anthropogenic variables, so here “risk” needs to be understood as “environmental risk”. The model gives only a part of the actual risk. In this context, the correlation between malaria cases and favorable conditions for the vector presence (Bravais-Pearson's coef. = 0.48) is high, because only environmental factors related to the presence of the mosquito are taken into account, excluding parameters regarding the presence of the parasite or anthropogenic parameters that may influence the spread of malaria.

As mentioned in the introduction, these models for mosquito abundance and resistance are difficult to validate on the field. Their main interest consists, on the one hand, in directing the entomological surveys towards zones and times when the probability of presence and abundance of the *Anopheles* is the highest and on the other hand to direct preventive vector control, public health actions and surveillance systems to these zones. Contrasting with entomological collection and evaluation on the field, these methods are rapid, at low cost, easy to implement, and results may cover large territories with high spatial resolution.

## 5. Conclusion

The mapping and statistical results of this work highlighted the space-time distribution of the environmental risk of *Anopheles* presence, potential insecticide emergence, insecticide resistance, and risk of exposure to these threats for the human population in Laos. These results are based on expert knowledge, and on practices observed in Thailand concerning agricultural policies and insecticide use. We found several areas of higher risk for the development of resistance, especially in agricultural areas located in the southern part of the country close to the Thailand border.

The results of this work can be used to help develop public health policies and to implement targeted vector control strategies for malaria prevention and elimination. Furthermore, they may be used to focus the search for insecticide resistance among mosquito populations in Laos, reducing considerably the area of the surveys, and making possible the implementation of a surveillance system for *Anopheles* resistance to insecticide.

## Supporting information

S1 Table Table giving risks results by district and province for southern Laos. (XLSX)

S1 Fig Maps of meteorological data for Laos, by month (temperature, rainfall) and maps of anopheles presence probability, by month and specie. (ZIP)

S1 Macro SavGIS macro-command grouping all commands used in the GIS modelling process. (MAC)
