# Supplementary figures and images for: Modeling spatial variation in risk of presence and insecticide resistance for malaria vectors in Laos
Source: PLoS One. 2017 May 11;12(5):e0177274. doi: 10.1371/journal.pone.0177274 (PMC5426714; doi:10.1371/journal.pone.0177274)

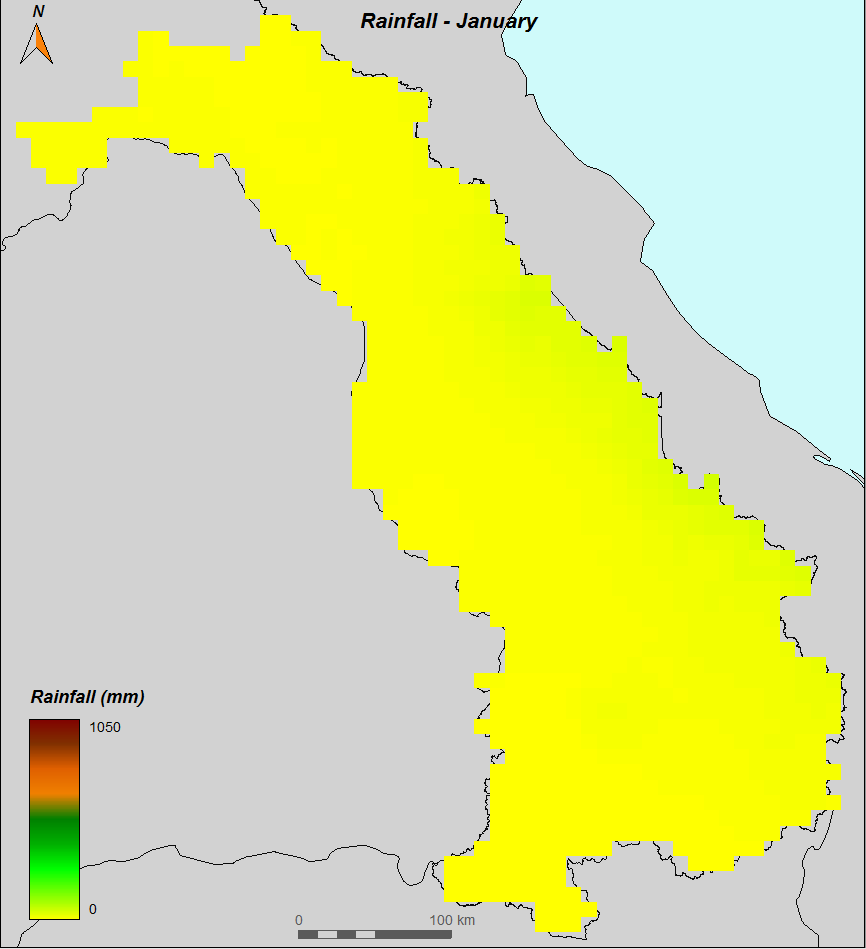

Supplement: S1 Fig — (ZIP) [file pone.0177274.s002.zip › RF_01.tif]

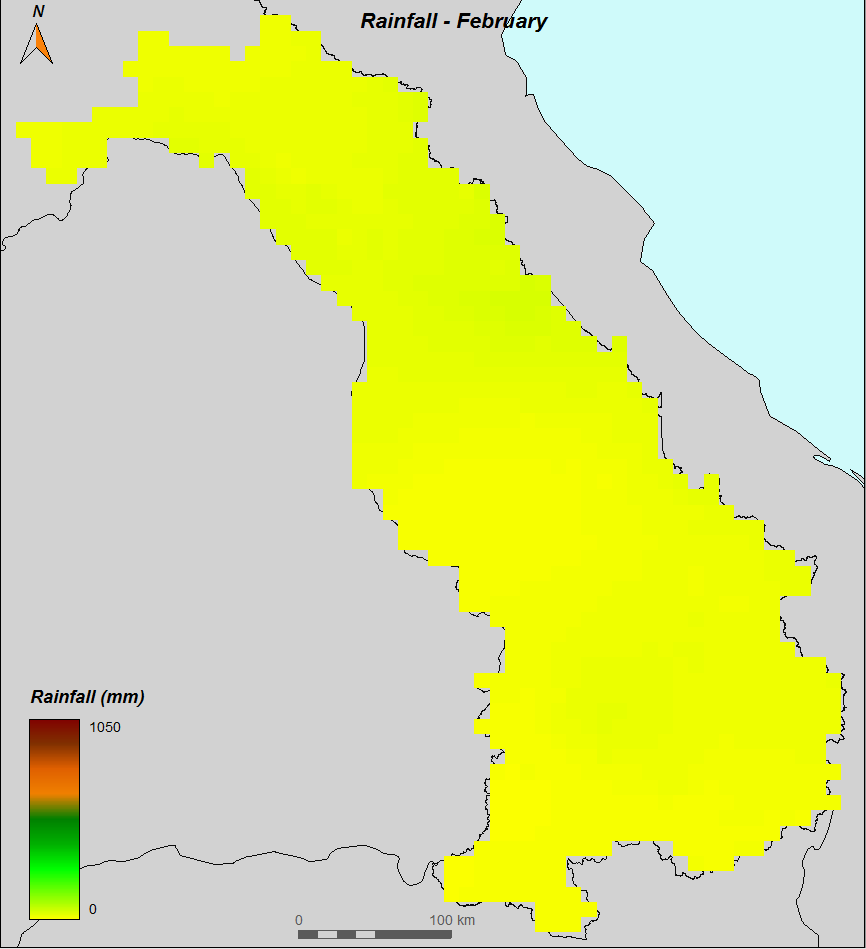

Supplement: S1 Fig — (ZIP) [file pone.0177274.s002.zip › RF_02.tif]

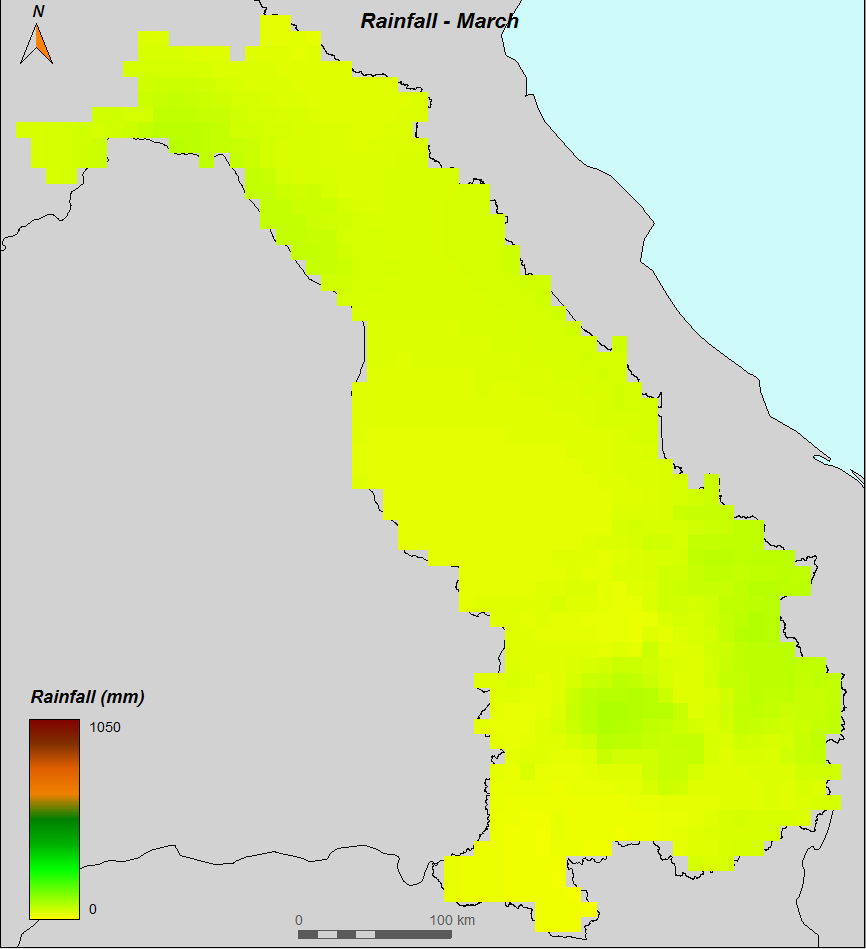

Supplement: S1 Fig — (ZIP) [file pone.0177274.s002.zip › RF_03.tif]

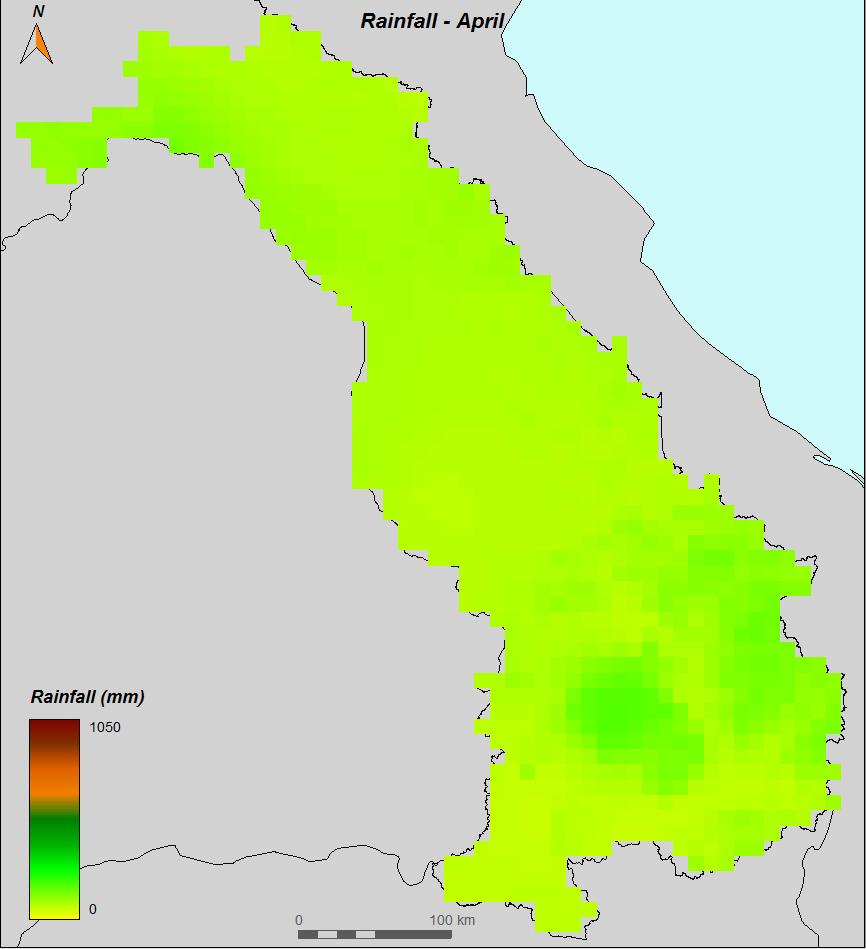

Supplement: S1 Fig — (ZIP) [file pone.0177274.s002.zip › RF_04.tif]

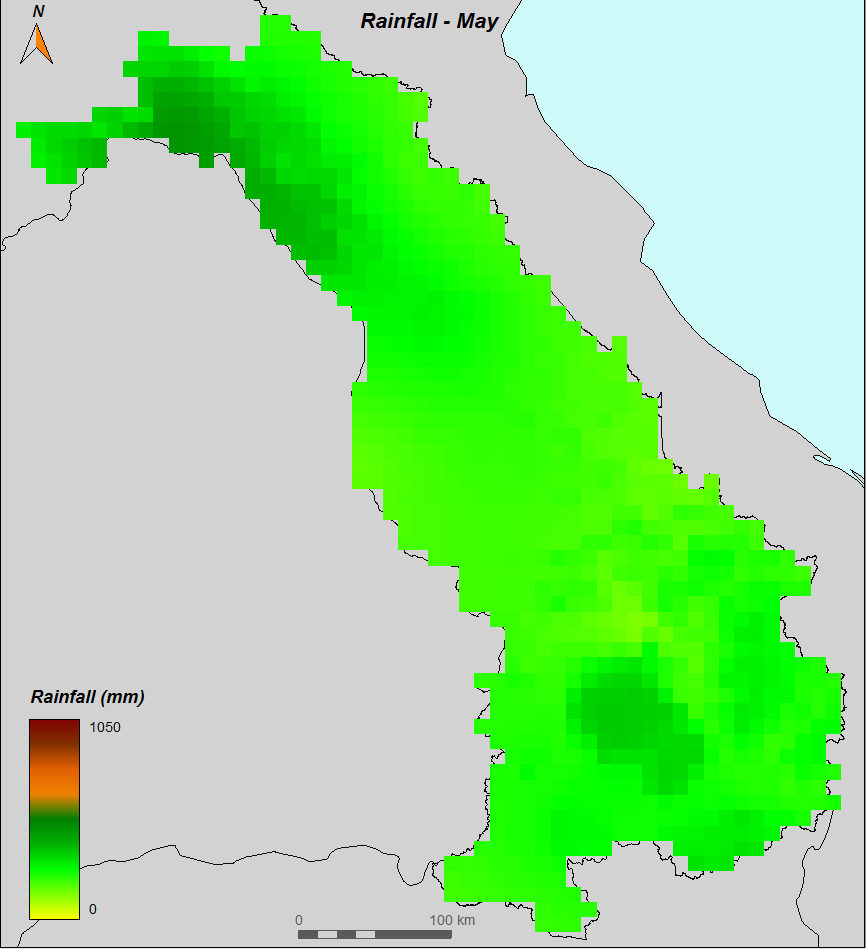

Supplement: S1 Fig — (ZIP) [file pone.0177274.s002.zip › RF_05.tif]

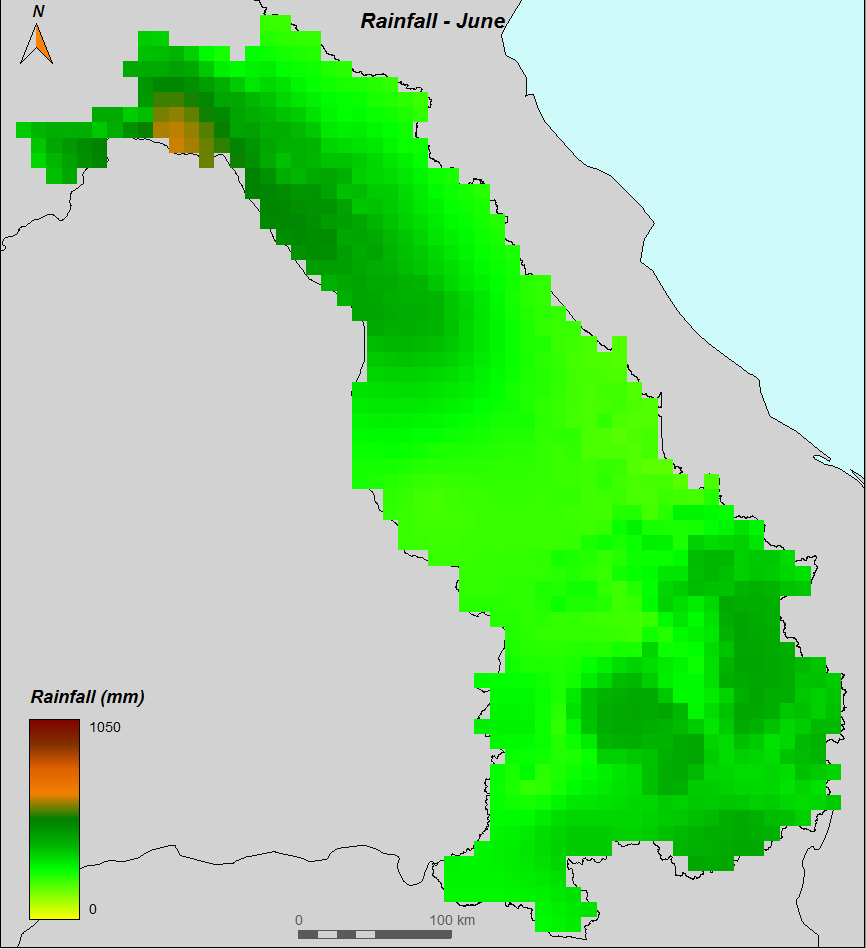

Supplement: S1 Fig — (ZIP) [file pone.0177274.s002.zip › RF_06.tif]

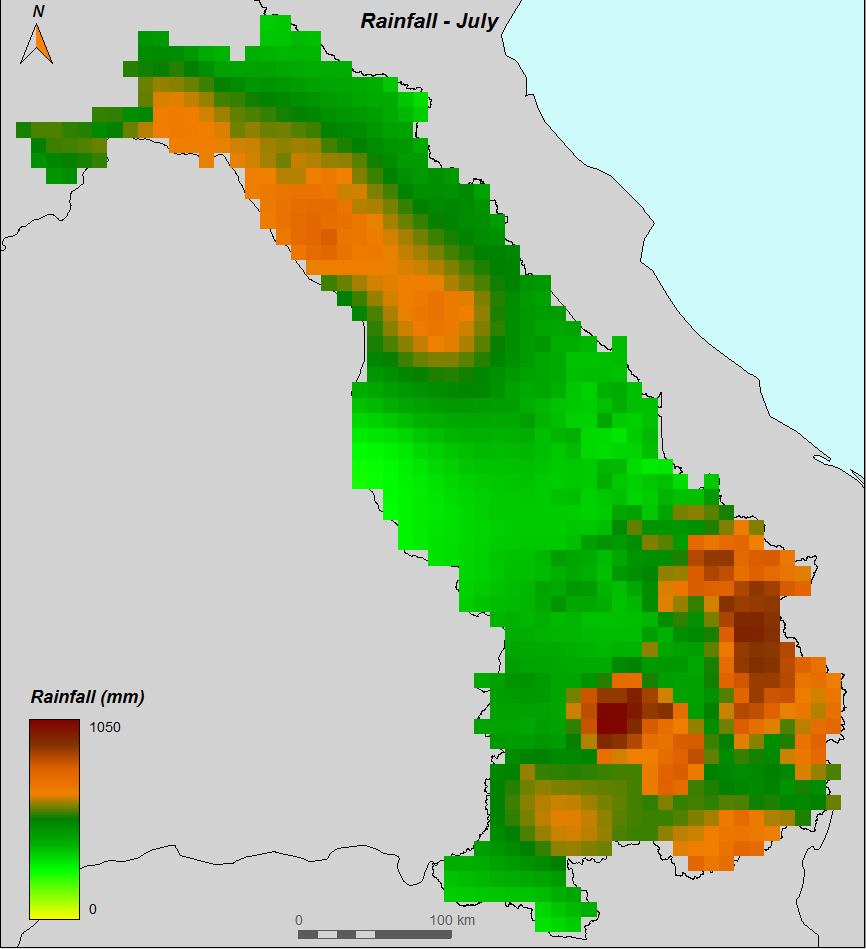

Supplement: S1 Fig — (ZIP) [file pone.0177274.s002.zip › RF_07.tif]

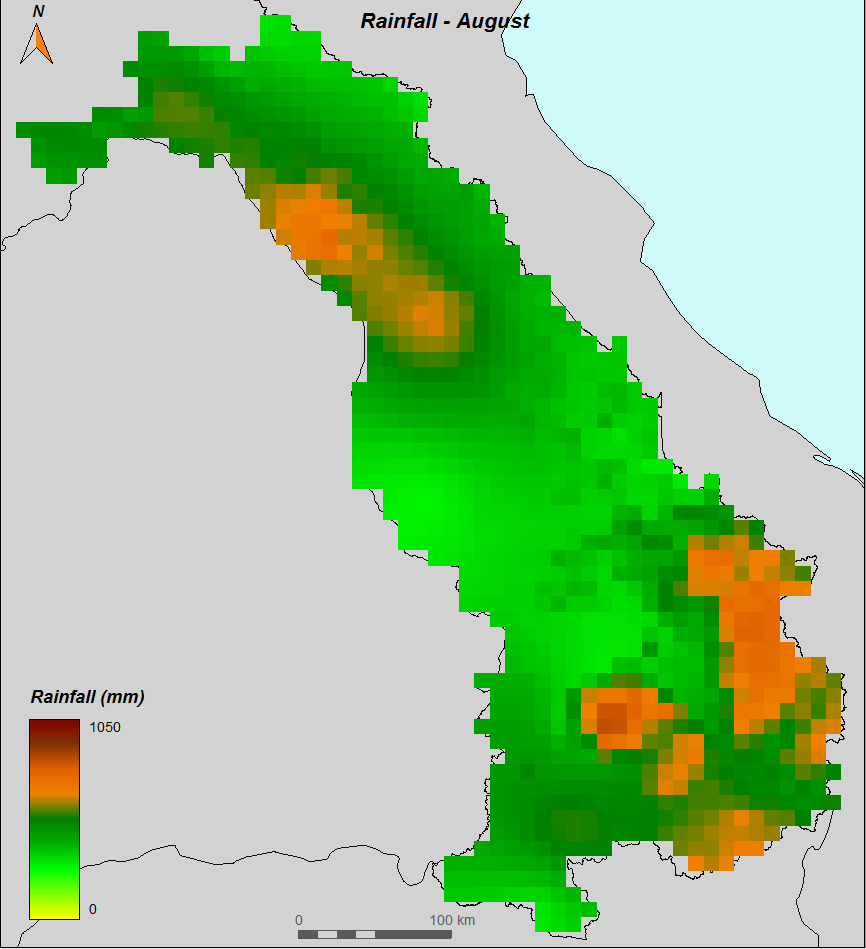

Supplement: S1 Fig — (ZIP) [file pone.0177274.s002.zip › RF_08.tif]

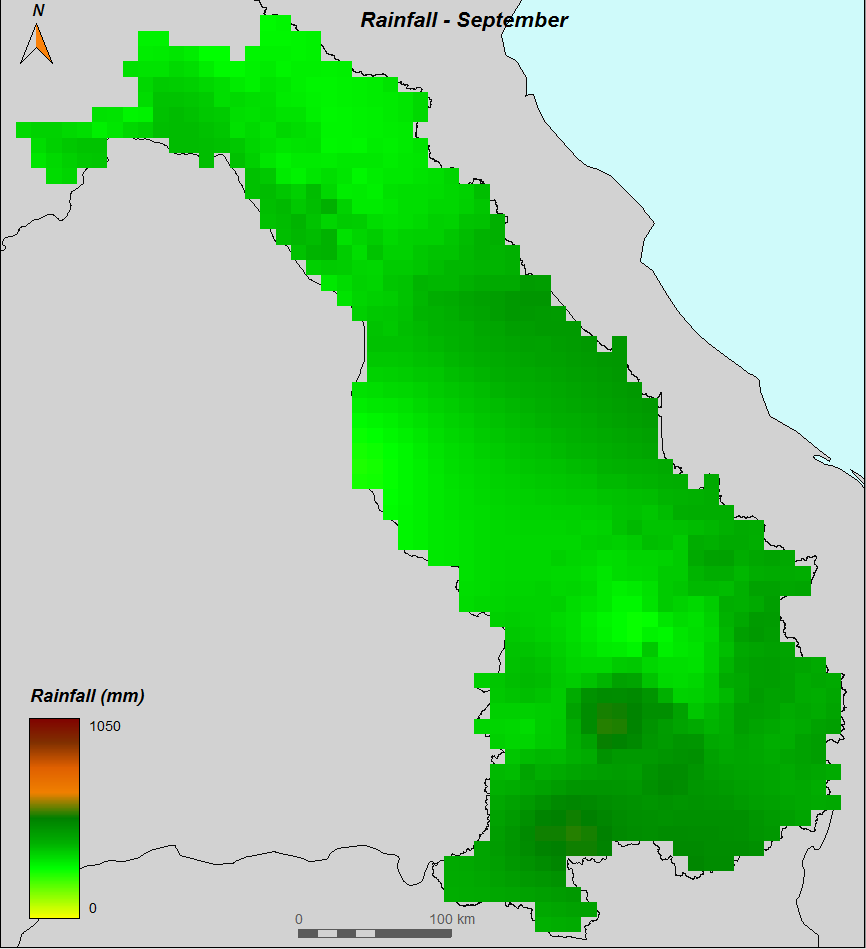

Supplement: S1 Fig — (ZIP) [file pone.0177274.s002.zip › RF_09.tif]

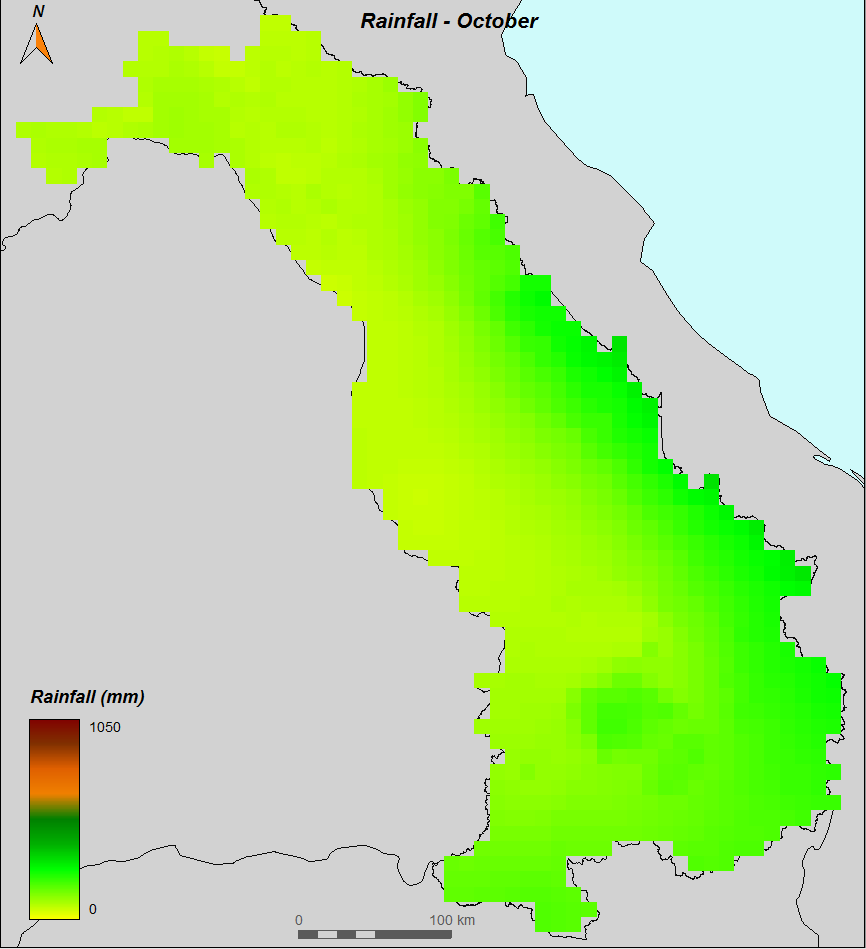

Supplement: S1 Fig — (ZIP) [file pone.0177274.s002.zip › RF_10.tif]

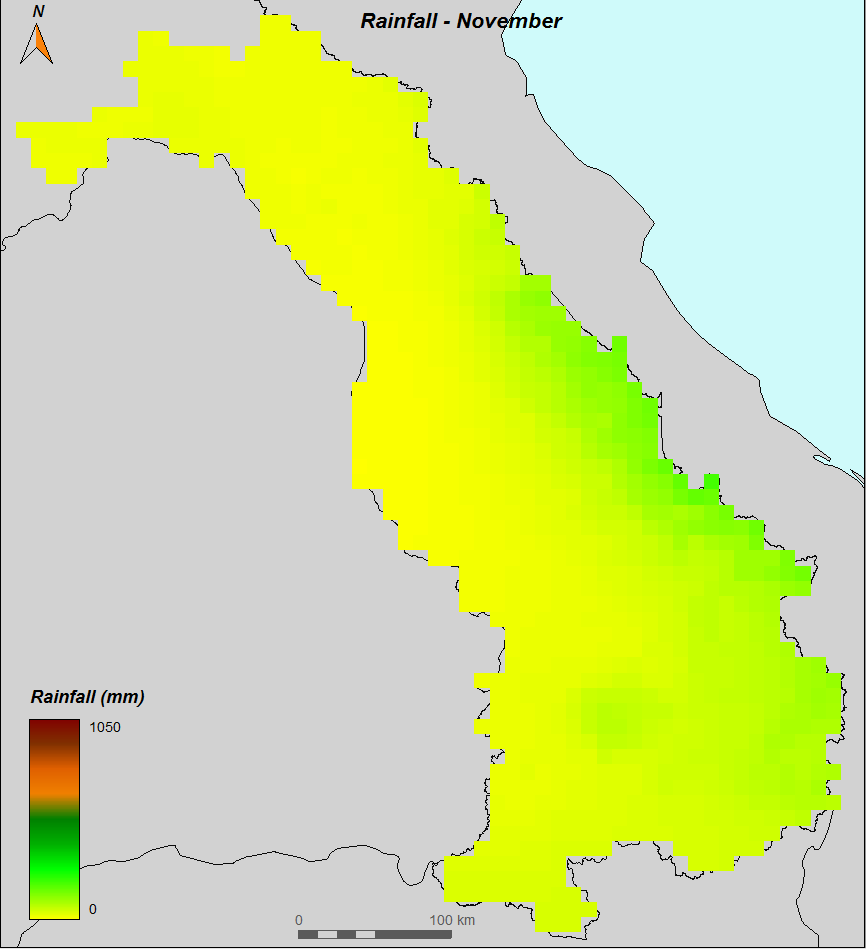

Supplement: S1 Fig — (ZIP) [file pone.0177274.s002.zip › RF_11.tif]

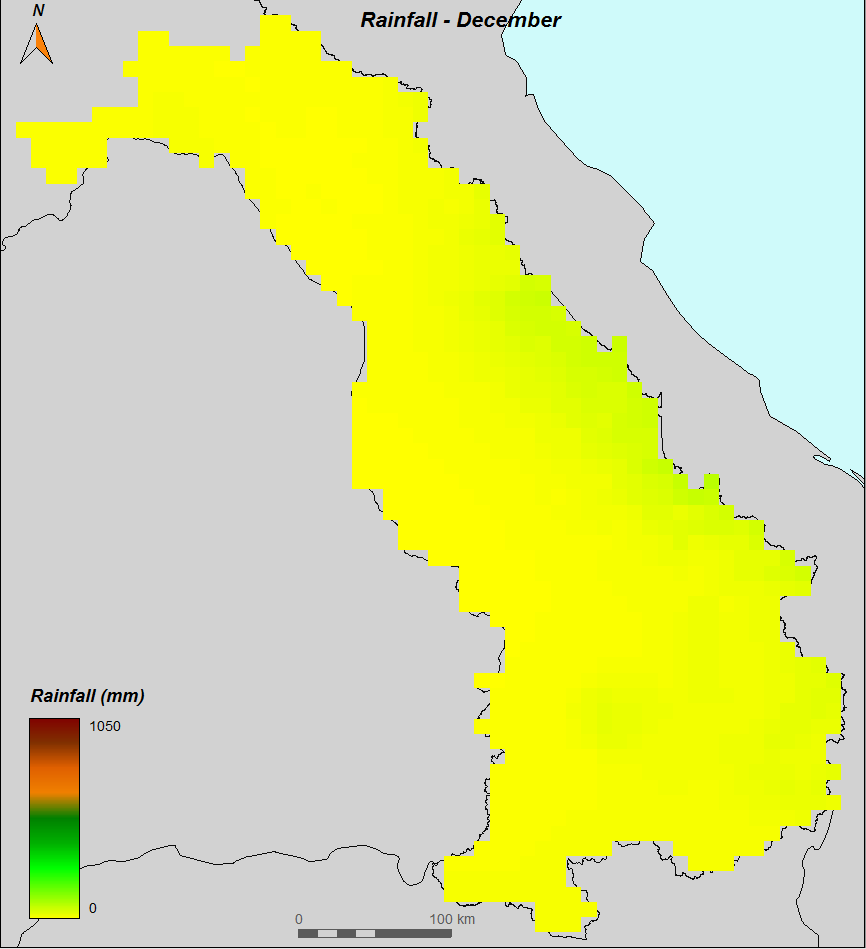

Supplement: S1 Fig — (ZIP) [file pone.0177274.s002.zip › RF_12.tif]

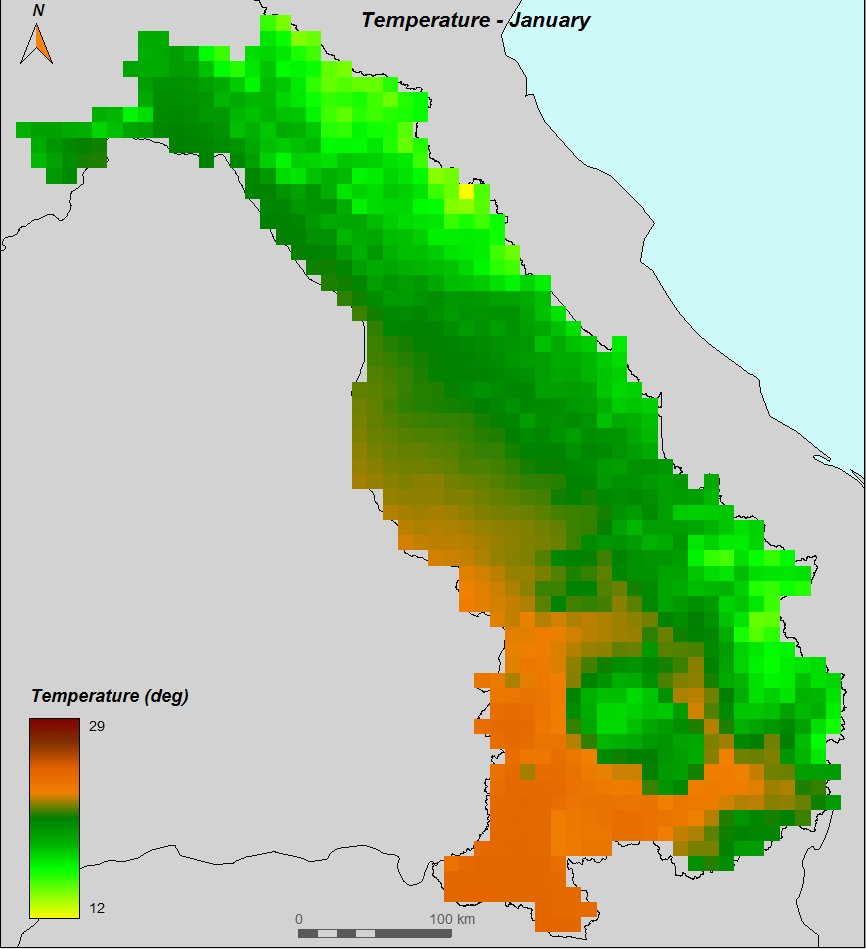

Supplement: S1 Fig — (ZIP) [file pone.0177274.s002.zip › T_01.tif]

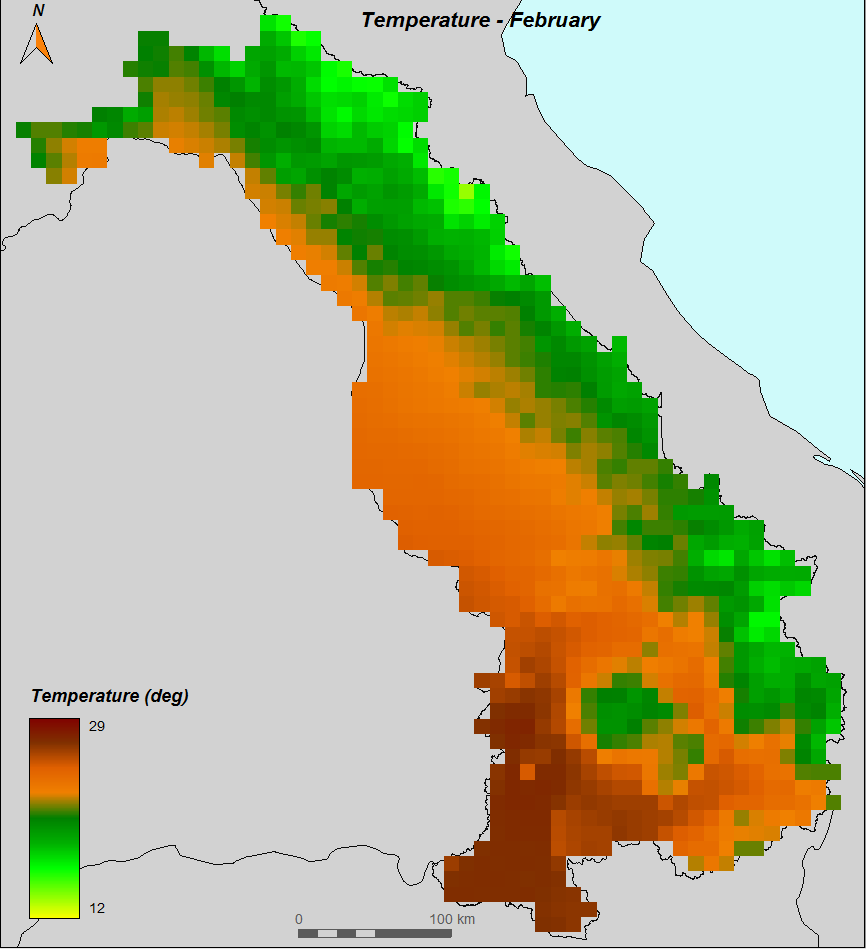

Supplement: S1 Fig — (ZIP) [file pone.0177274.s002.zip › T_02.tif]

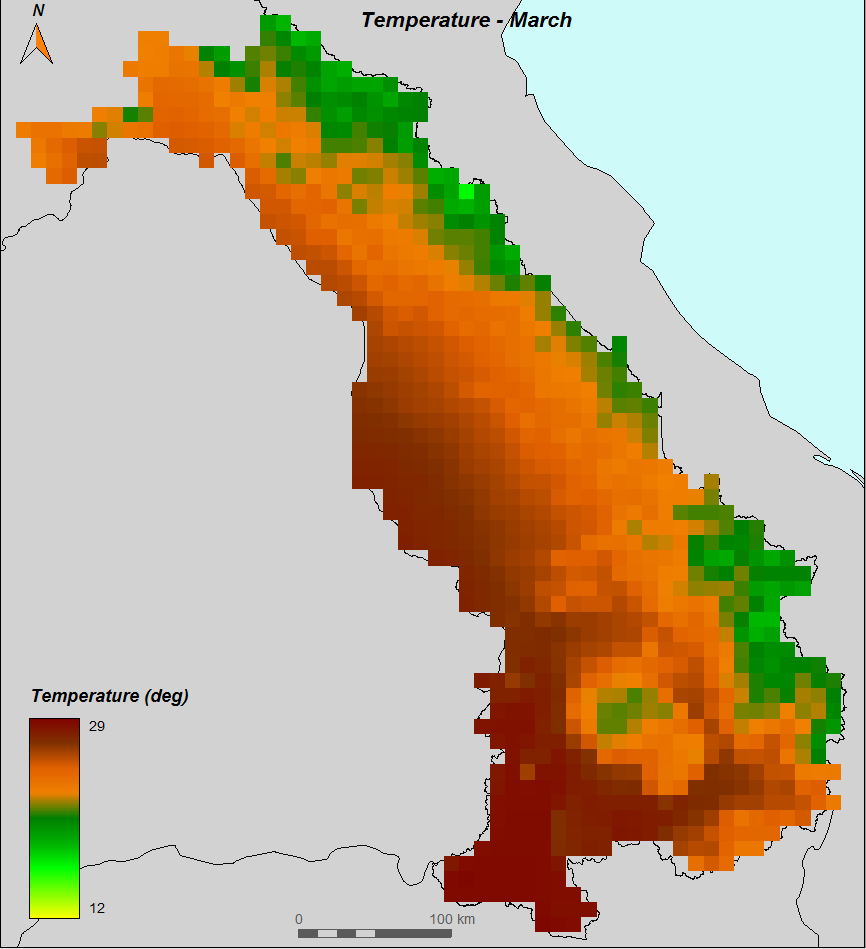

Supplement: S1 Fig — (ZIP) [file pone.0177274.s002.zip › T_03.tif]

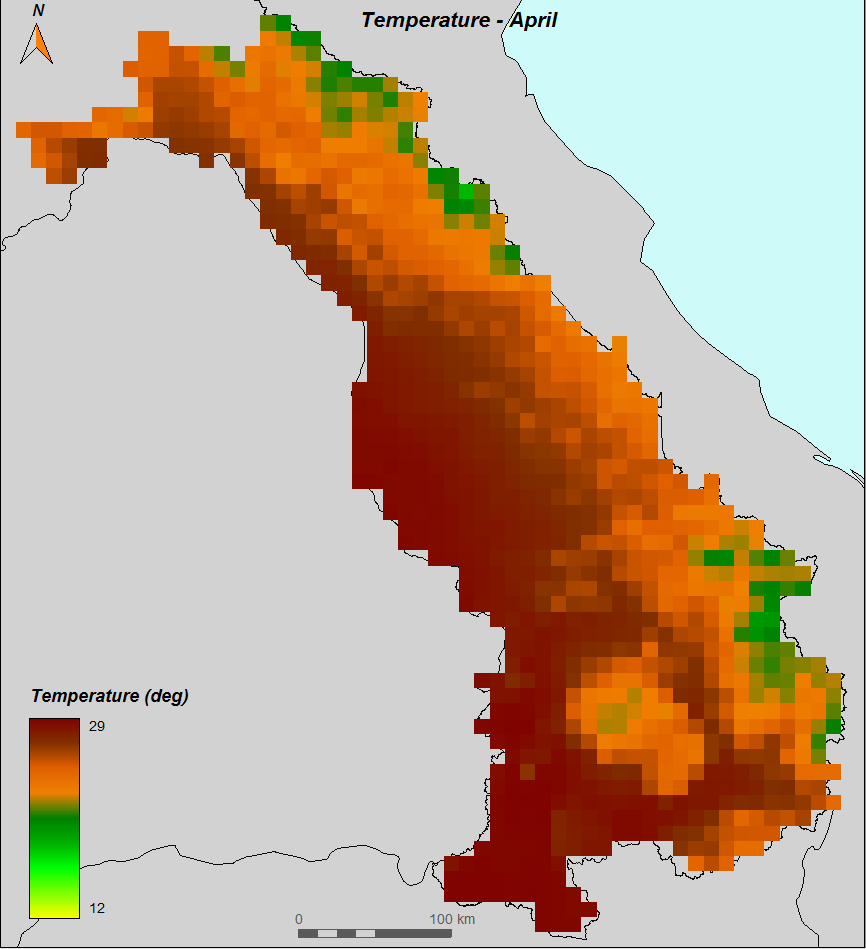

Supplement: S1 Fig — (ZIP) [file pone.0177274.s002.zip › T_04.tif]

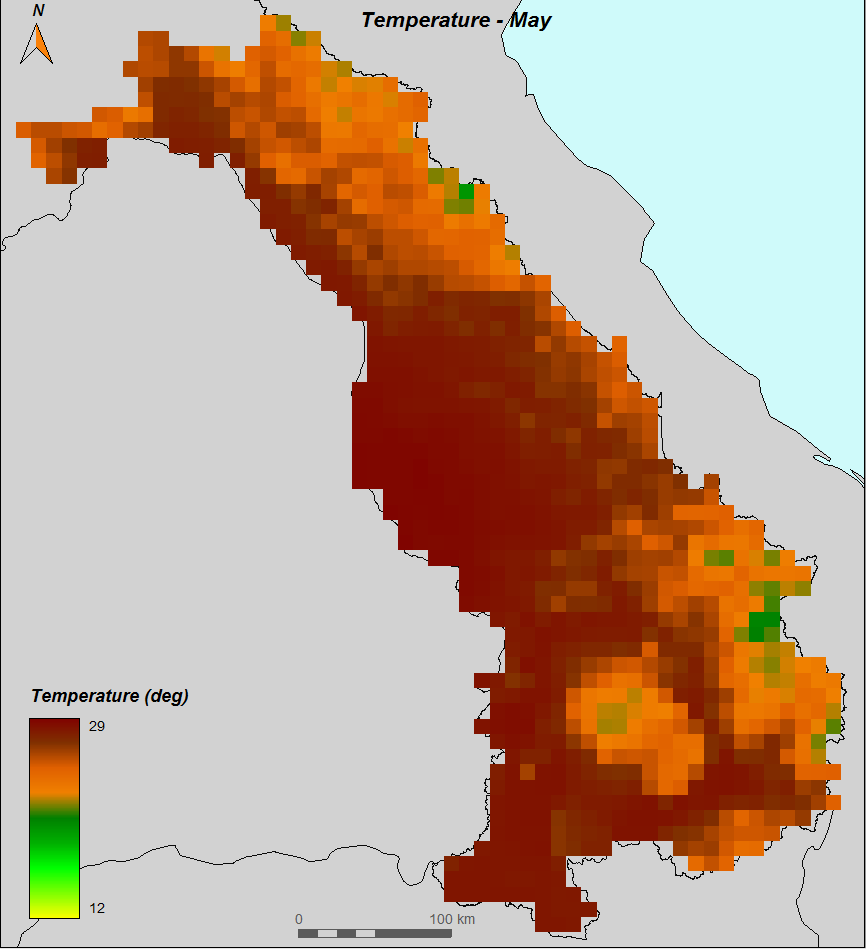

Supplement: S1 Fig — (ZIP) [file pone.0177274.s002.zip › T_05.tif]

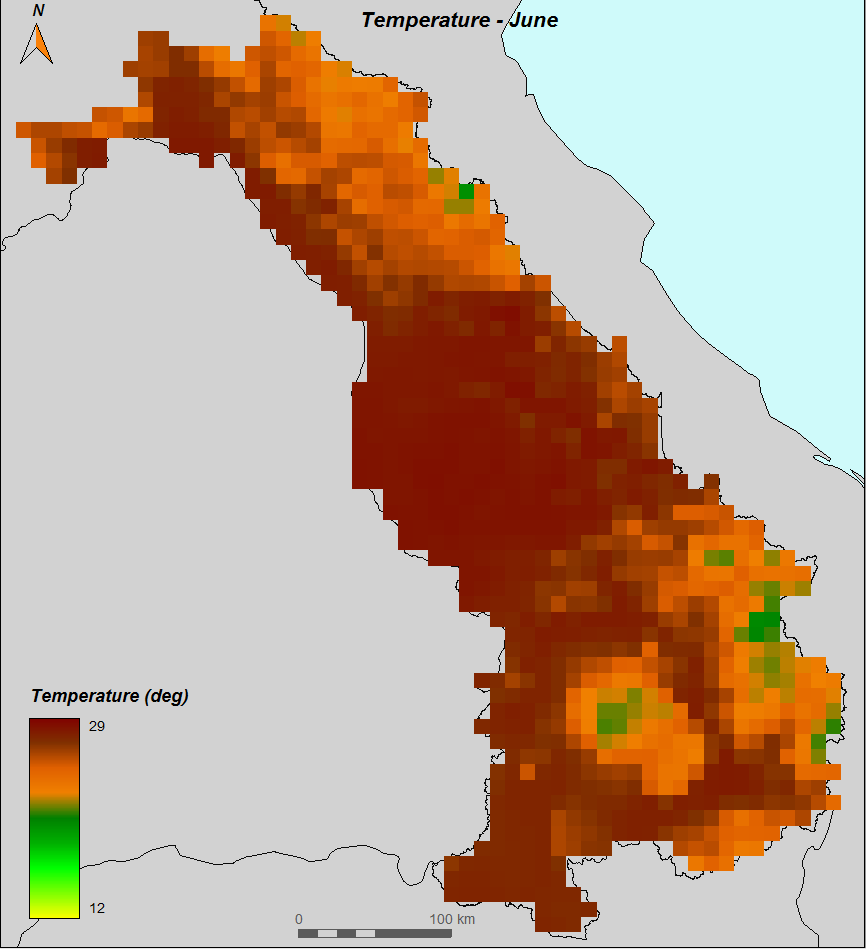

Supplement: S1 Fig — (ZIP) [file pone.0177274.s002.zip › T_06.tif]

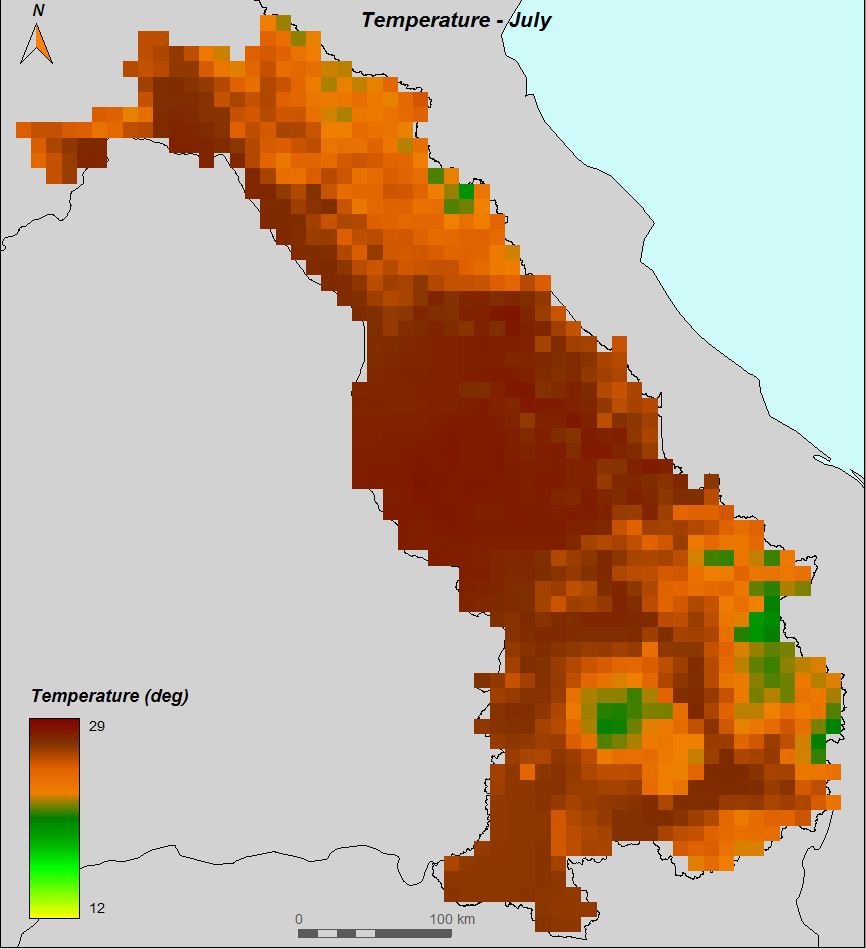

Supplement: S1 Fig — (ZIP) [file pone.0177274.s002.zip › T_07.tif]

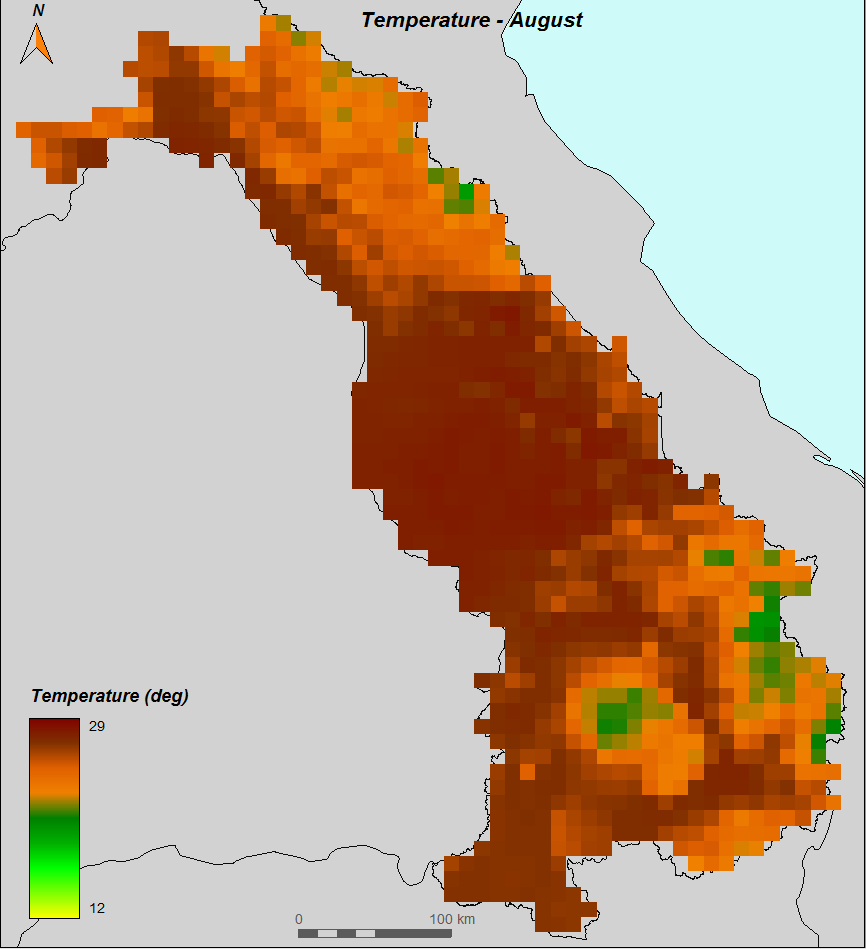

Supplement: S1 Fig — (ZIP) [file pone.0177274.s002.zip › T_08.tif]

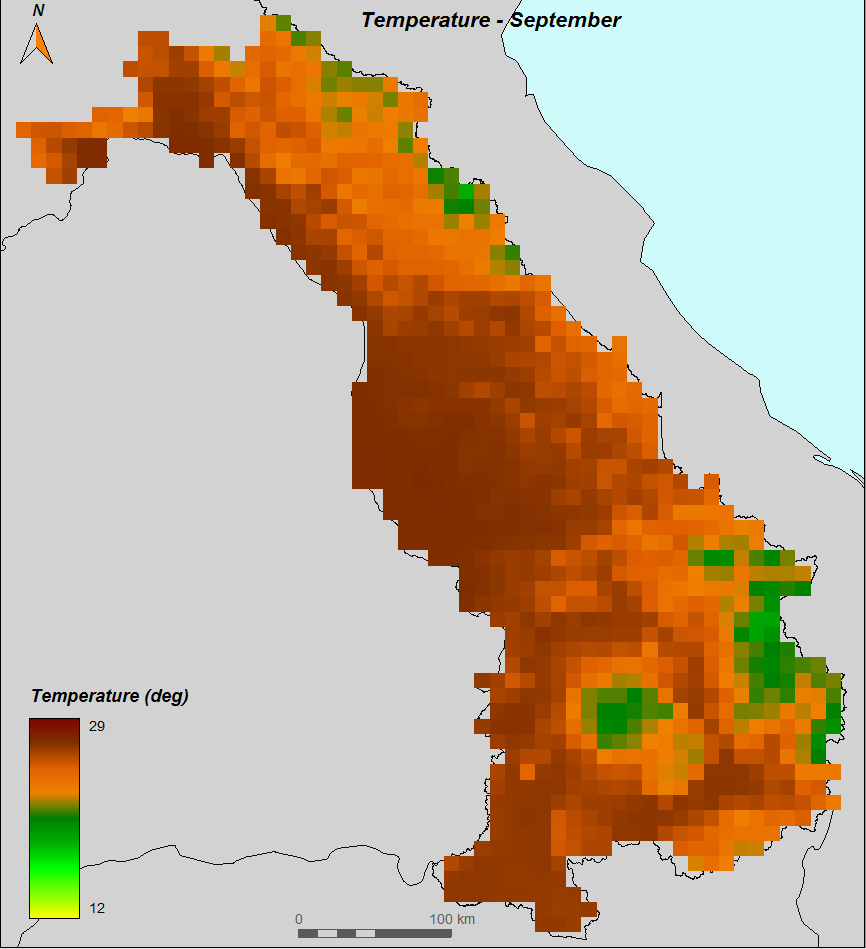

Supplement: S1 Fig — (ZIP) [file pone.0177274.s002.zip › T_09.tif]

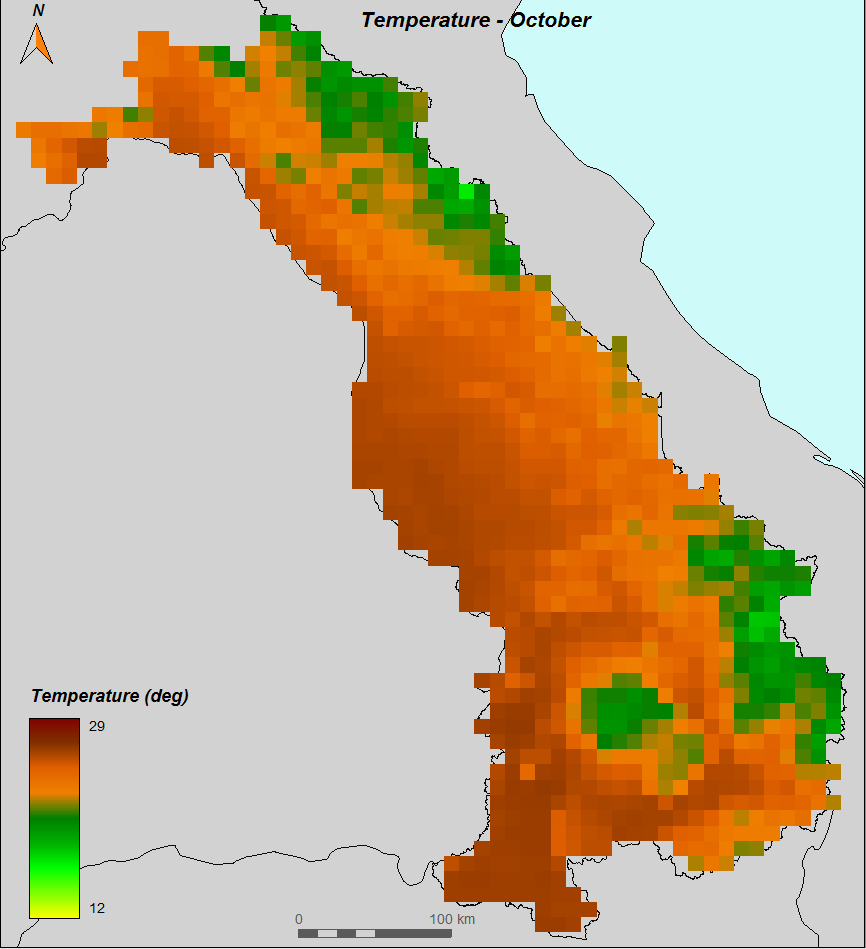

Supplement: S1 Fig — (ZIP) [file pone.0177274.s002.zip › T_10.tif]

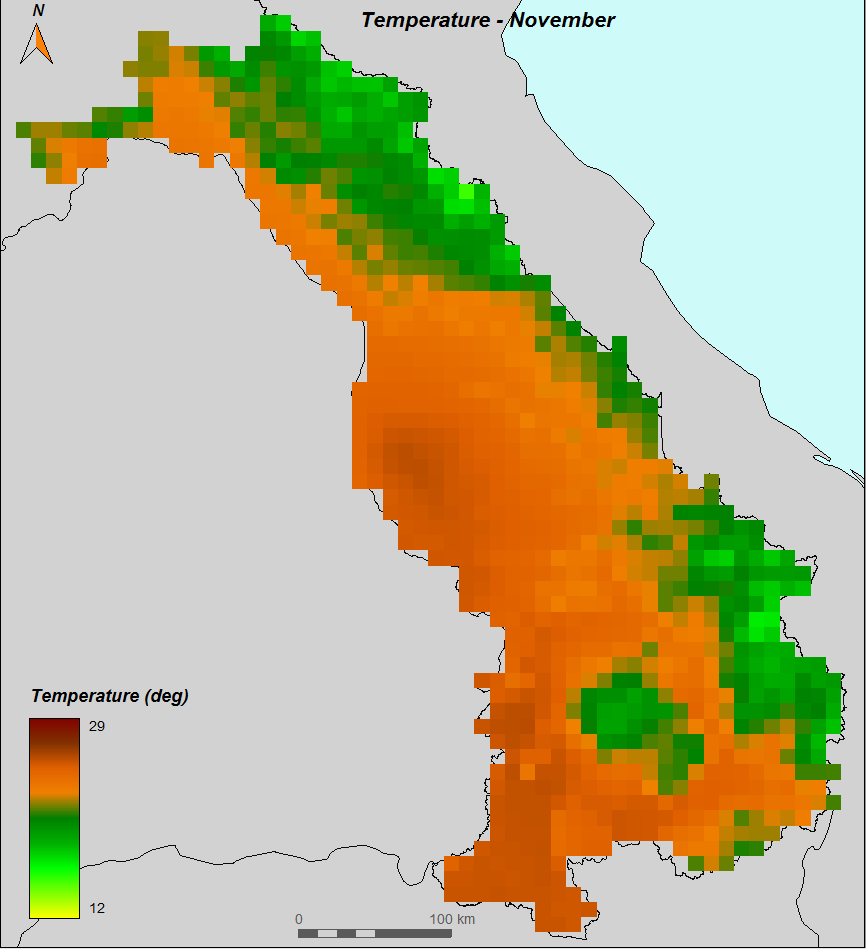

Supplement: S1 Fig — (ZIP) [file pone.0177274.s002.zip › T_11.tif]

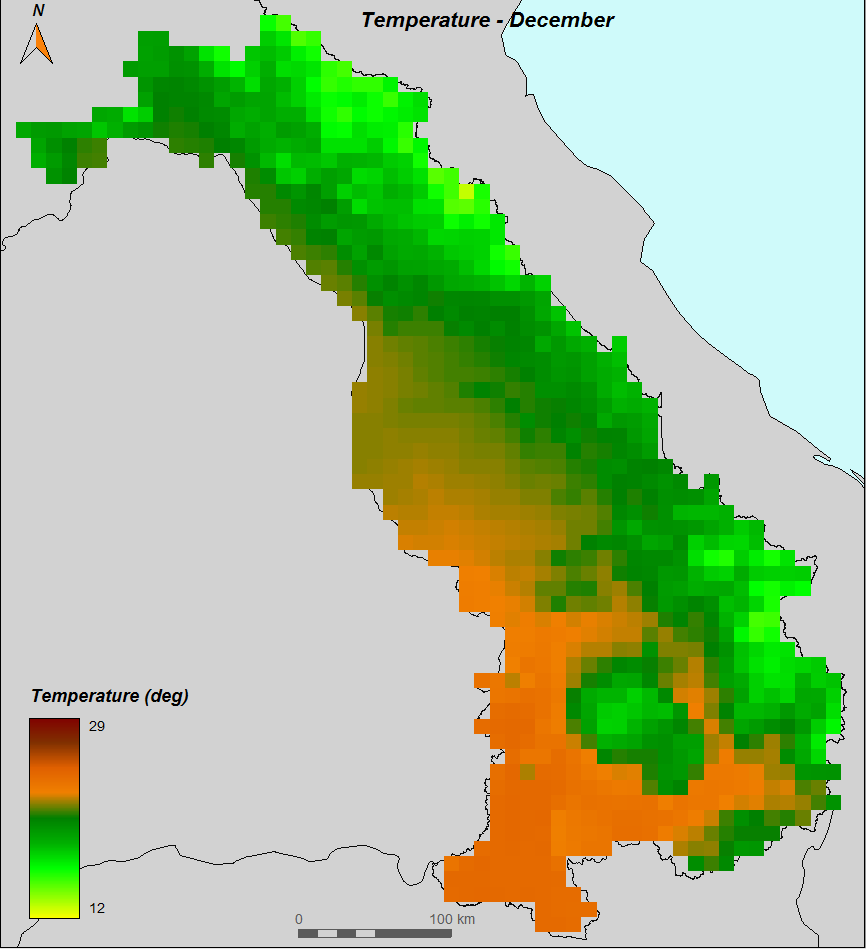

Supplement: S1 Fig — (ZIP) [file pone.0177274.s002.zip › T_12.tif]

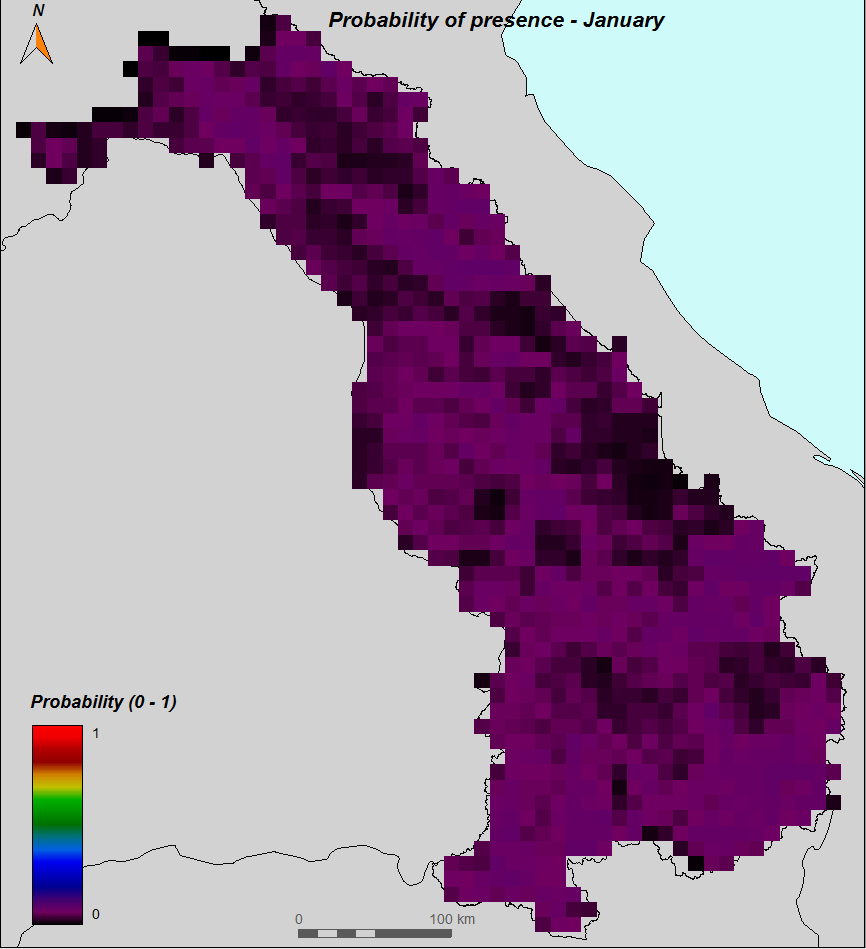

Supplement: S1 Fig — (ZIP) [file pone.0177274.s002.zip › Y4_01.tif]

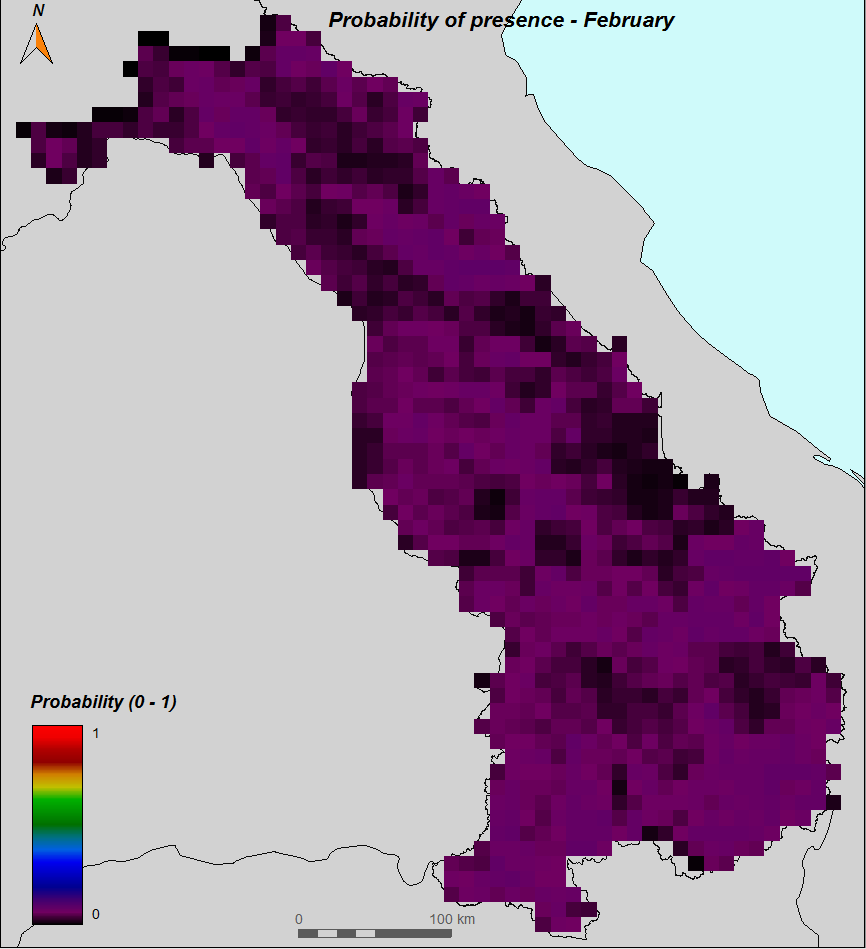

Supplement: S1 Fig — (ZIP) [file pone.0177274.s002.zip › Y4_02.tif]

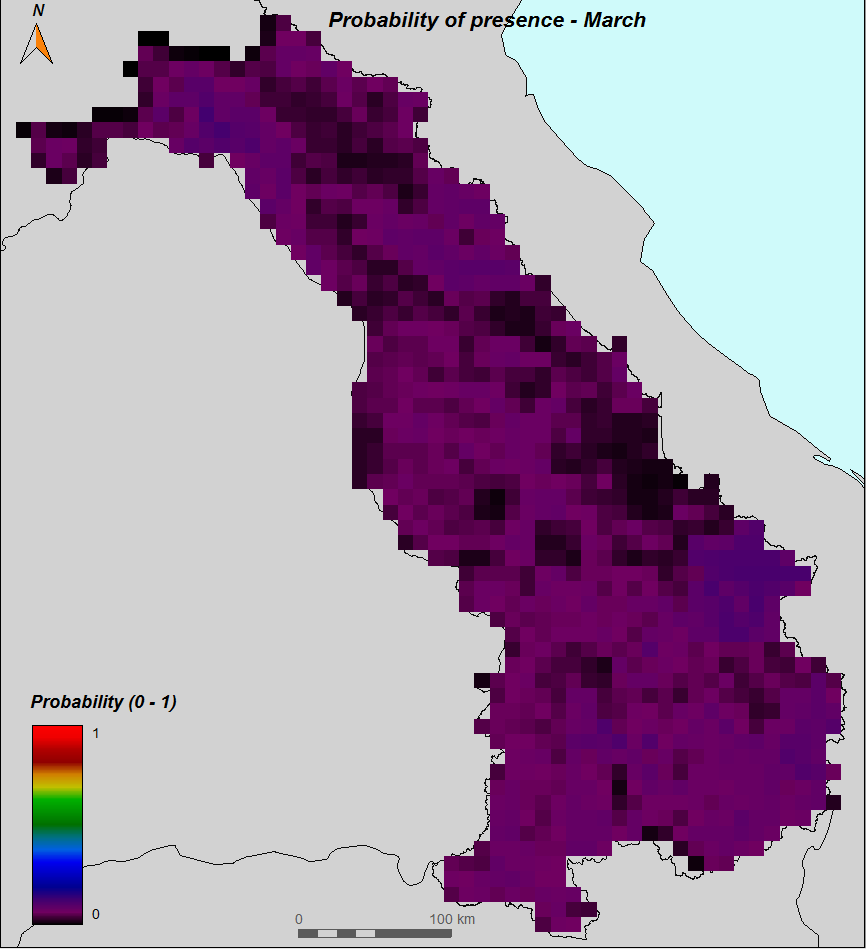

Supplement: S1 Fig — (ZIP) [file pone.0177274.s002.zip › Y4_03.tif]

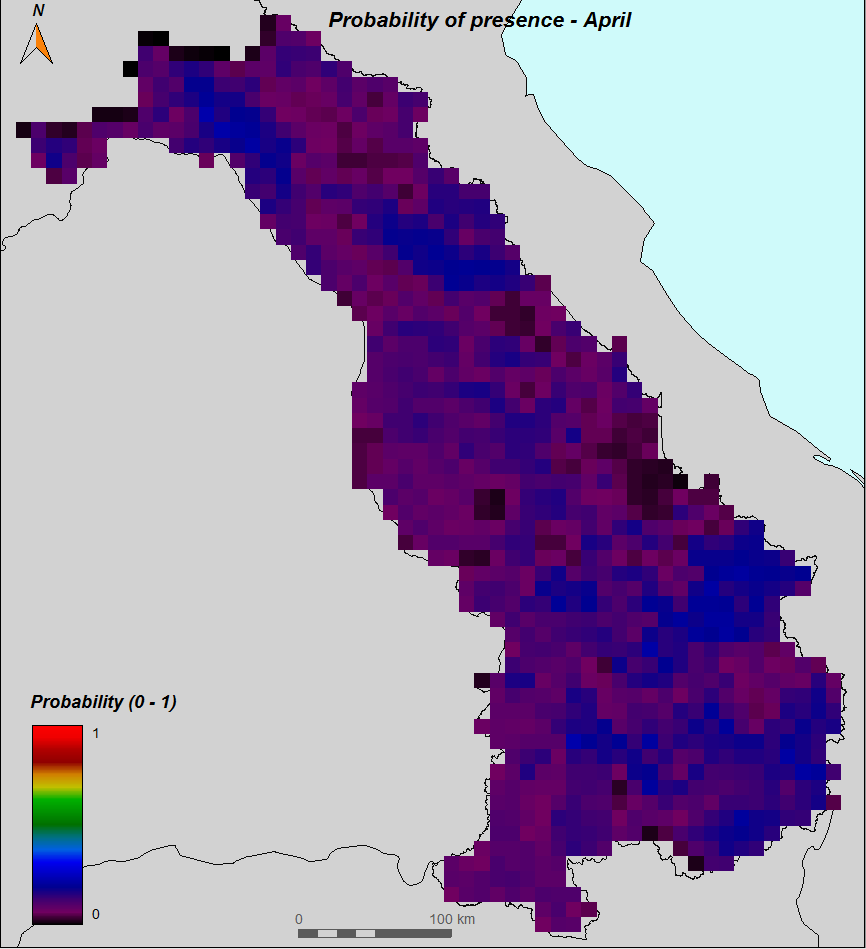

Supplement: S1 Fig — (ZIP) [file pone.0177274.s002.zip › Y4_04.tif]

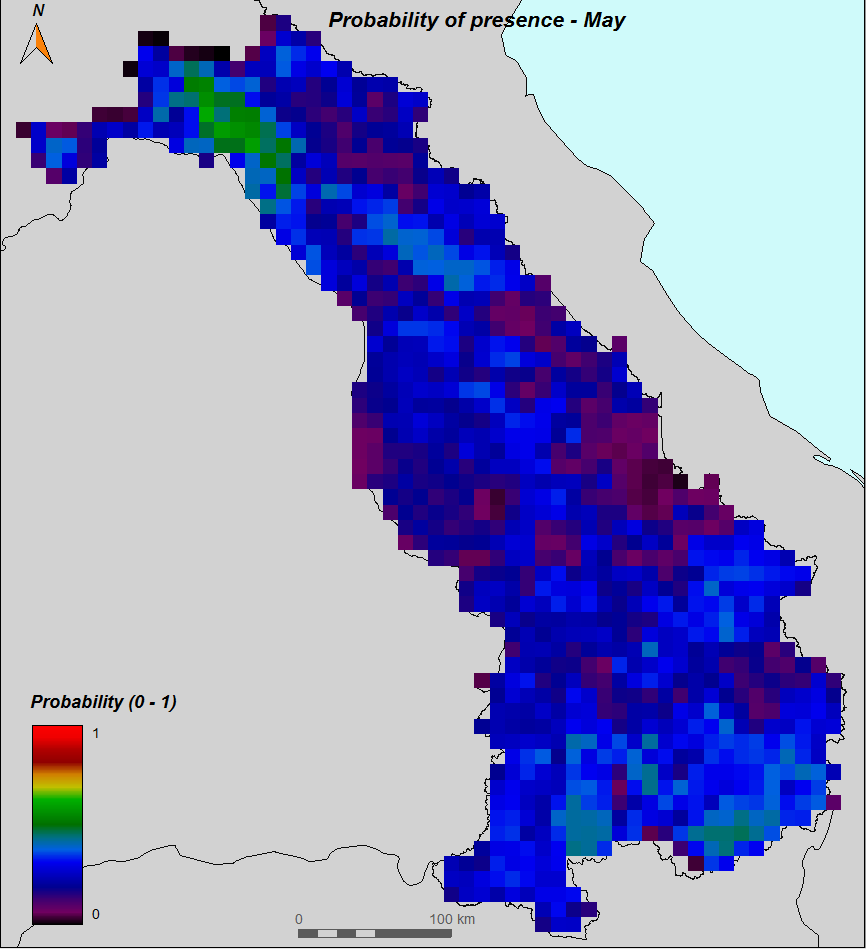

Supplement: S1 Fig — (ZIP) [file pone.0177274.s002.zip › Y4_05.tif]

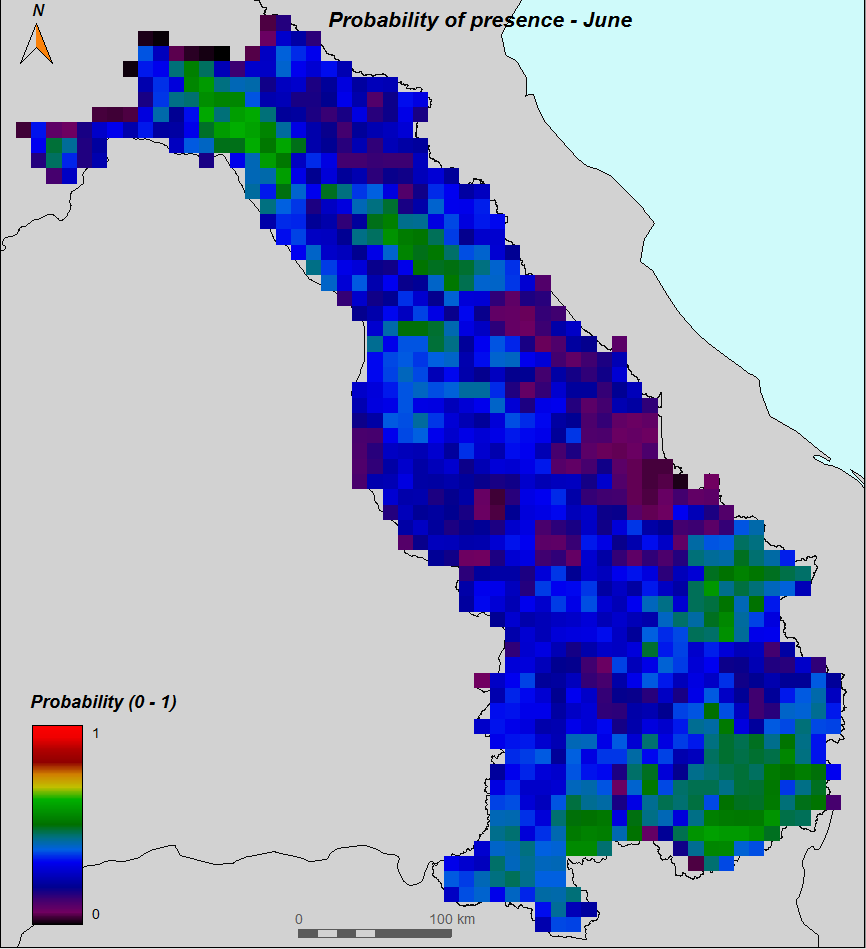

Supplement: S1 Fig — (ZIP) [file pone.0177274.s002.zip › Y4_06.tif]

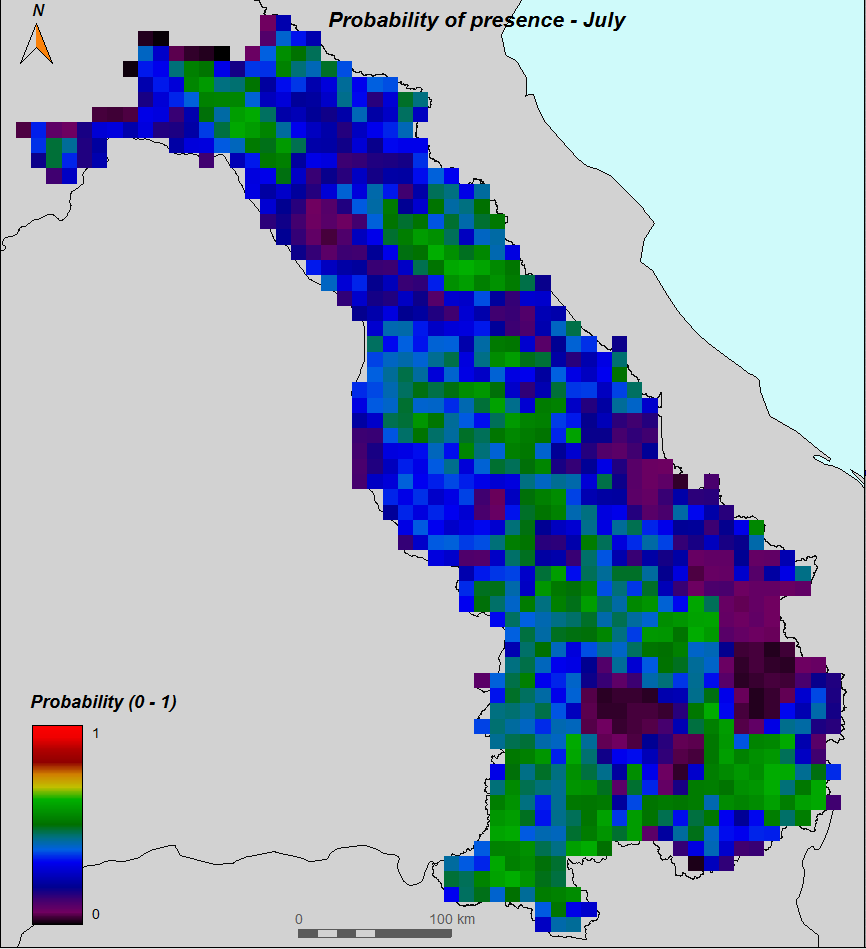

Supplement: S1 Fig — (ZIP) [file pone.0177274.s002.zip › Y4_07.tif]

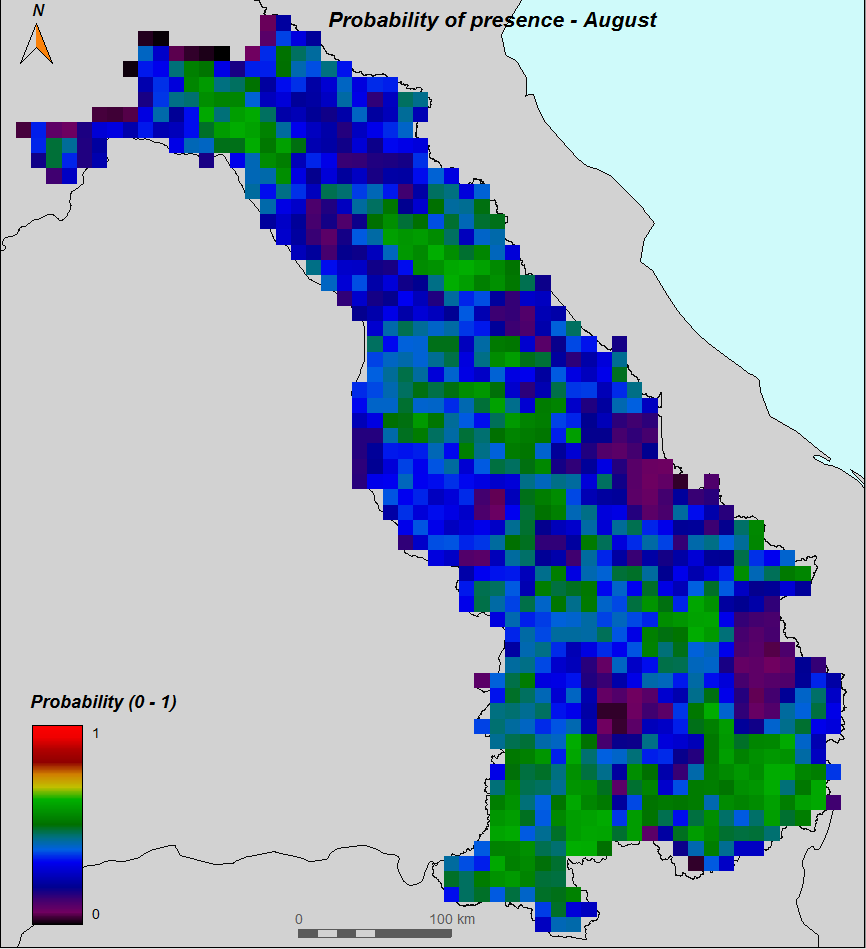

Supplement: S1 Fig — (ZIP) [file pone.0177274.s002.zip › Y4_08.tif]

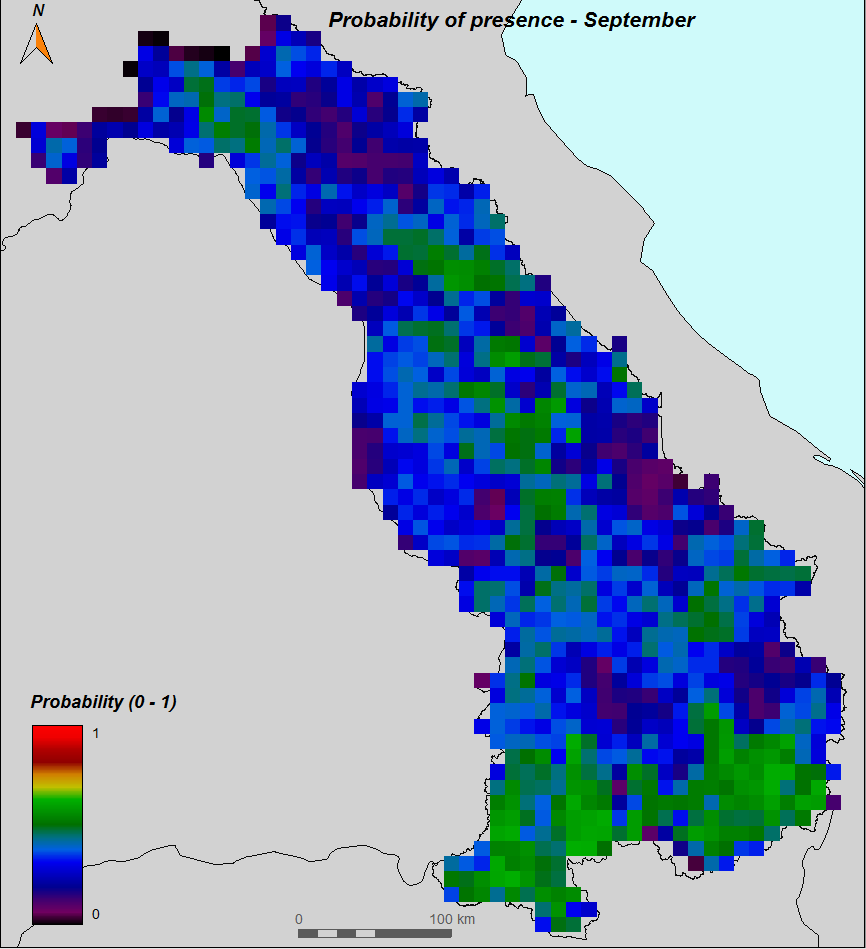

Supplement: S1 Fig — (ZIP) [file pone.0177274.s002.zip › Y4_09.tif]

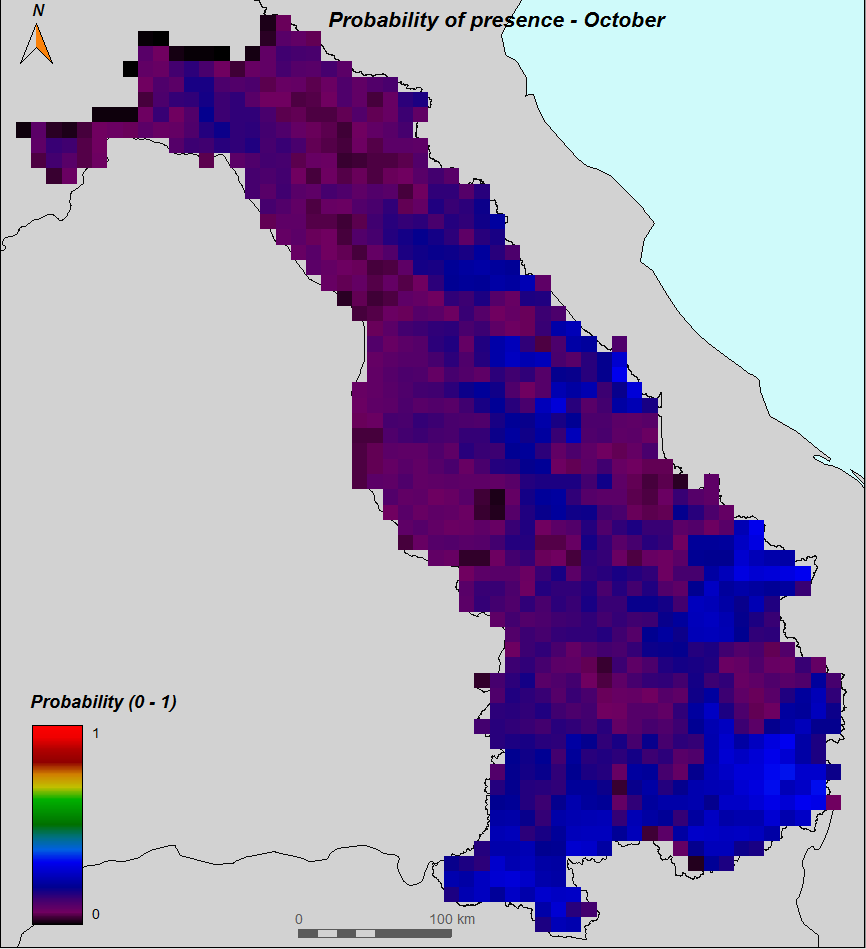

Supplement: S1 Fig — (ZIP) [file pone.0177274.s002.zip › Y4_10.tif]

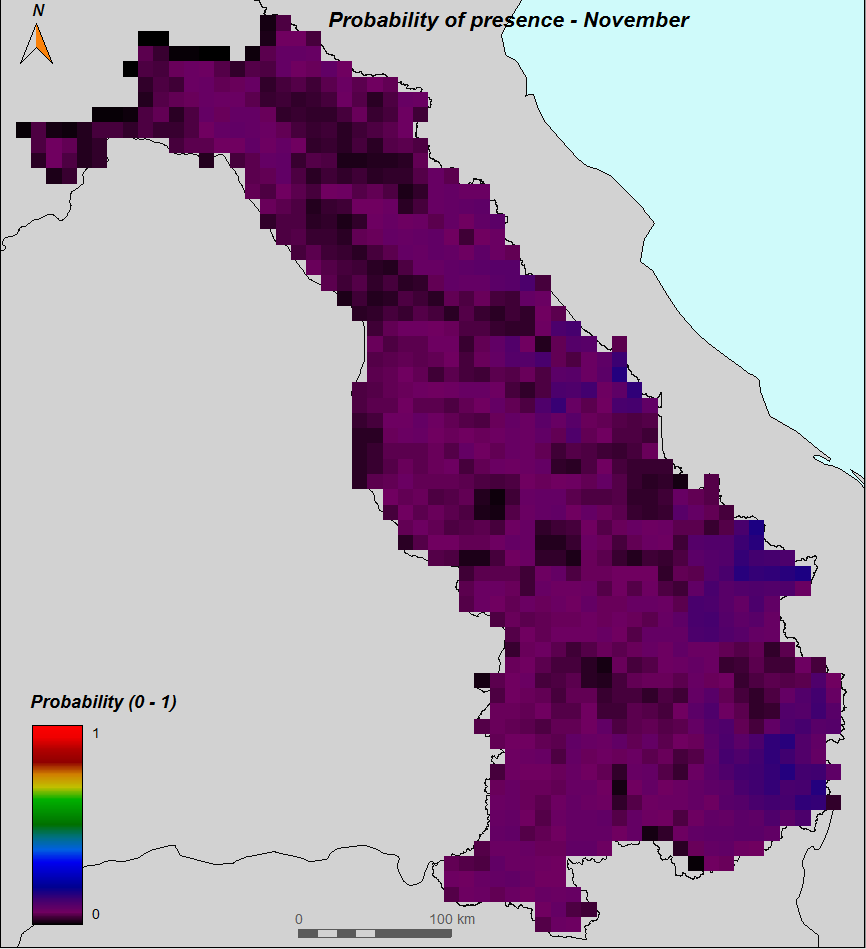

Supplement: S1 Fig — (ZIP) [file pone.0177274.s002.zip › Y4_11.tif]

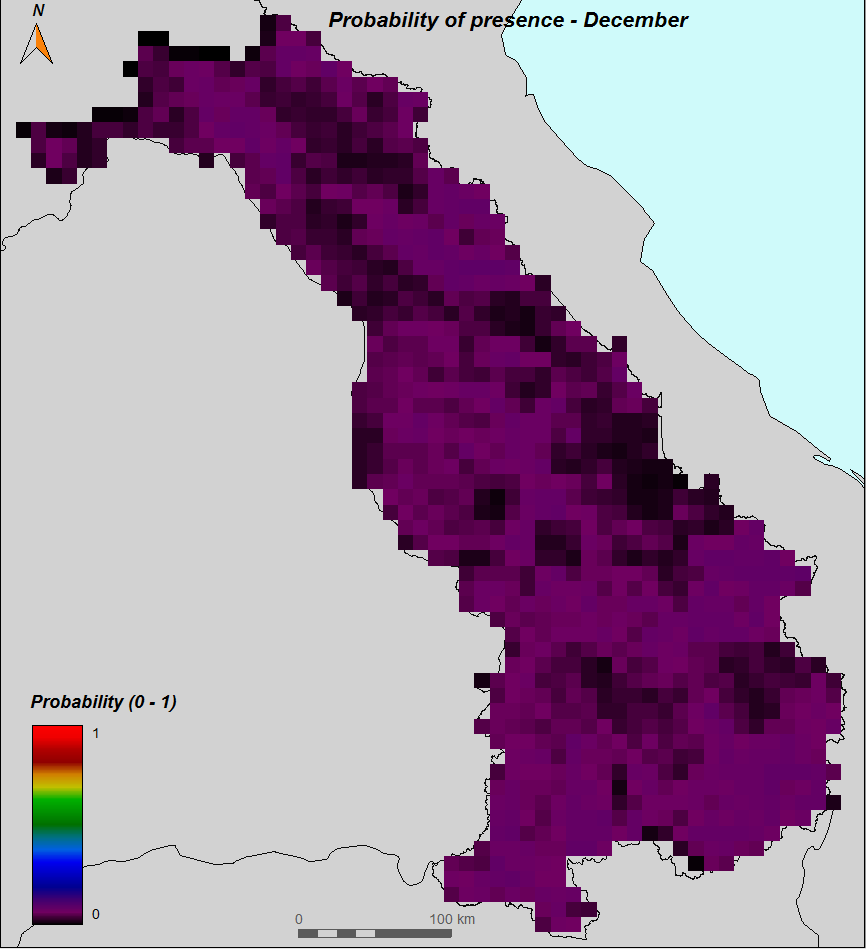

Supplement: S1 Fig — (ZIP) [file pone.0177274.s002.zip › Y4_12.tif]

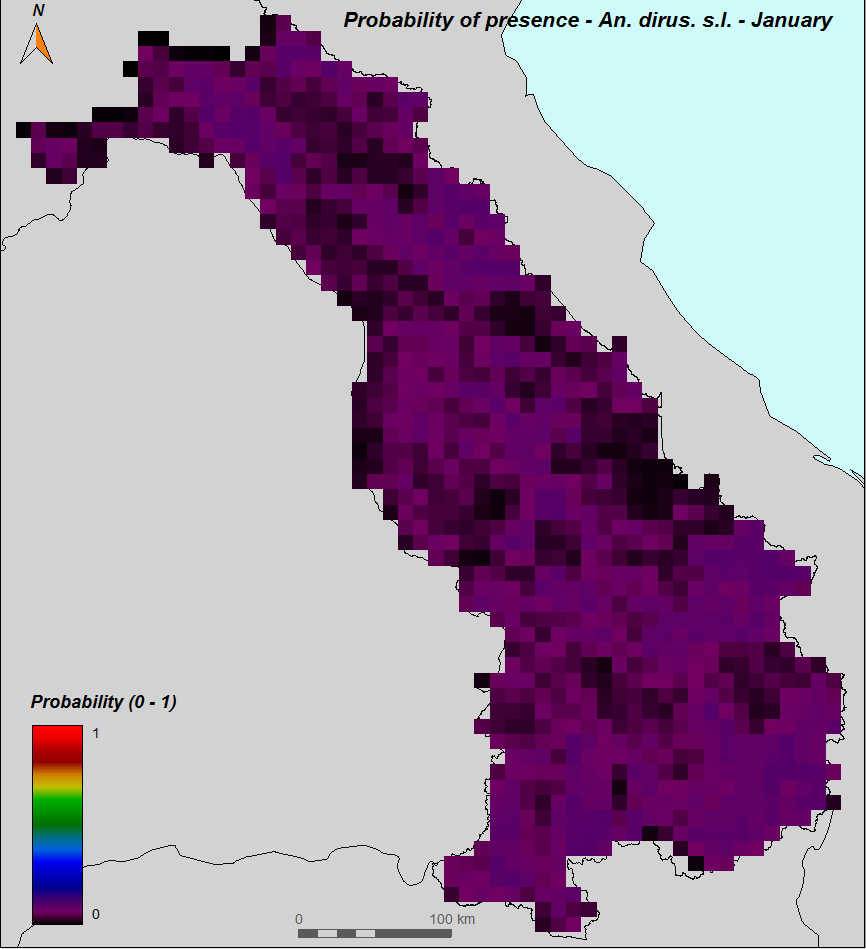

Supplement: S1 Fig — (ZIP) [file pone.0177274.s002.zip › Y4_Dirus_01.tif]

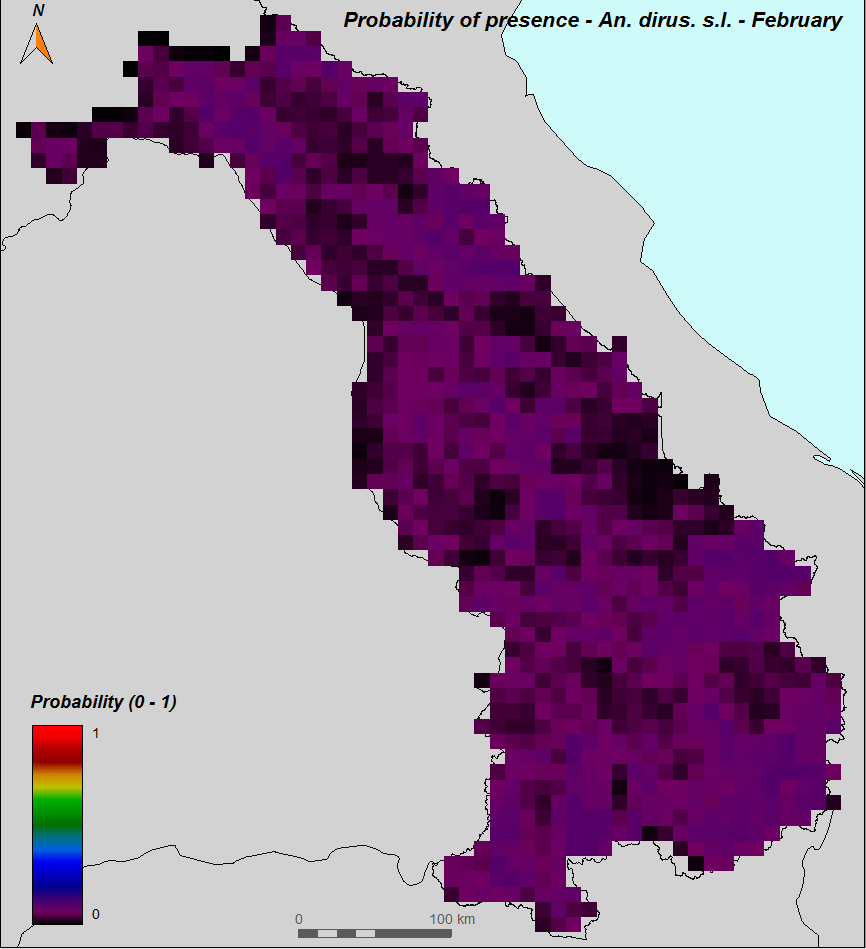

Supplement: S1 Fig — (ZIP) [file pone.0177274.s002.zip › Y4_Dirus_02.tif]

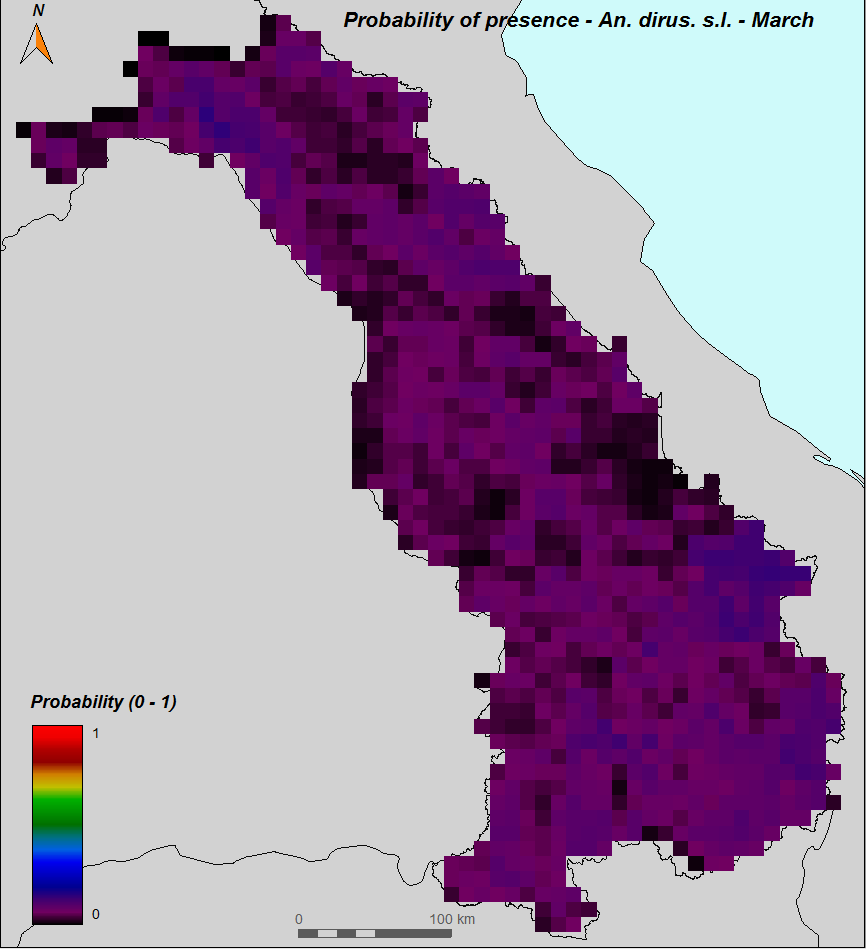

Supplement: S1 Fig — (ZIP) [file pone.0177274.s002.zip › Y4_Dirus_03.tif]

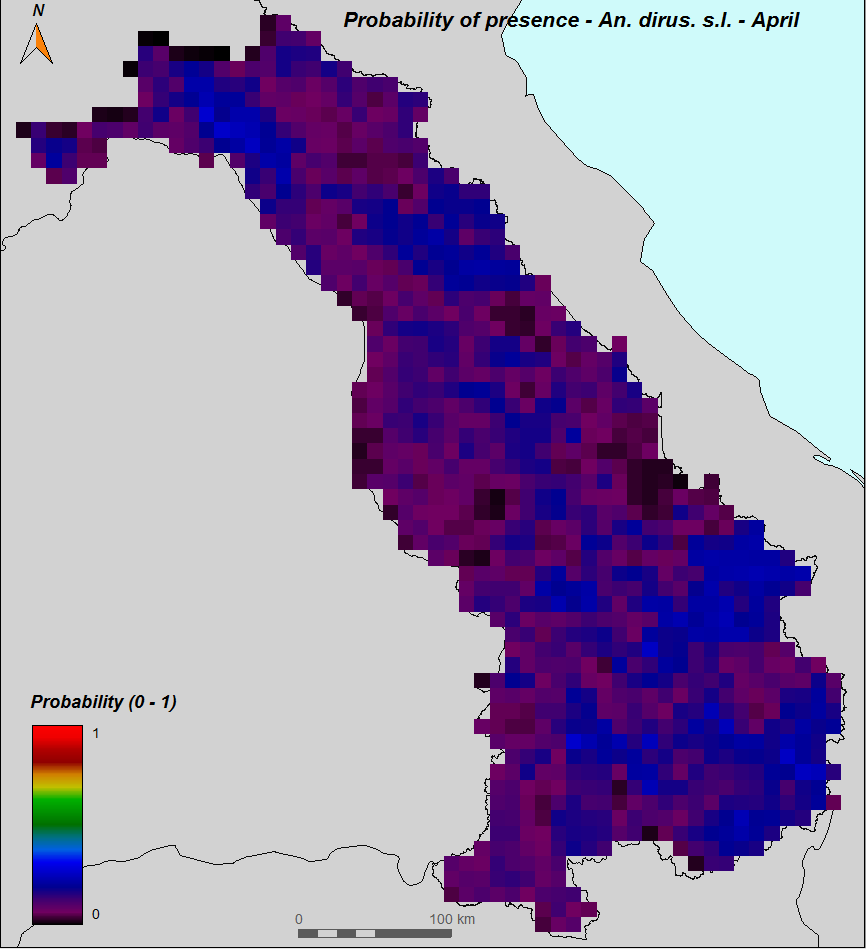

Supplement: S1 Fig — (ZIP) [file pone.0177274.s002.zip › Y4_Dirus_04.tif]

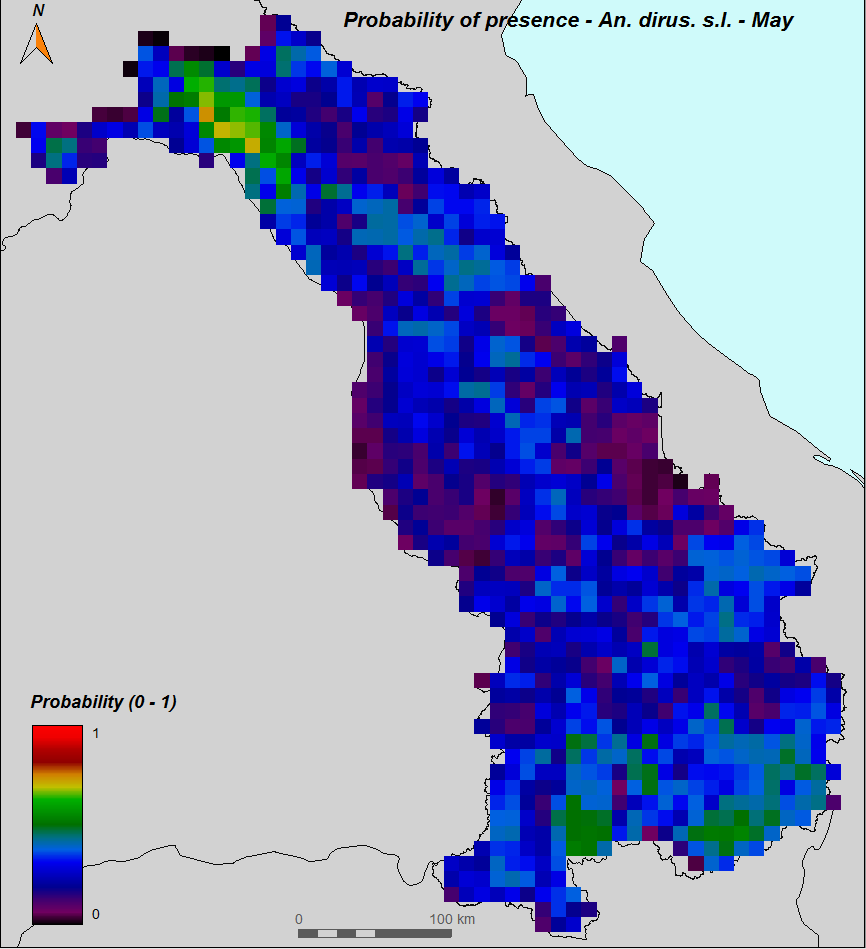

Supplement: S1 Fig — (ZIP) [file pone.0177274.s002.zip › Y4_Dirus_05.tif]

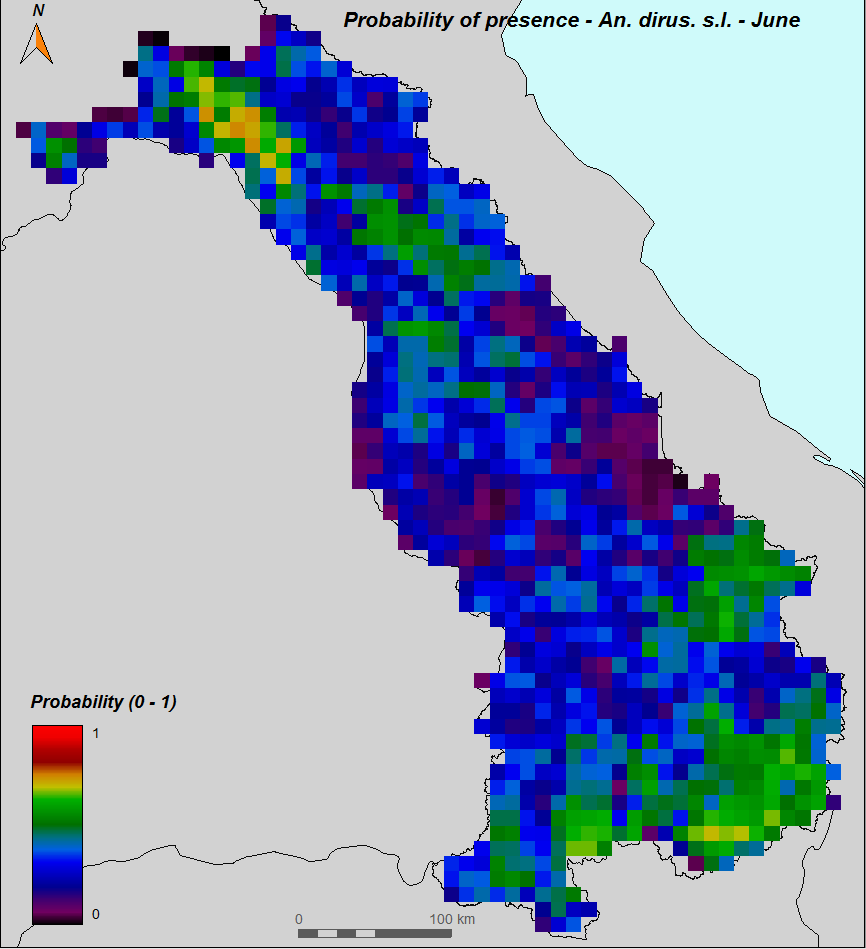

Supplement: S1 Fig — (ZIP) [file pone.0177274.s002.zip › Y4_Dirus_06.tif]

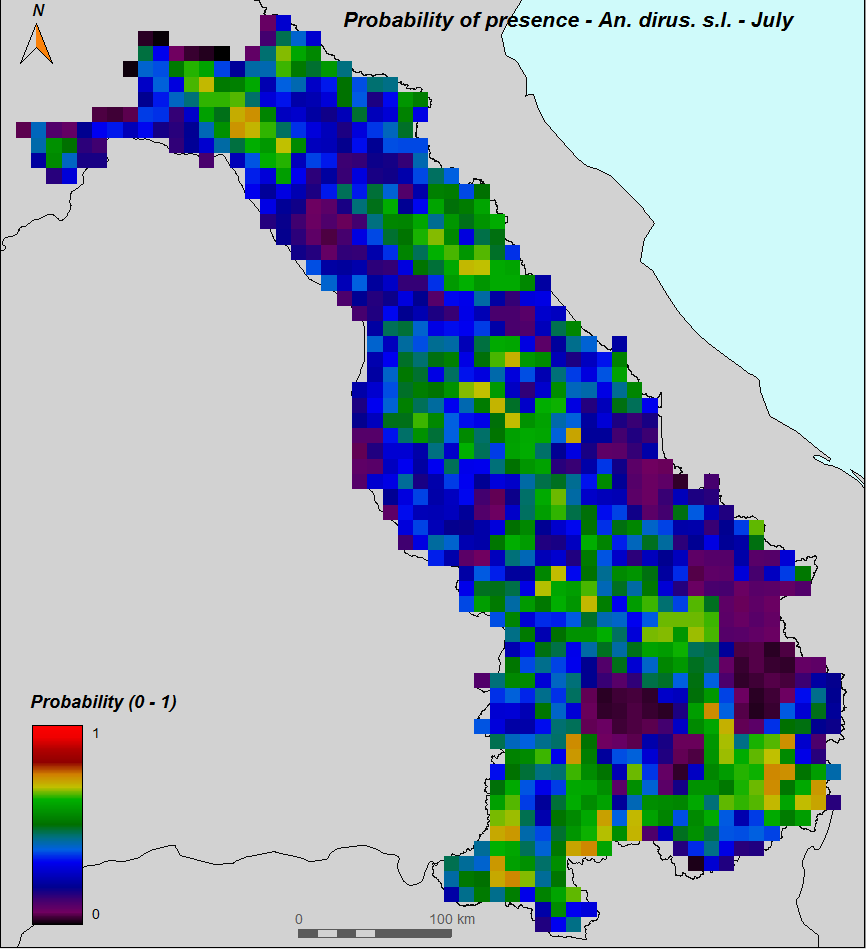

Supplement: S1 Fig — (ZIP) [file pone.0177274.s002.zip › Y4_Dirus_07.tif]

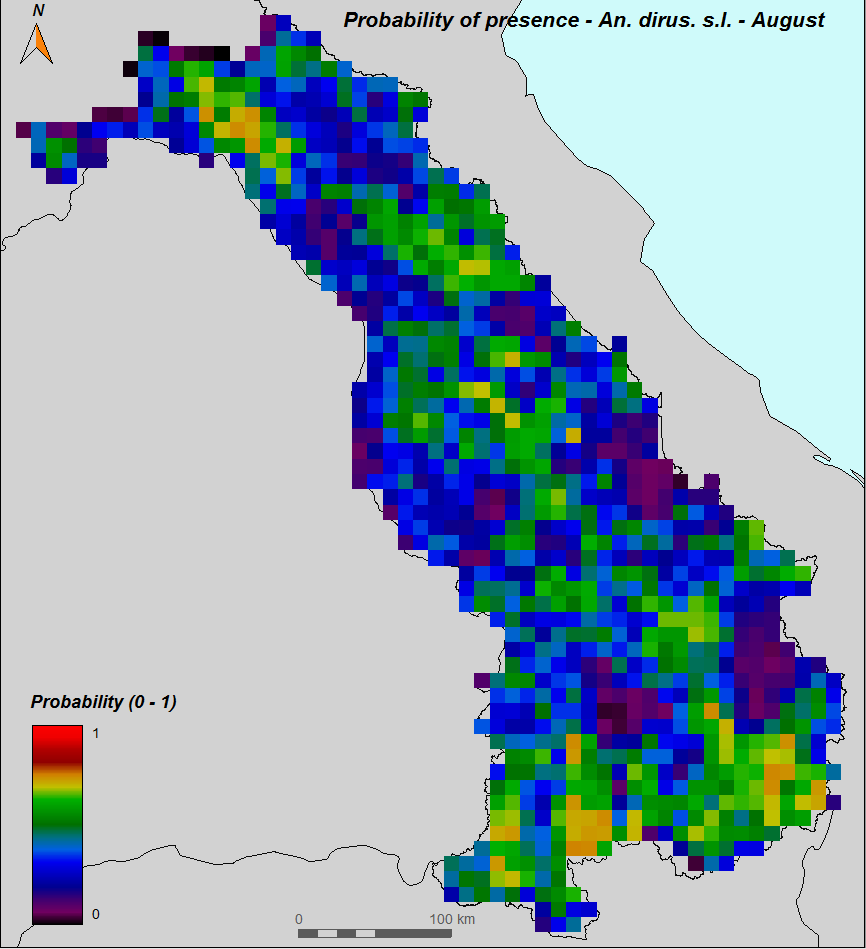

Supplement: S1 Fig — (ZIP) [file pone.0177274.s002.zip › Y4_Dirus_08.tif]

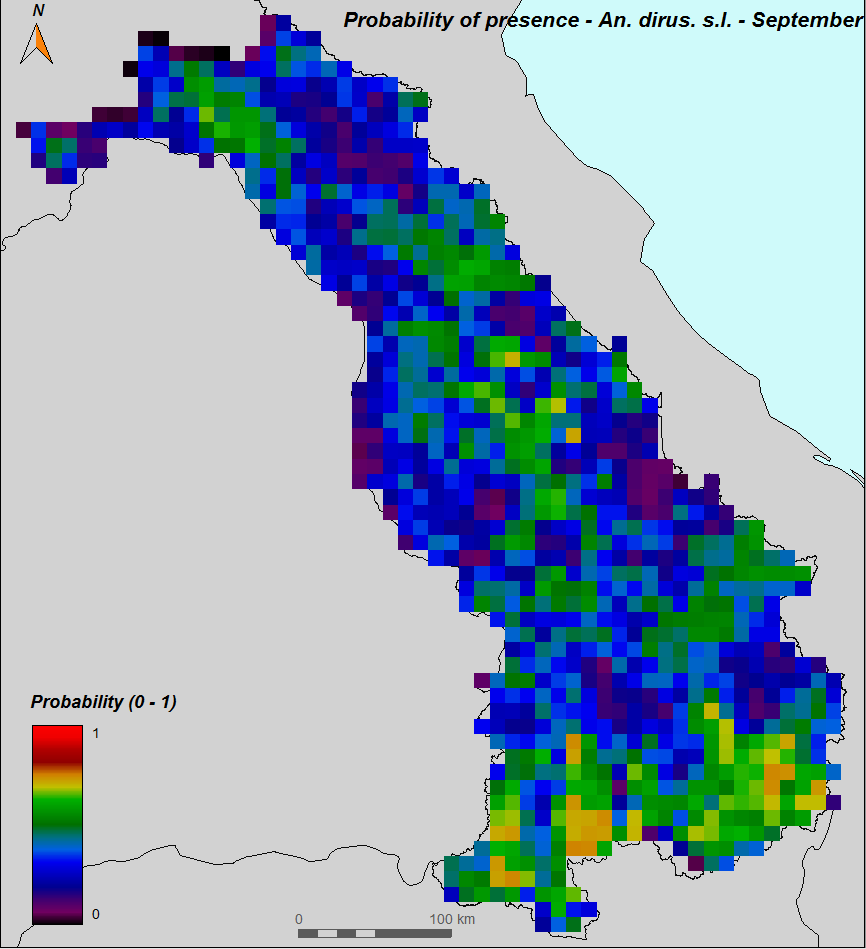

Supplement: S1 Fig — (ZIP) [file pone.0177274.s002.zip › Y4_Dirus_09.tif]

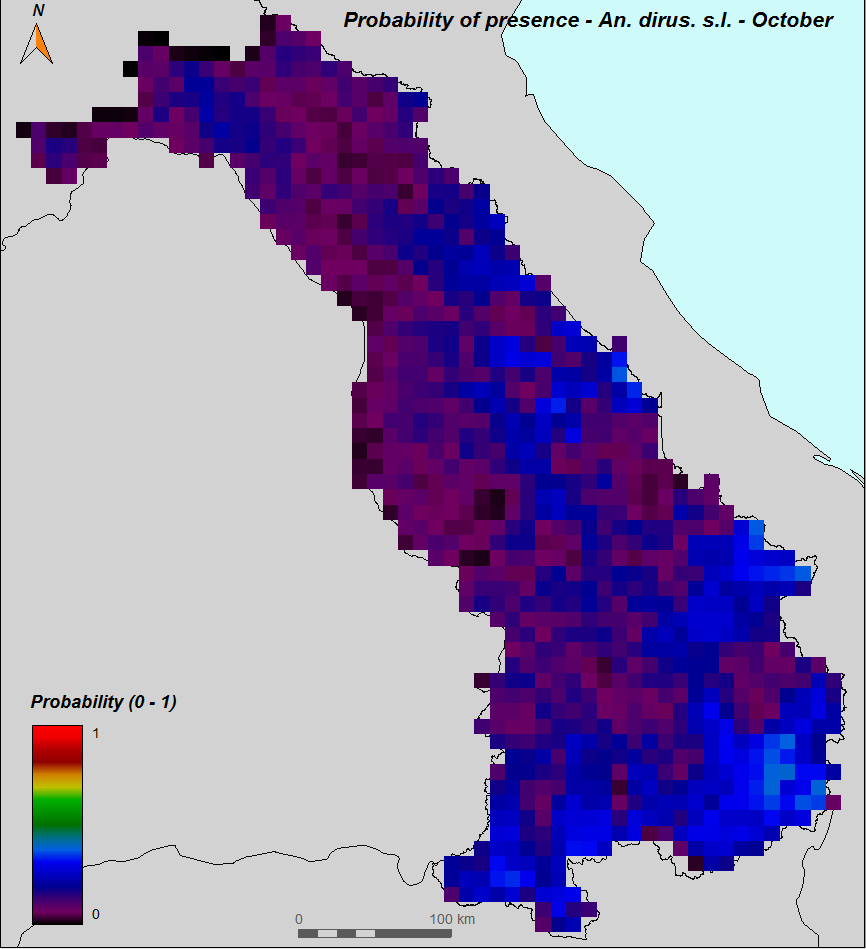

Supplement: S1 Fig — (ZIP) [file pone.0177274.s002.zip › Y4_Dirus_10.tif]

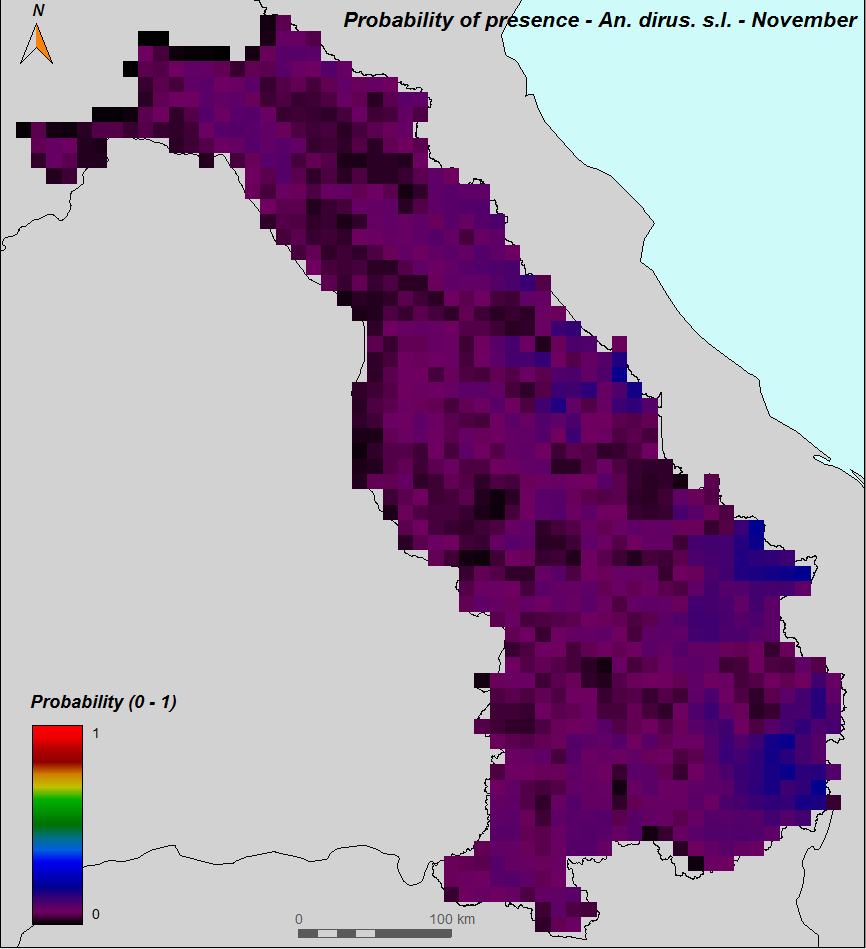

Supplement: S1 Fig — (ZIP) [file pone.0177274.s002.zip › Y4_Dirus_11.tif]

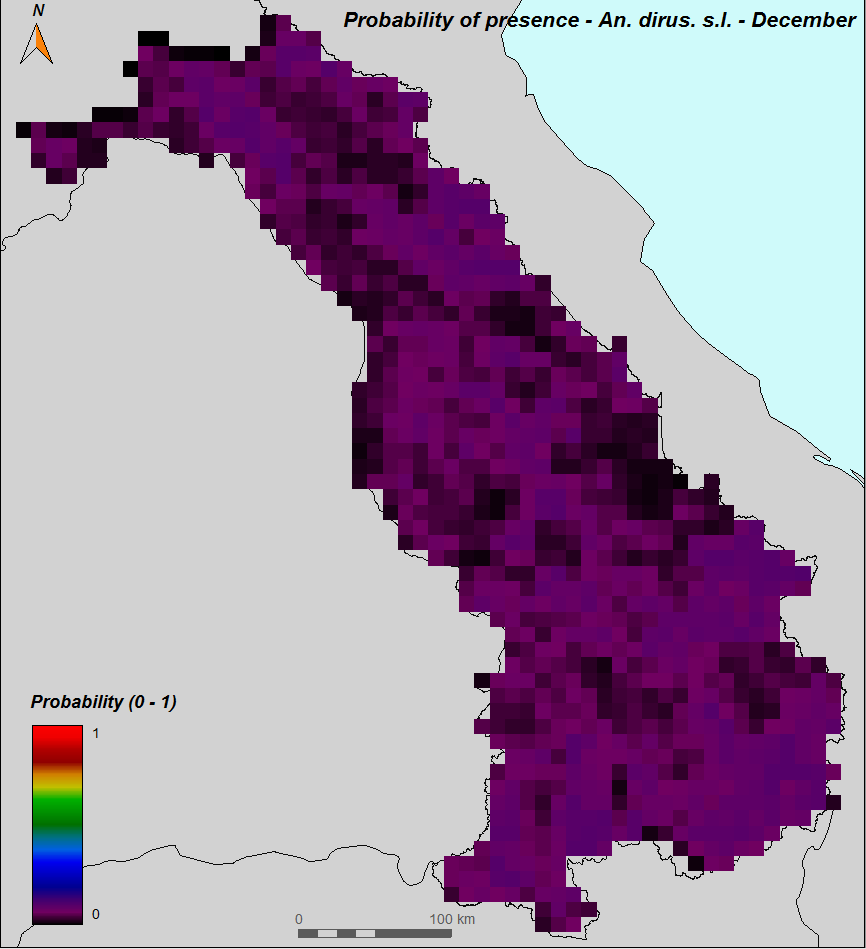

Supplement: S1 Fig — (ZIP) [file pone.0177274.s002.zip › Y4_Dirus_12.tif]

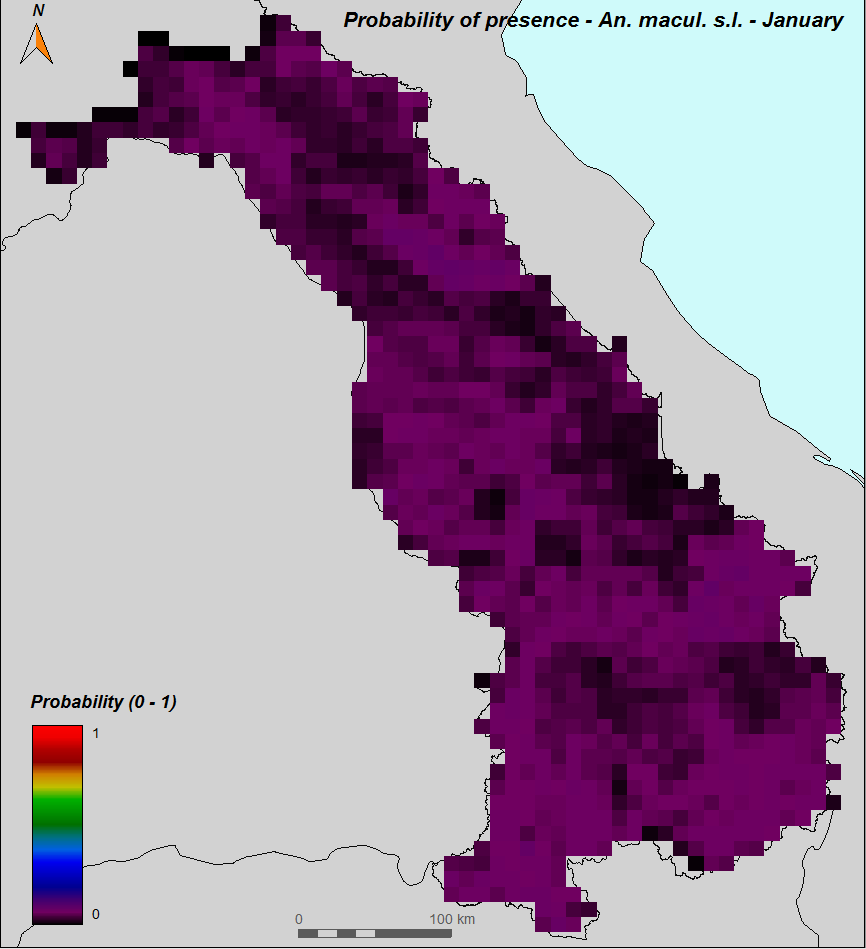

Supplement: S1 Fig — (ZIP) [file pone.0177274.s002.zip › Y4_Macul_01.tif]

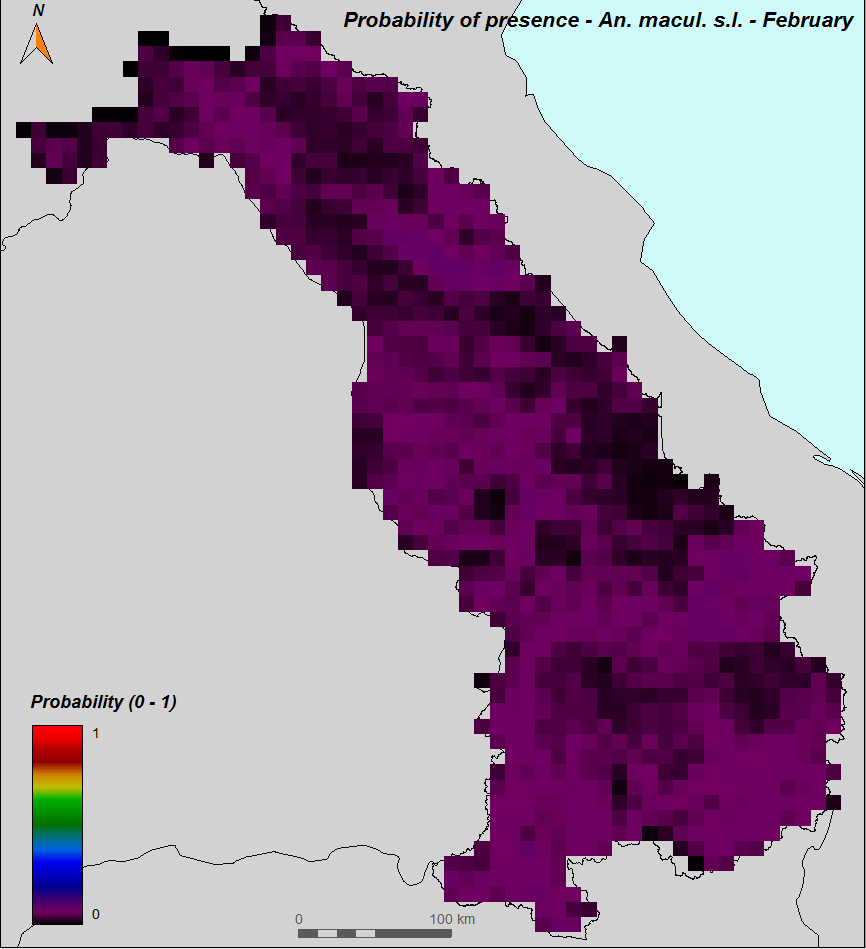

Supplement: S1 Fig — (ZIP) [file pone.0177274.s002.zip › Y4_Macul_02.tif]

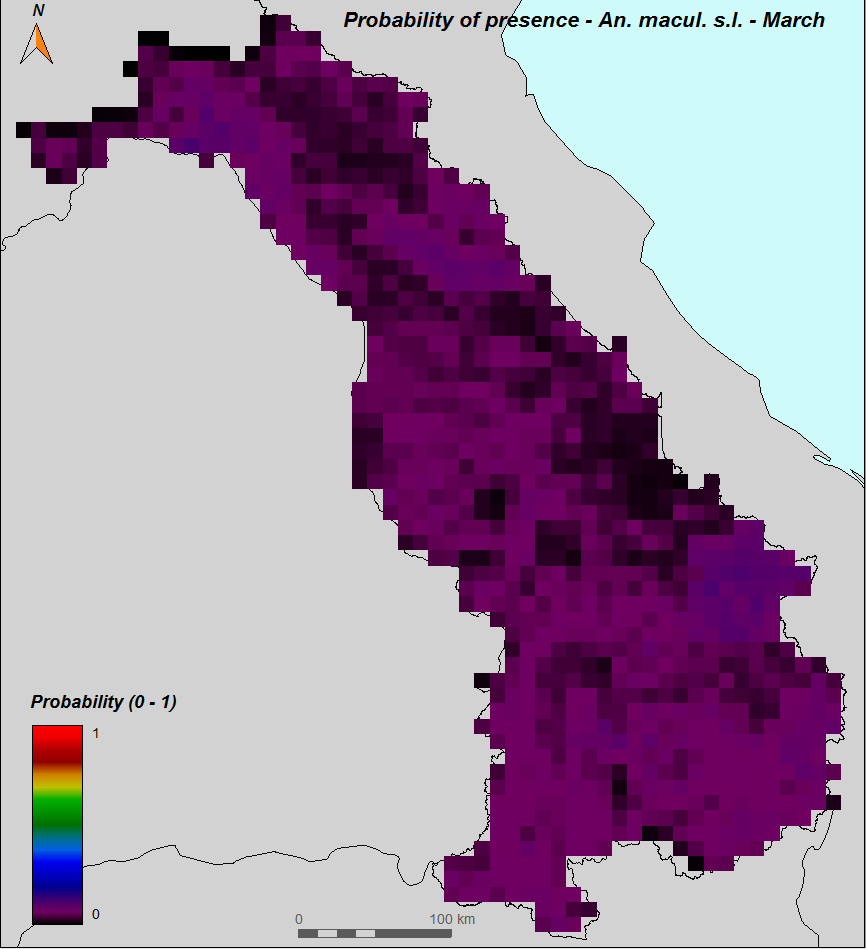

Supplement: S1 Fig — (ZIP) [file pone.0177274.s002.zip › Y4_Macul_03.tif]

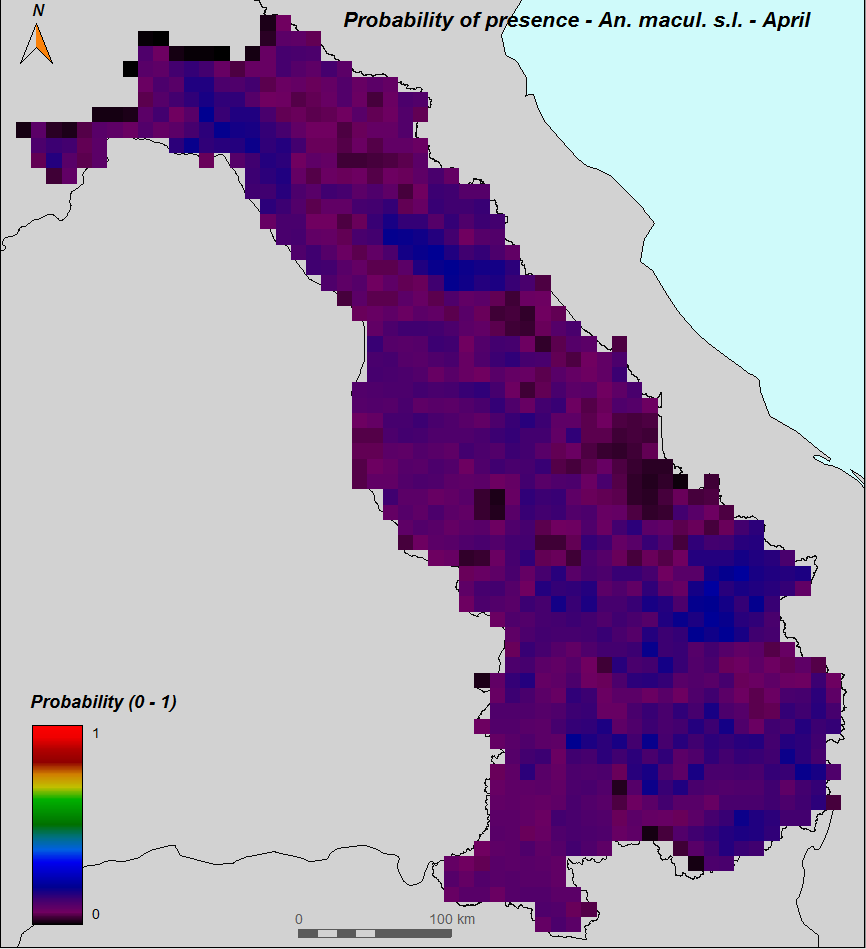

Supplement: S1 Fig — (ZIP) [file pone.0177274.s002.zip › Y4_Macul_04.tif]

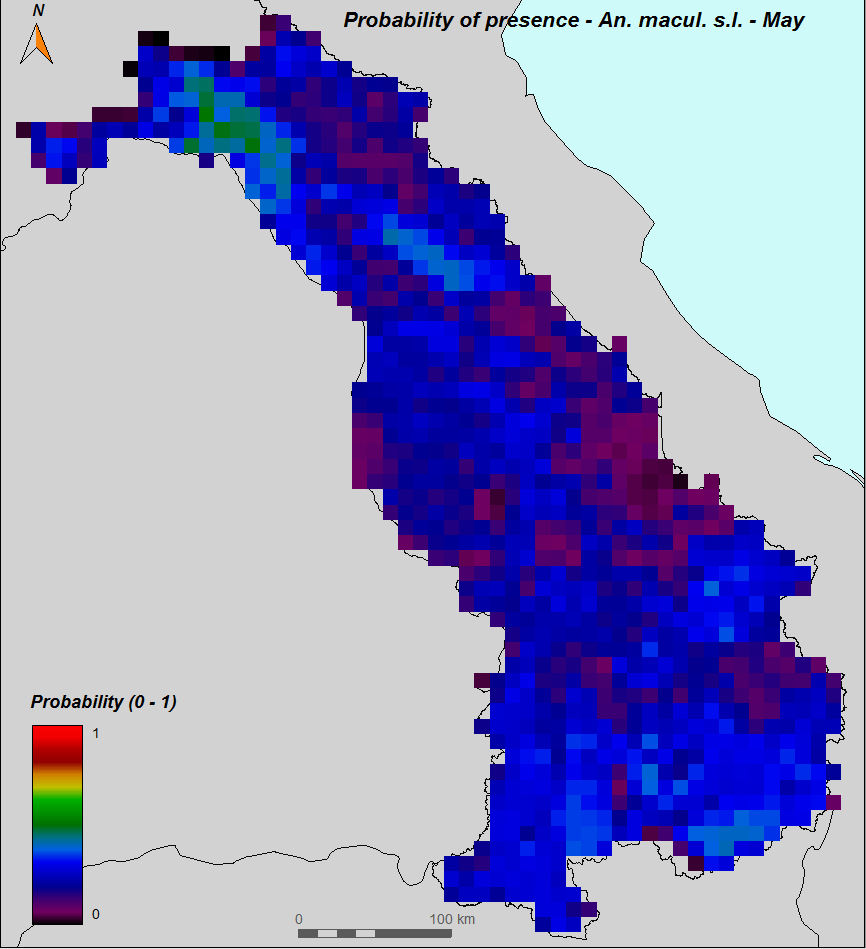

Supplement: S1 Fig — (ZIP) [file pone.0177274.s002.zip › Y4_Macul_05.tif]

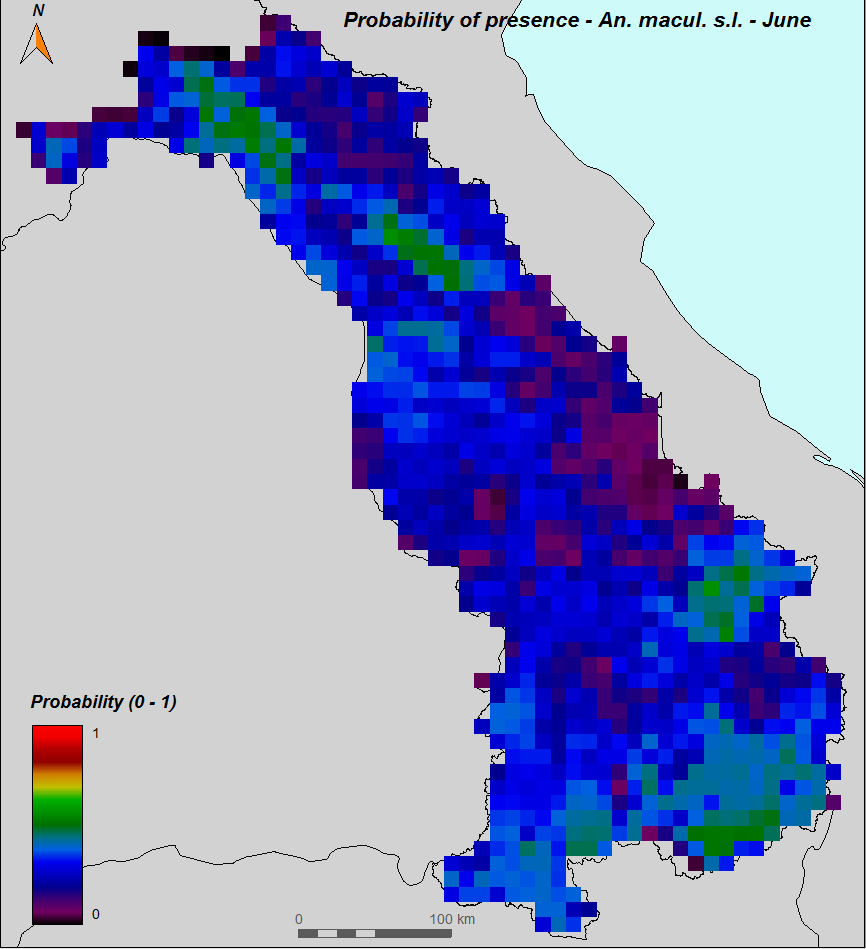

Supplement: S1 Fig — (ZIP) [file pone.0177274.s002.zip › Y4_Macul_06.tif]

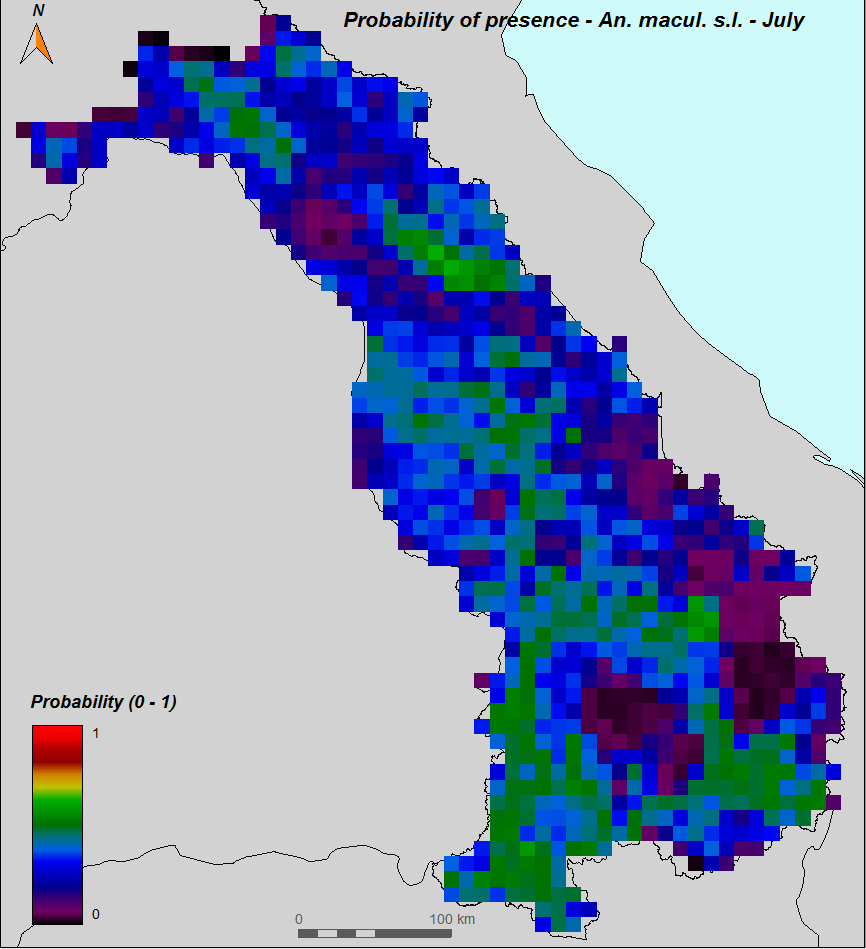

Supplement: S1 Fig — (ZIP) [file pone.0177274.s002.zip › Y4_Macul_07.tif]

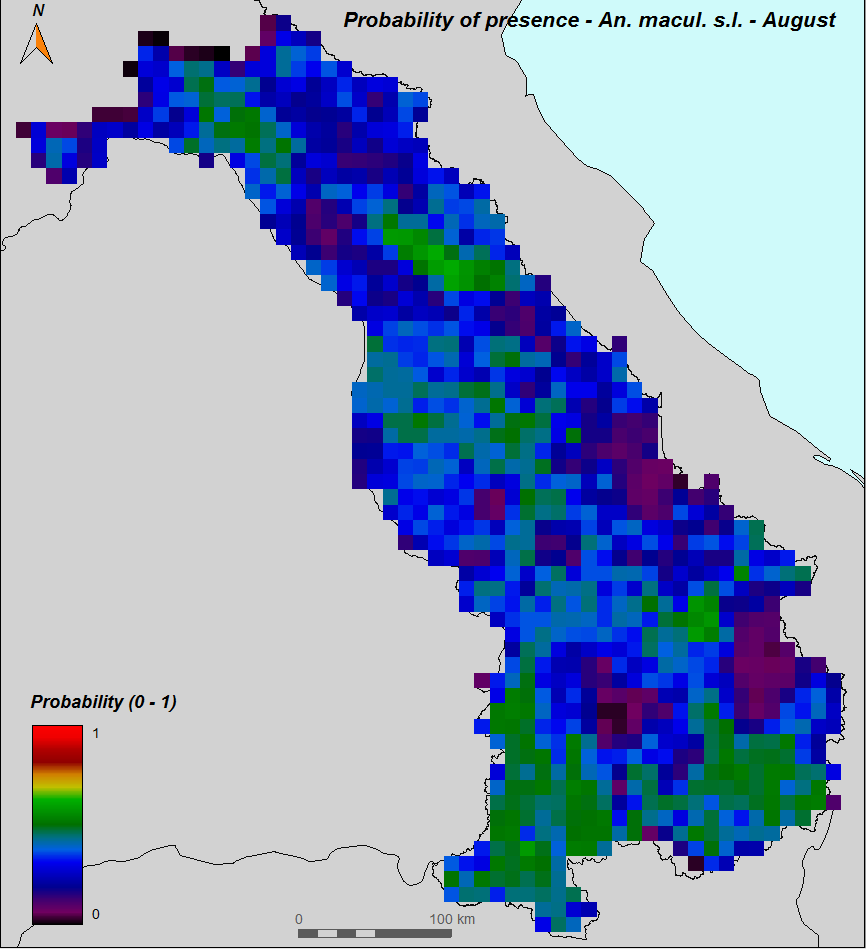

Supplement: S1 Fig — (ZIP) [file pone.0177274.s002.zip › Y4_Macul_08.tif]

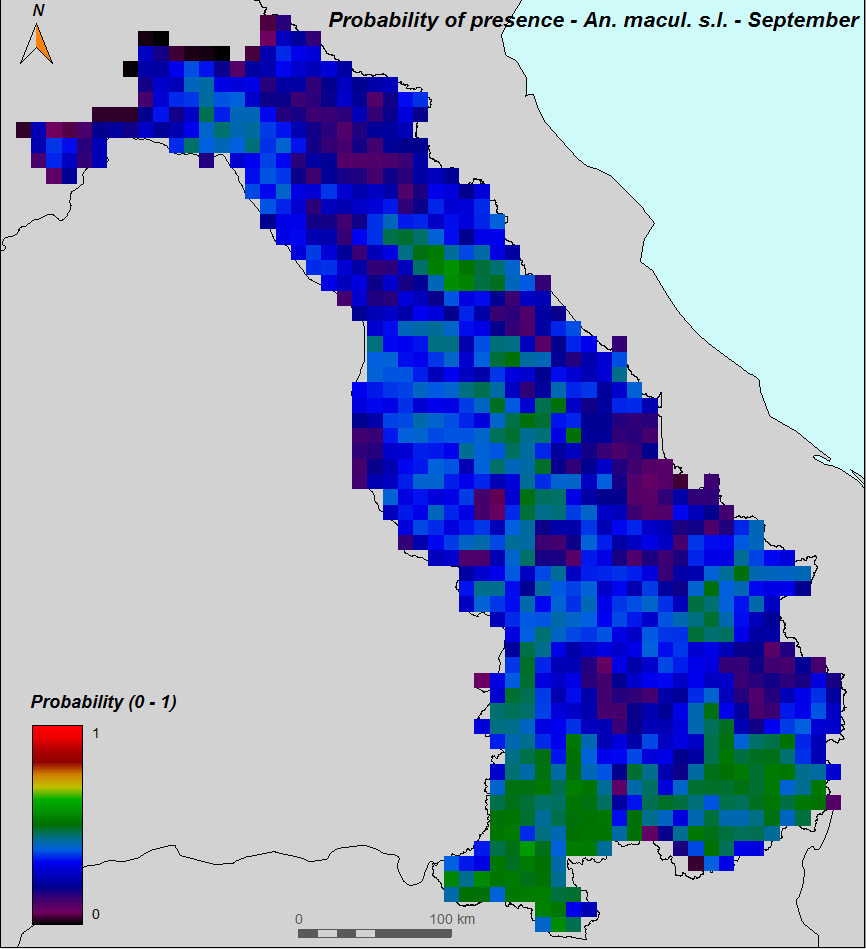

Supplement: S1 Fig — (ZIP) [file pone.0177274.s002.zip › Y4_Macul_09.tif]

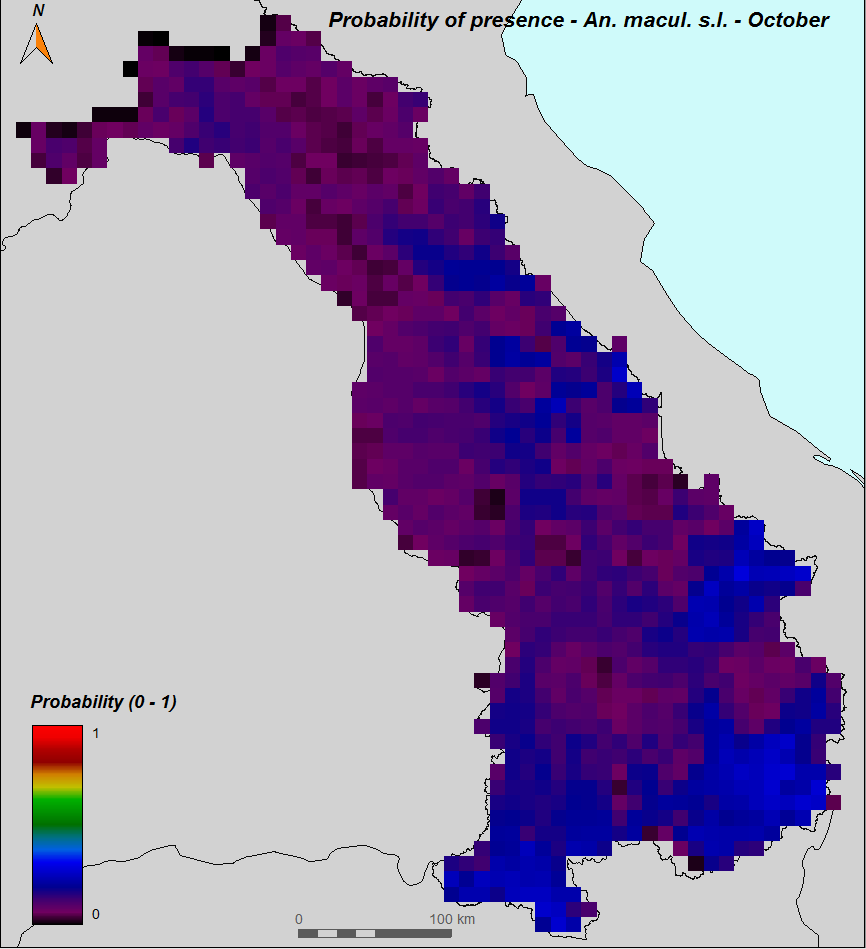

Supplement: S1 Fig — (ZIP) [file pone.0177274.s002.zip › Y4_Macul_10.tif]

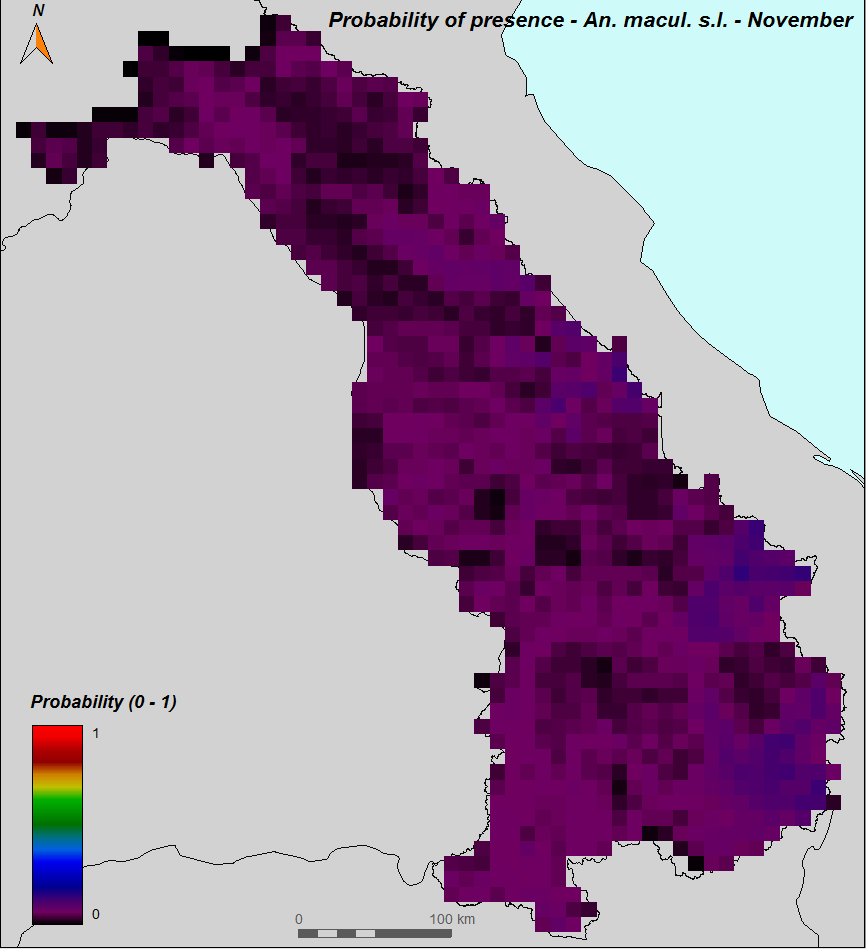

Supplement: S1 Fig — (ZIP) [file pone.0177274.s002.zip › Y4_Macul_11.tif]

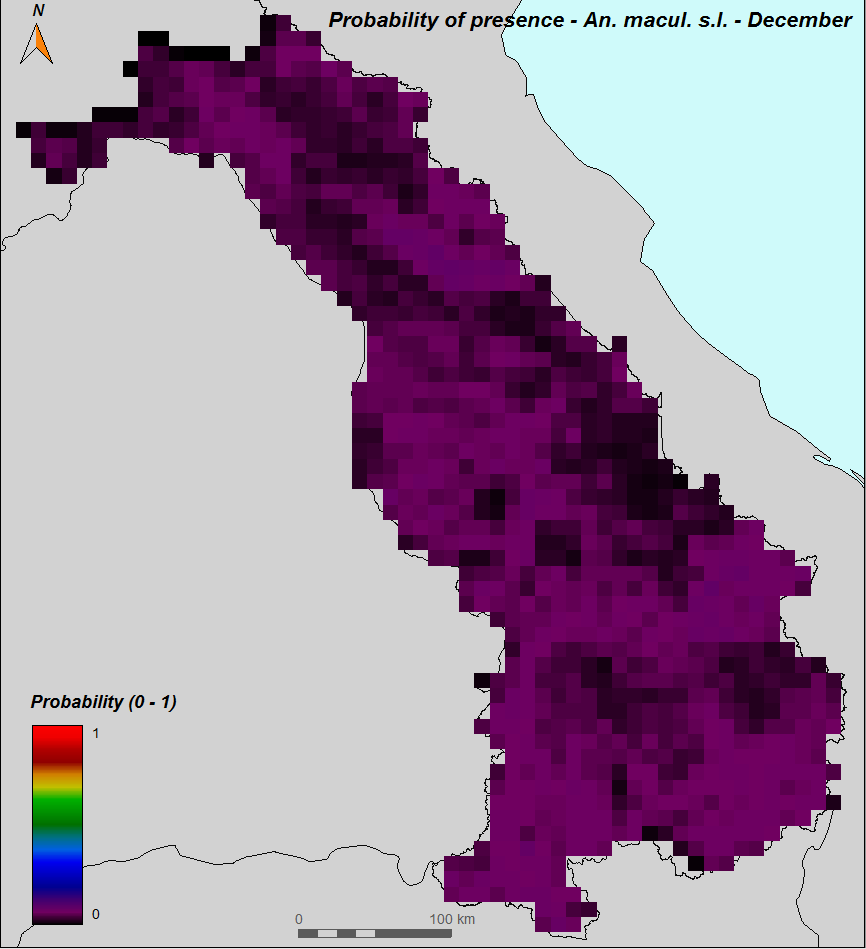

Supplement: S1 Fig — (ZIP) [file pone.0177274.s002.zip › Y4_Macul_12.tif]

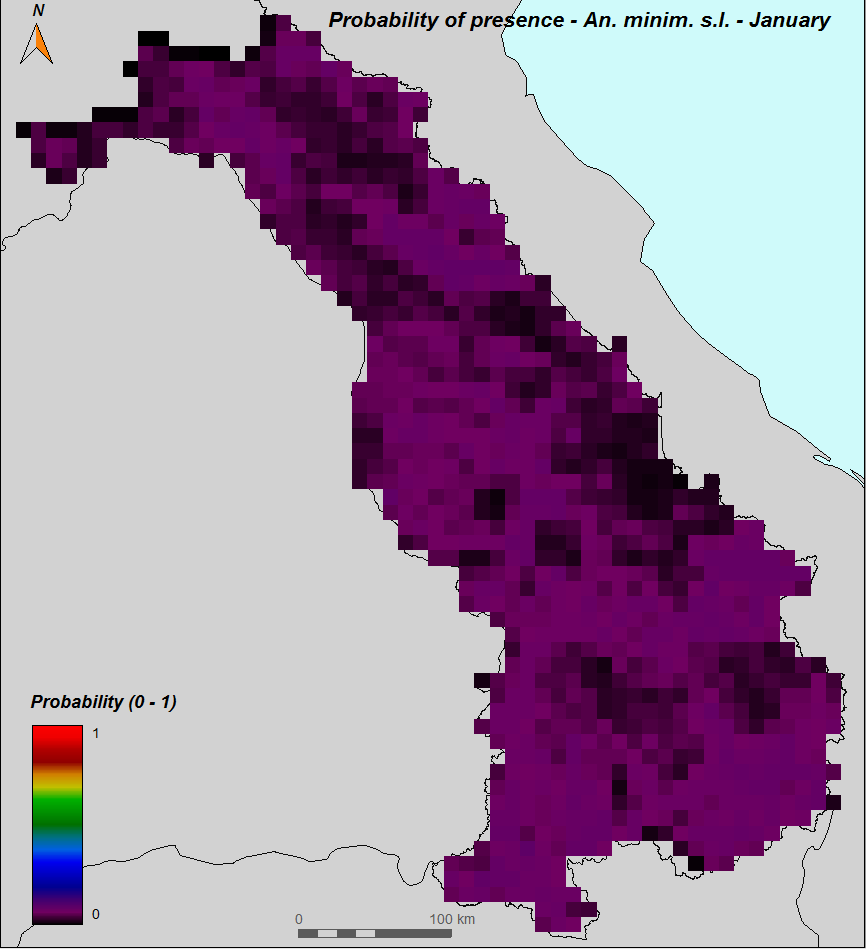

Supplement: S1 Fig — (ZIP) [file pone.0177274.s002.zip › Y4_Minim_01.tif]

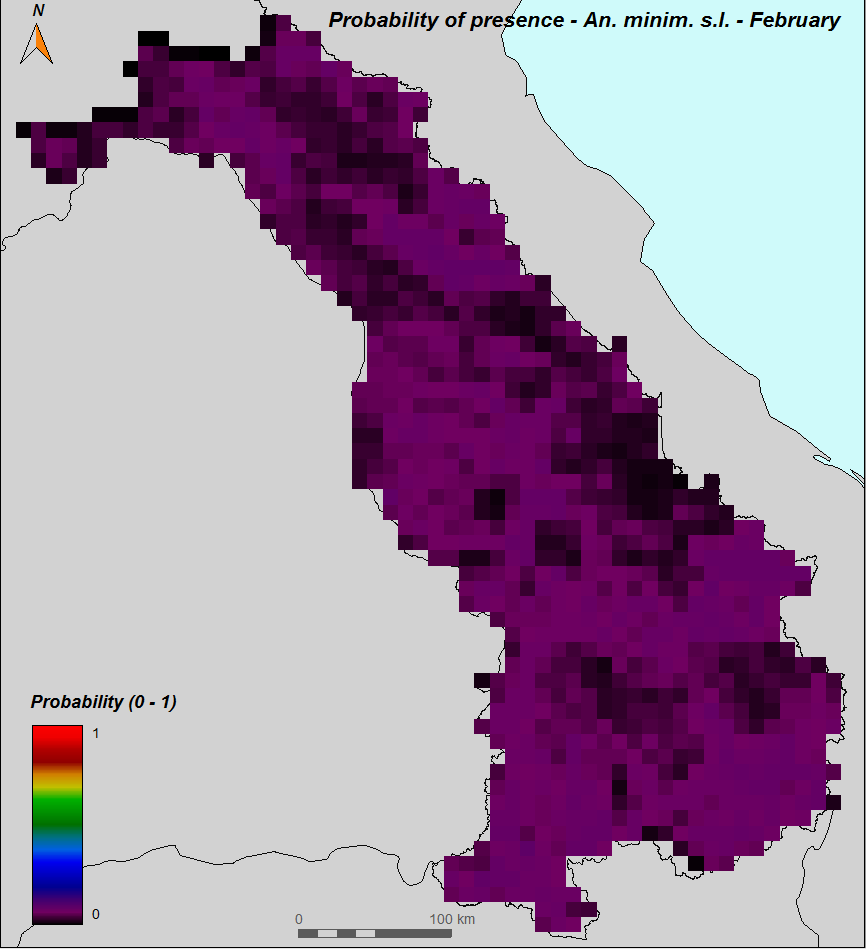

Supplement: S1 Fig — (ZIP) [file pone.0177274.s002.zip › Y4_Minim_02.tif]

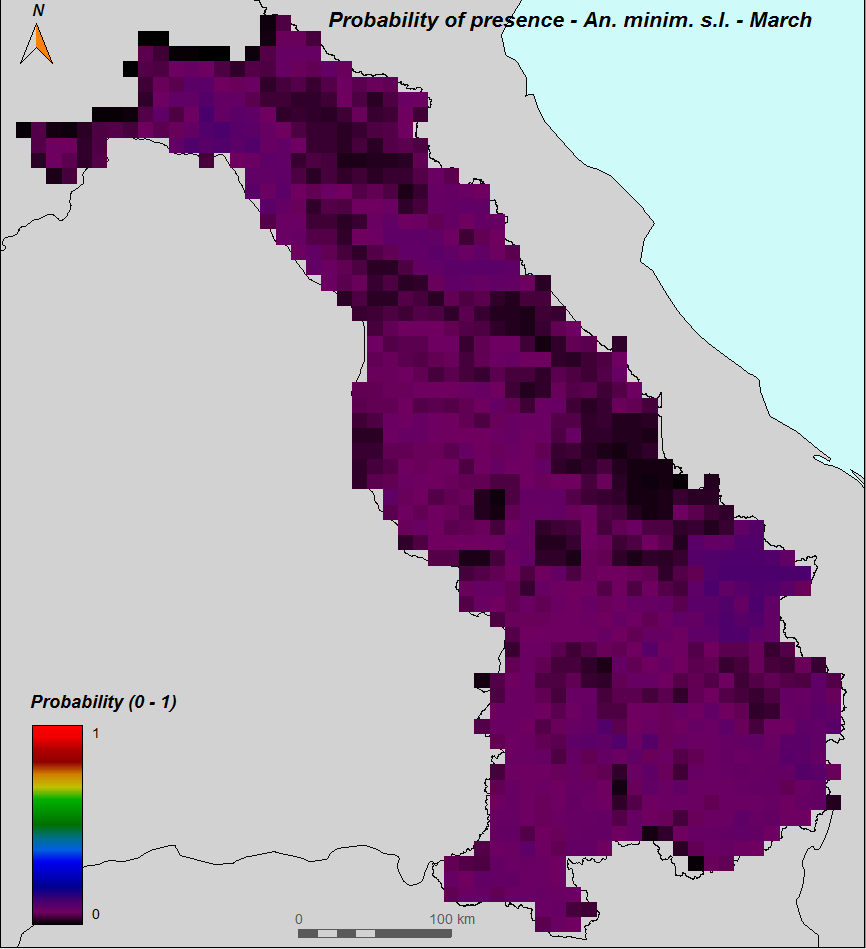

Supplement: S1 Fig — (ZIP) [file pone.0177274.s002.zip › Y4_Minim_03.tif]

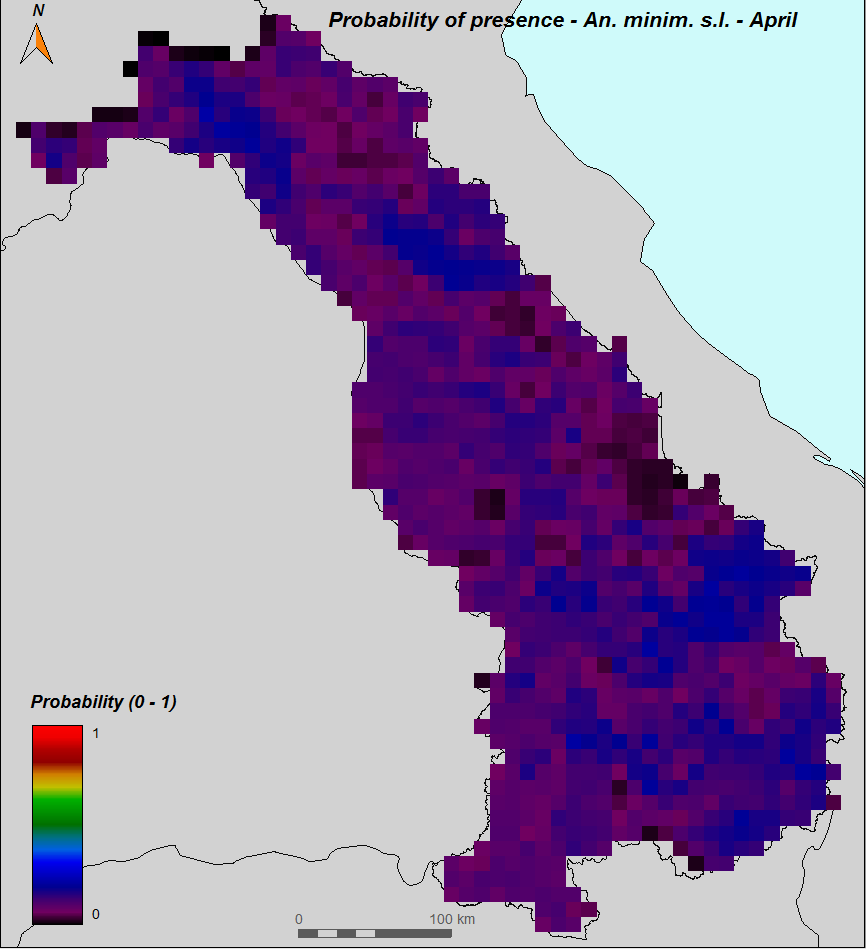

Supplement: S1 Fig — (ZIP) [file pone.0177274.s002.zip › Y4_Minim_04.tif]

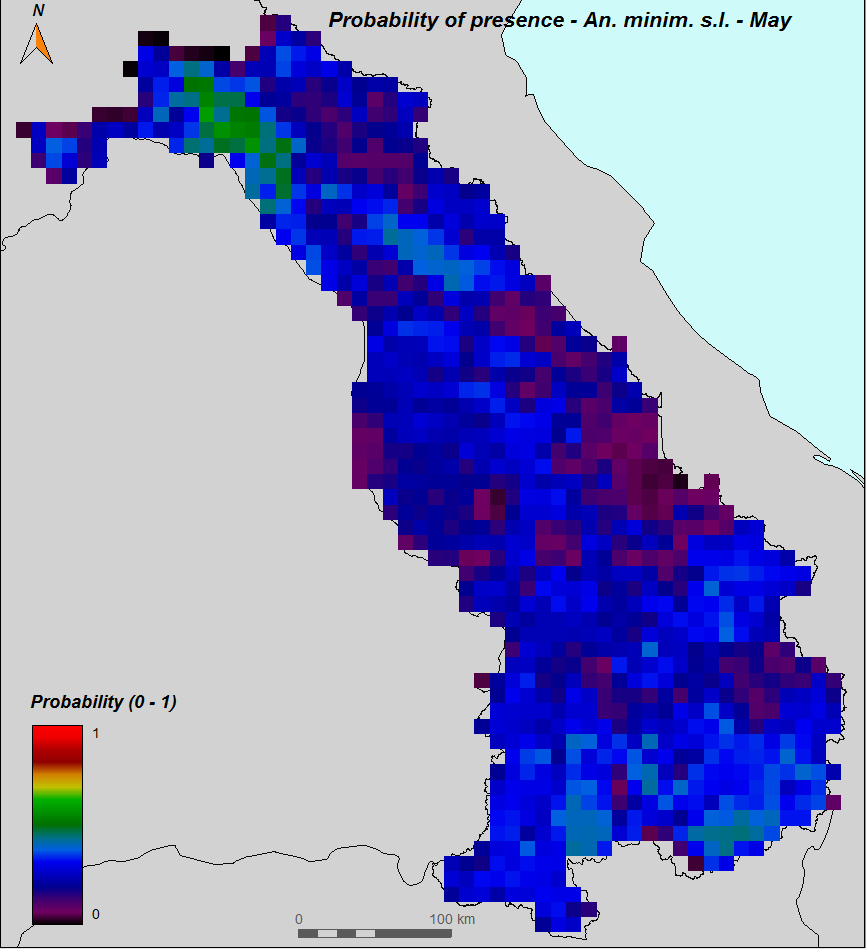

Supplement: S1 Fig — (ZIP) [file pone.0177274.s002.zip › Y4_Minim_05.tif]

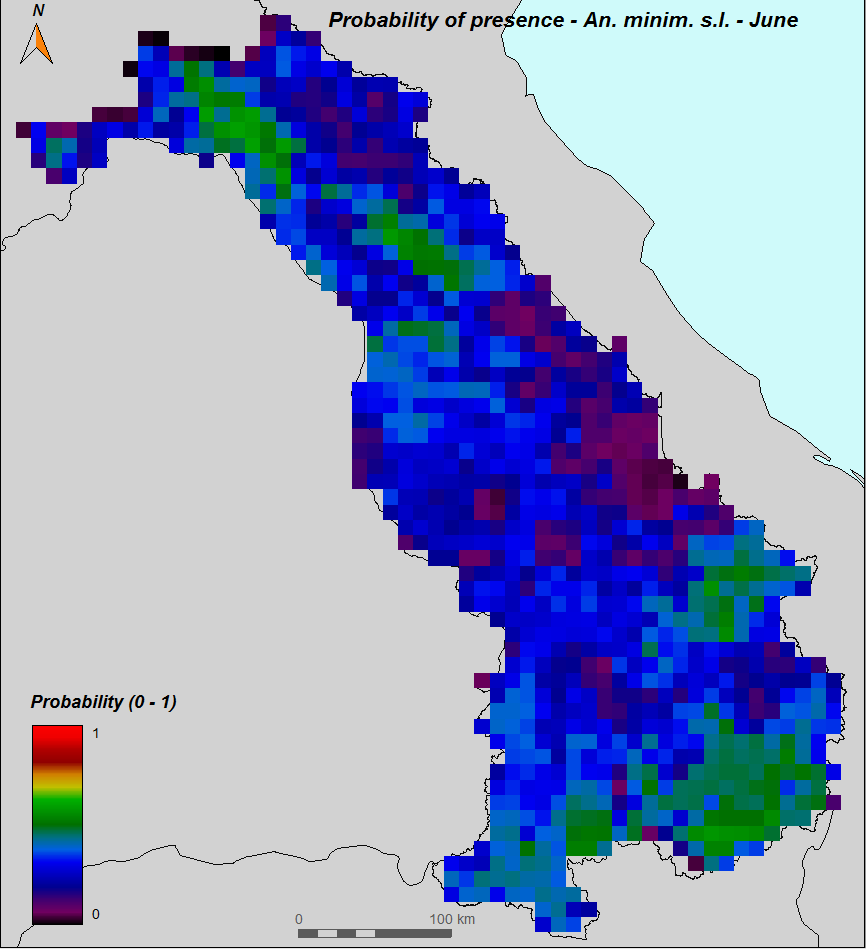

Supplement: S1 Fig — (ZIP) [file pone.0177274.s002.zip › Y4_Minim_06.tif]

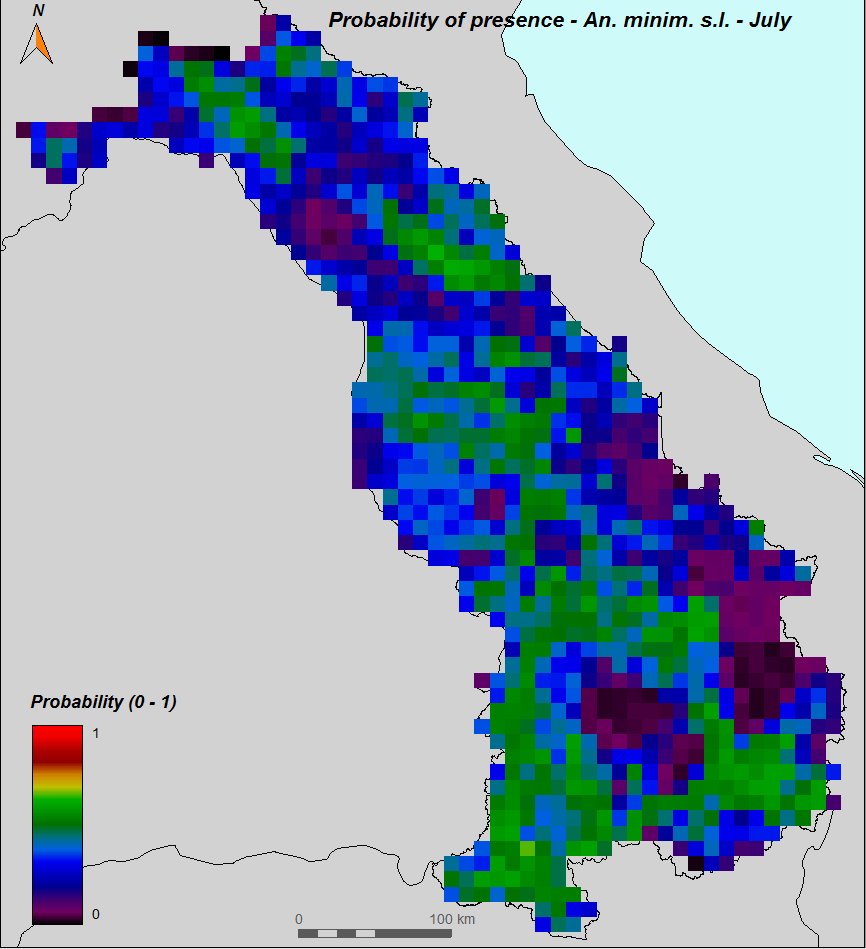

Supplement: S1 Fig — (ZIP) [file pone.0177274.s002.zip › Y4_Minim_07.tif]

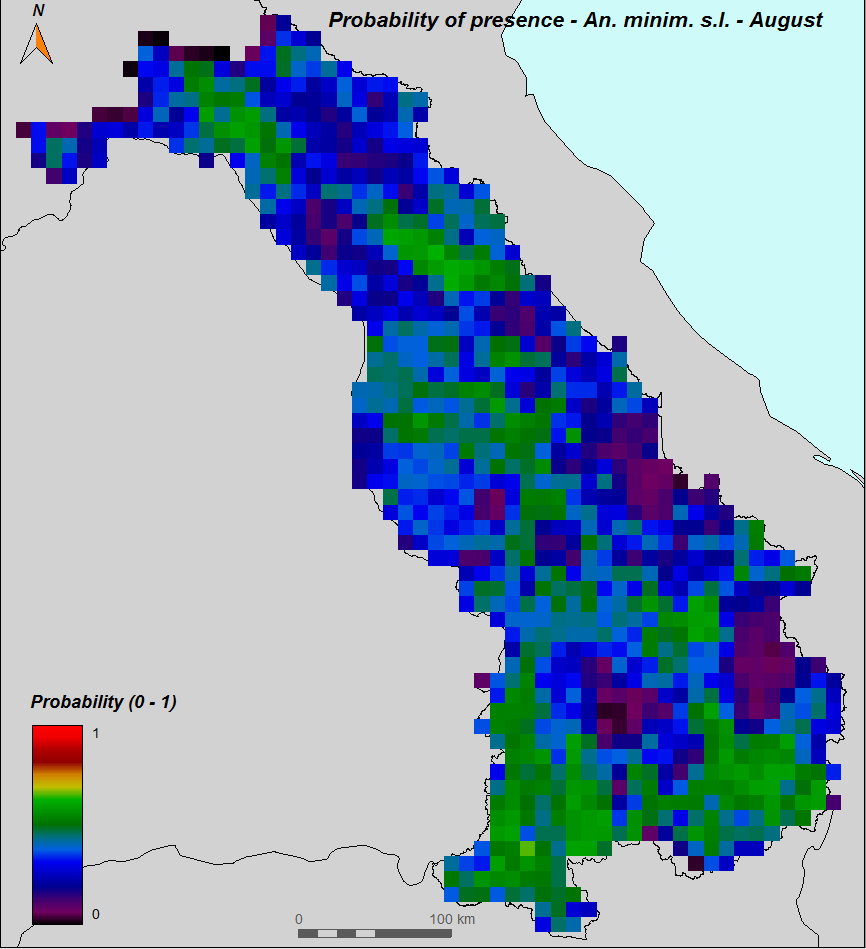

Supplement: S1 Fig — (ZIP) [file pone.0177274.s002.zip › Y4_Minim_08.tif]

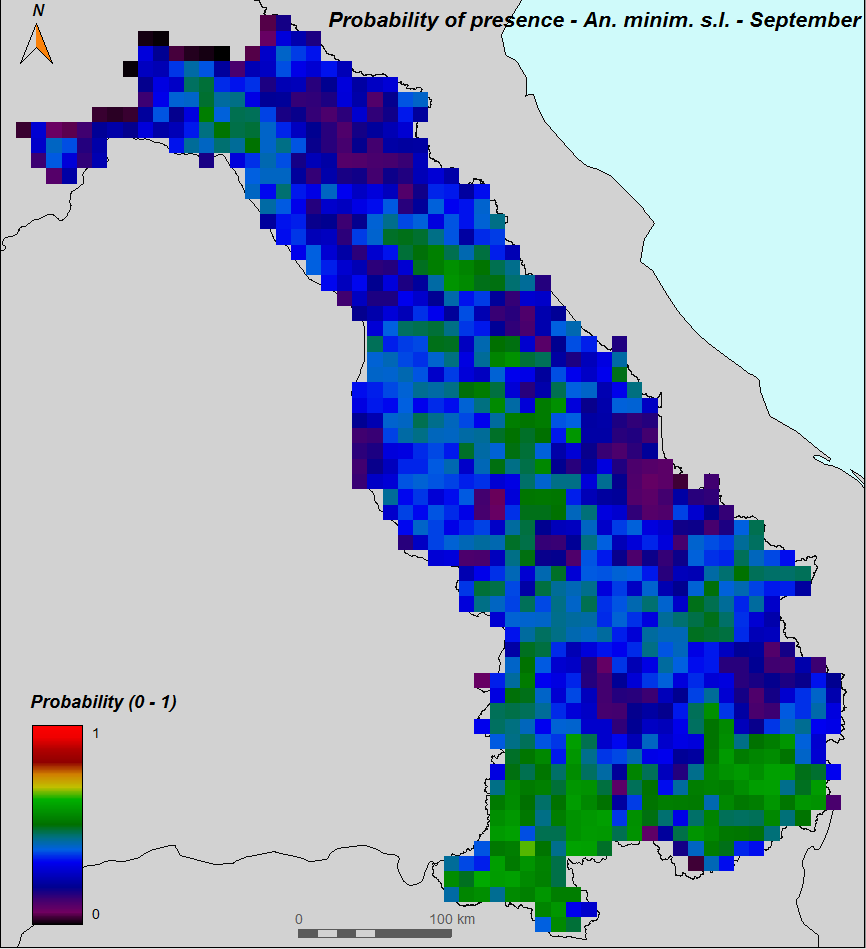

Supplement: S1 Fig — (ZIP) [file pone.0177274.s002.zip › Y4_Minim_09.tif]

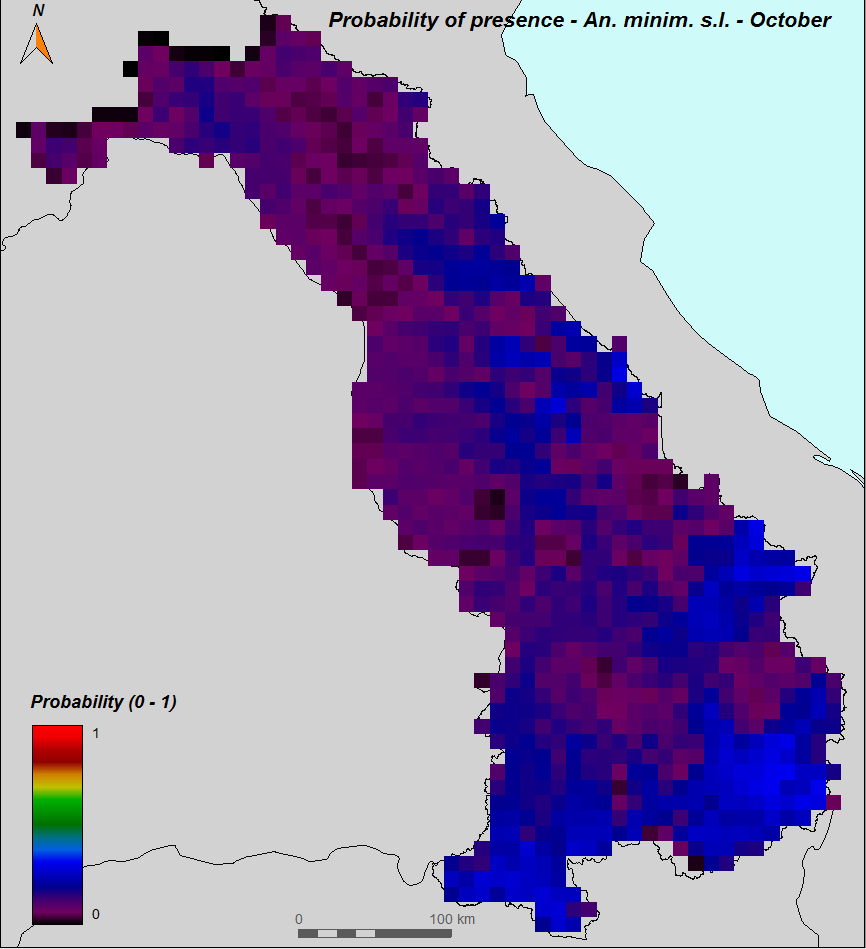

Supplement: S1 Fig — (ZIP) [file pone.0177274.s002.zip › Y4_Minim_10.tif]

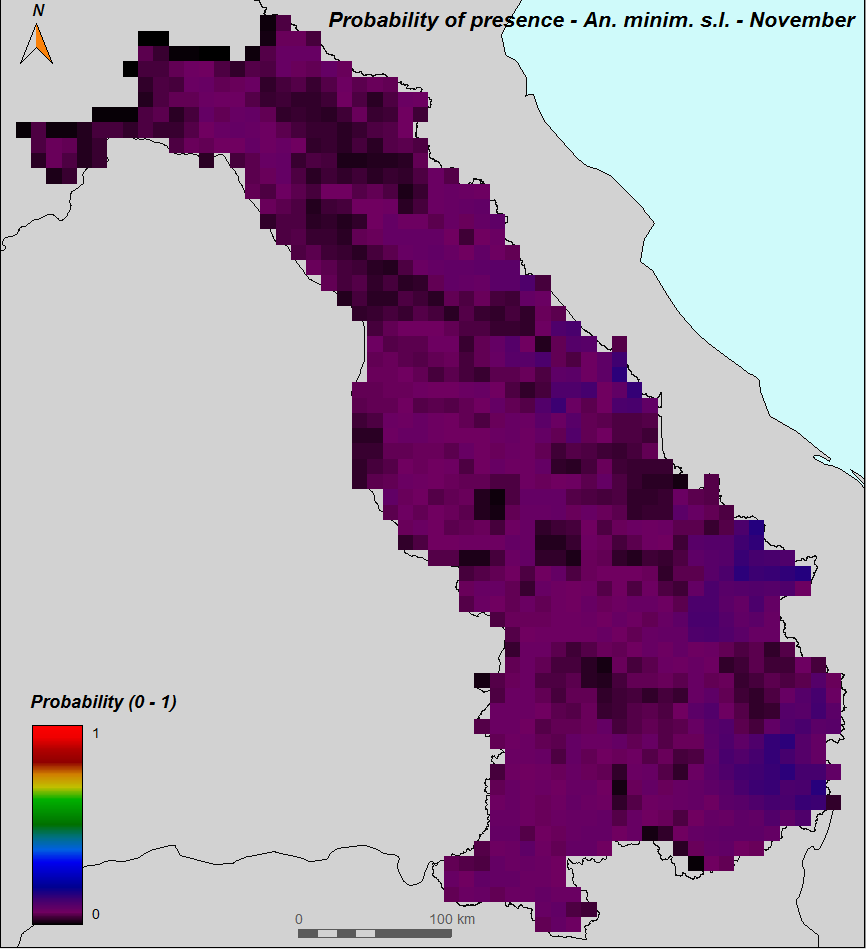

Supplement: S1 Fig — (ZIP) [file pone.0177274.s002.zip › Y4_Minim_11.tif]

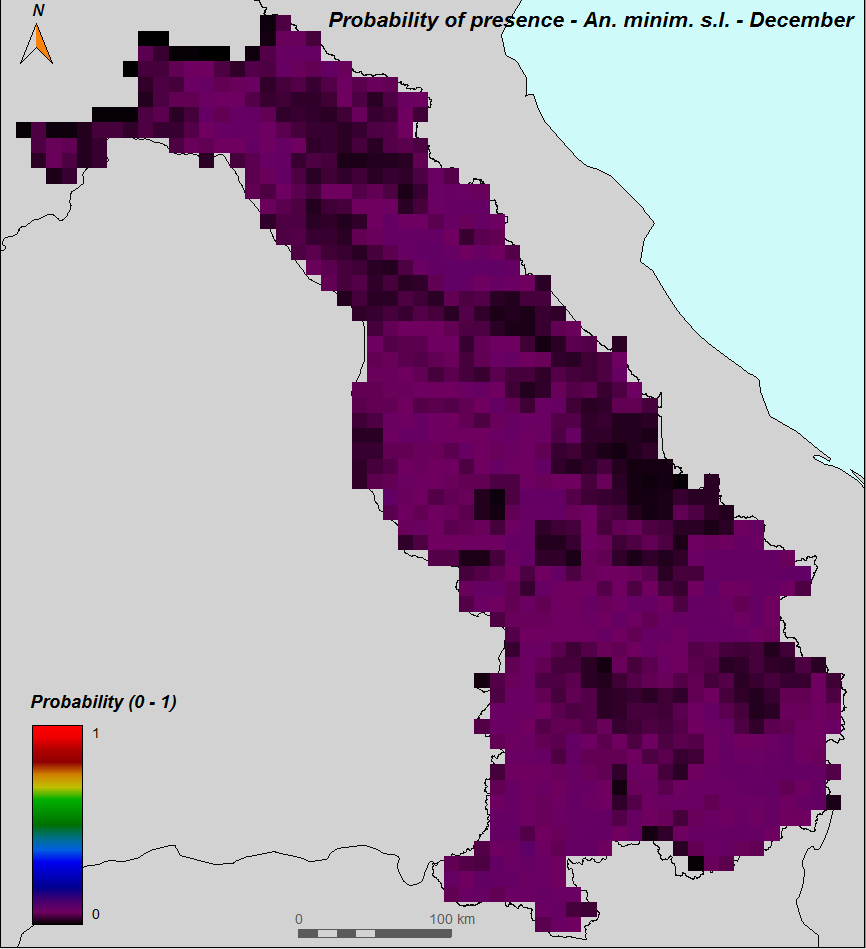

Supplement: S1 Fig — (ZIP) [file pone.0177274.s002.zip › Y4_Minim_12.tif]
